# Probing Bioinorganic Electron Spin Decoherence Mechanisms with an Fe_2_S_2_ Metalloprotein

**DOI:** 10.1021/acs.jpcb.4c06186

**Published:** 2024-10-11

**Authors:** Christian A. Totoiu, Alec H. Follmer, Paul H. Oyala, Ryan G. Hadt

**Affiliations:** Division of Chemistry and Chemical Engineering, Arthur Amos Noyes Laboratory of Chemical Physics, California Institute of Technology, Pasadena, California 91125, United States

## Abstract

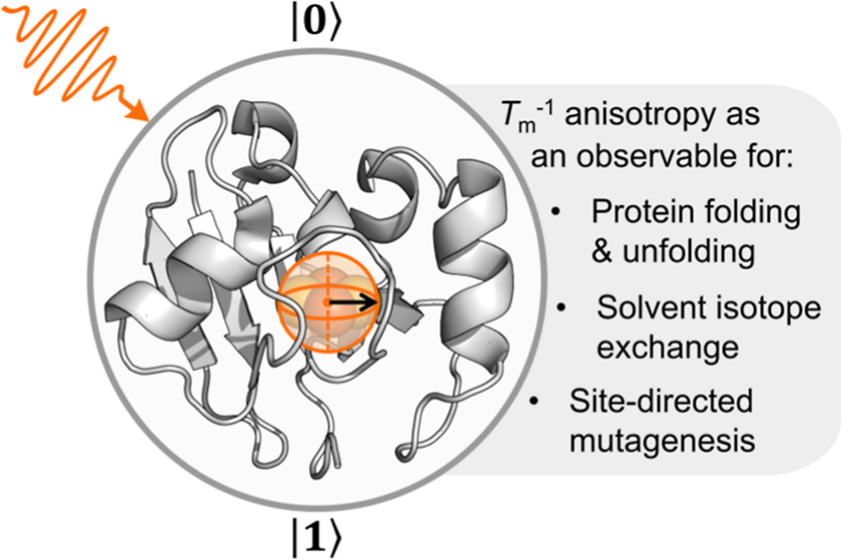

Recent efforts have sought to develop paramagnetic molecular quantum bits (qubits) as a means to store and manipulate quantum information. Emerging structure–property relationships have shed light on electron spin decoherence mechanisms. While insights within molecular quantum information science have derived from synthetic systems, biomolecular platforms would allow for the study of decoherence phenomena in more complex chemical environments and further leverage molecular biology and protein engineering approaches. Here we have employed the exchange-coupled *S*_T_ = 1/2 Fe_2_S_2_ active site of putidaredoxin, an electron transfer metalloprotein, as a platform for fundamental mechanistic studies of electron spin decoherence toward spin-based biological quantum sensing. At low temperatures, decoherence rates were anisotropic, reflecting a hyperfine-dominated decoherence mechanism, standing in contrast to the anisotropy of molecular systems observed previously. This mechanism provided a pathway for probing spatial effects on decoherence, such as protein vs solvent contributions. Furthermore, we demonstrated spatial sensitivity to single point mutations via site-directed mutagenesis and temporal sensitivity for monitoring solvent isotope exchange. Thus, this study demonstrates a step toward the design and construction of biomolecular quantum sensors.

## Introduction

Molecular quantum information science (QIS) seeks to utilize the quantum properties and tunability of molecular systems for computation, communication, and sensing applications.^1^ The fundamental unit of quantum information is the quantum bit (qubit), a complex-valued superposition of a formal two-level quantum system. Qubits have been physically realized, for example, with superconducting loops, trapped ions/atoms, and silicon quantum dots.^2^ However, these technologies require millikelvin temperatures.

Unpaired electrons also satisfy the criteria for a qubit. As such, anionic nitrogen vacancy (NV^–^) centers and molecular electron spins provide a platform for site-specific quantum coherence on an atomic scale; they can further exhibit favorable coherence properties up to room temperature. This has led to tantalizing quantum sensing applications. For example, NV^–^ centers have been used for atomic scale imaging,^3^ nanoscale thermometry,^4^ imaging of magnetic fields in live bacteria,^5^ monitoring single neuron action potentials,^6^ and studying condensed matter magnetism.^7^ While providing significant advances, the coherence properties of NV^–^ centers are fixed by the diamond lattice, which is also physically bulky and can be difficult to surface functionalize. Quantum sensing with molecular electron spins, however, may provide advantages such as tunable coherence properties, greater spatial resolution, and the ability to probe more complex chemical phenomena.

**Figure 1:**
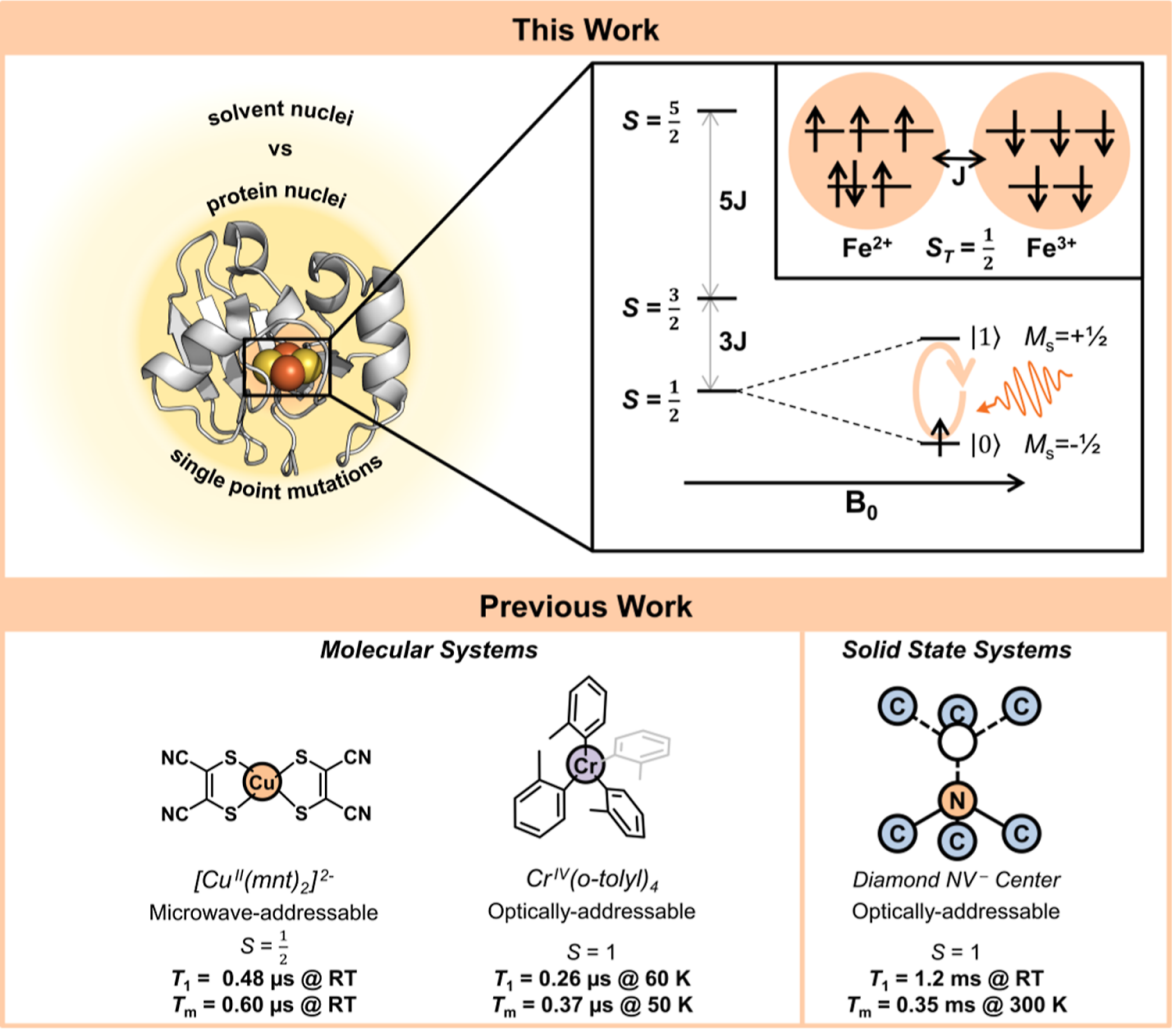
QIS in molecular and biomolecular systems. (Top) The *S*_T_ = 1/2 ground state of the ISC in Pdx functions as a spin qubit for biological quantum sensing [PDB: 1XLQ]. (Bottom) Previous works in molecular QIS have probed decoherence in synthetic systems as models of NV^–^ centers.

Quantum sensing based on electron spins requires mechanistic understanding of decoherence. Briefly, an applied magnetic field splits the electron spin *M*_S_ sublevels, a coherent superposition between which can be formed using microwave pulses in an electron paramagnetic resonance (EPR) spectrometer (Figure 1). The coherence lifetime is controlled by two time constants: *T*_1_ and *T*_2_. The spin–lattice relaxation (*T*_1_) corresponds to longitudinal relaxation due to absorption or stimulated emission of phonons and is highly sensitive to temperature.^8^ Spin–spin relaxation (*T*_2_) corresponds to transverse relaxation due to paired flip-flops of coupled spins.^8^ This latter parameter constitutes the system’s decoherence time, dictating the duration quantum information can be stored. Given *T*_1_ represents an upper bound on *T*_2_, significant efforts have been made to prolong *T*_1_ to increase the temperature at which *T*_2_ can be maintained.^9−14^ In pulse EPR, the phase memory time (*T*_m_) offers an empirical parameter that incorporates *T*_2_, as well as other contributions such as instantaneous diffusion and lifetime broadening. In regimes where spin flips dominate, *T*_m_ can be used as a proxy for *T*_2_. Thus, *T*_m_ variation due to local environmental effects represents a quantum sensing modality.

Biological systems are attractive targets for studying spin relaxation phenomena. Significant synthetic efforts have been made to probe decoherence in molecular qubit candidates. Carrying out similar efforts with biological systems offers some advantages, as (1) they are inherently biologically compatible/integrable and would constitute potentially powerful quantum sensing modalities, (2) the more complex structures of biological macromolecules provide a robust platform for gaining fundamental mechanistic insight into electron spin decoherence, beyond what is obtainable using molecular synthesis, and (3) biomolecular qubits offer more streamlined tuning strategies via conventional molecular biology techniques. Such mechanistic studies benefit greatly from the large body of literature on the EPR of biological systems.

Here we provide a mechanistic study to demonstrate a decoherence-based biological quantum sensing approach using ferredoxins, a class of electron transfer metalloproteins involved in processes such as photosynthesis, nitrogen fixation, and mitochondrial function.^15−17^ Iron–sulfur clusters were discovered and characterized by EPR in 1960, and the first EPR spectra of ferredoxins were reported in 1966.^18−21^ Ferredoxins have been previously demonstrated to act as qubits with decoherence times sensitive to nitrogen isotope labeling.^22^ Additionally, pulse EPR-monitored redox potentiometry at Q-band has been utilized to determine midpoint potentials of multiple plant-type ferredoxin isoforms.^23^ We have utilized putidaredoxin (Pdx), a bacterial ferredoxin from *Pseudomonas putida*,^24^ as it features established protocols for production, purification, and site-directed mutagenesis, and its Fe_2_S_2_ iron–sulfur cluster (ISC) has been characterized by magnetic spectroscopy.^25−27^ Upon reduction, the ISC features an *S*_T_ = 1/2 ground state, which functions as an effective biomolecular qubit (Figure 1^9,28−31^). Using a range of established pulse EPR methods coupled to isotope and chemical perturbations, the decoherence mechanism in Pdx at low temperatures was determined to be hyperfine-dominated, analogous to the decoherence mechanism of NV^–^ centers, yet also distinct relative to previous observations for molecular systems. This provided the ability to distinguish between isotopic contributions from the protein and surrounding solvent. The *T*_m_ quantum sensing mechanism was also sensitive to protein-based single point mutations introduced via site-directed mutagenesis. Thus, this study demonstrates the utility of biomolecular systems for fundamental mechanistic studies of spin relaxation phenomena in nuclear spin-rich environments and the development of coherent quantum systems for applications in QIS.

## Methods

### Expression

Overnight cultures were initiated in LB media with ampicillin and plasmid glycerol stock then grown (37 °C). Expression cultures were started with 2xYT media, ampicillin stock solution, and overnight culture. The expression cultures were grown for several days at room temperature then harvested by centrifugation.

### Mutagenesis

The WT Pdx plasmid was ordered in a pET-17b plasmid. Pdx mutants were prepared with a Q5 Site-directed Mutagenesis Kit with the NEBaseChanger primer design tool, and sequences were confirmed via Sanger sequencing. Plasmids were transformed into the *E. coli* BL21(DE3) cell line. The Pdx Q88G plasmid was transformed into the *E. coli* C43(DE3) cell line.

### Purification

Cell pellets were resuspended in buffer. Soluble protein was isolated by sonication then ultracentrifugation. The supernatant was decanted and purified by anion exchange chromatography. Fractions were eluted by increasing KCl concentration and collected. Fractions containing Pdx (deduced by a brown color of oxidized Pdx) were combined and concentrated.

### UV–Vis and Circular Dichroism (CD)

Protein concentration was quantified by electronic absorbance (UV–Vis) spectroscopy via eq S1. Circular dichroism spectra were collected for WT Pdx in buffer and under mild denaturing conditions.

### Urea Gradient

Samples were prepared of WT Pdx in sample buffer containing urea to final concentrations of 0, 1, 2, or 4 M. WT Pdx stock solution was diluted with buffer containing urea then incubated for 30 min at room temperature. Sodium dithionite in sample buffer was added for reduction. Samples were transferred to EPR tubes and flash-frozen in liquid nitrogen. Final WT Pdx concentrations were 200 μM.

### Mutants

Pdx mutants were buffer exchanged into sample buffer and concentrated. Protein concentrations were quantified via UV–Vis with eq S1. Samples were prepared for each protein variant and reduced with sodium dithionite. Final concentrations were WT Pdx at 200 μM, Pdx G41R at 200 μM, and Pdx Q88G at 79 μM.

### Deuteration

WT Pdx stock was diluted in deuterated buffer and incubated on ice or at 4 °C for varying durations. A control was prepared with the WT Pdx stock diluted in nondeuterated buffer. Reduction was performed with sodium dithionite, and samples were transferred to EPR tubes and flash frozen in liquid nitrogen. Samples were 100–150 μL with 200 μM WT Pdx. The Pdx in H_2_O buffer control and the Pdx in D_2_O buffer (*t* = 48 h.) samples were analyzed by three-pulse ESEEM and HYSCORE at 15 K.

### EPR

X-band continuous-wave (CW) EPR (Bruker EMX) spectra were collected for each sample. For X-band pulse EPR spectroscopy, a Bruker ElexSys E580 pulse EPR spectrometer was used, equipped with an MD4 dielectric ENDOR resonator. Temperature control was achieved using a ColdEdge closed-loop cryogen-free cryostat. Two-pulse echo-detected field-swept spectra and two-pulse (Hahn echo) decays were collected using a π/2−τ–π–echo pulse sequence. Field-swept spectra were used to determine the perpendicular and parallel field positions used to collect the two-pulse decays (π/2 = 8 ns). The spectra were collected at temperatures of 10, 15, and 20 K. Two-pulse decays were fit using a MATLAB script and were fit to the conventional phase-memory relaxation expression (eq S2). The fit exponential was subtracted from the data followed by apodization with a positive Hamming window (“ham+”) and zero-filling (8-fold filling), and a fast Fourier transform was taken.

## Results

To gain mechanistic insight related to quantum sensing with a metalloprotein active site, we sought to measure variations in ISC *T*_m_ times upon systematic perturbations to the chemical microenvironment surrounding the Pdx active site, including urea-induced protein unfolding, buffer deuteration, and site-directed mutagenesis.

### Hyperfine-Dominated Decoherence in Putidaredoxin

Wild-type (WT) Pdx was expressed, purified, and characterized as described in the Methods and Section SI ISC reduction provides an *S*_T_ = 1/2 ground state with *g* = [1.922, 1.937, 2.022] displaying a nearly axial line shape with *g*_⊥_ = 1.929(5) and *g*_||_ = 2.022 (Figures S10 & S36 and Tables S2 & S5), consistent with previous reports.^32−34^ As detailed in the Methods and Section SI, pulse EPR was utilized to measure *T*_m_ times under various conditions.

**Figure 2:**
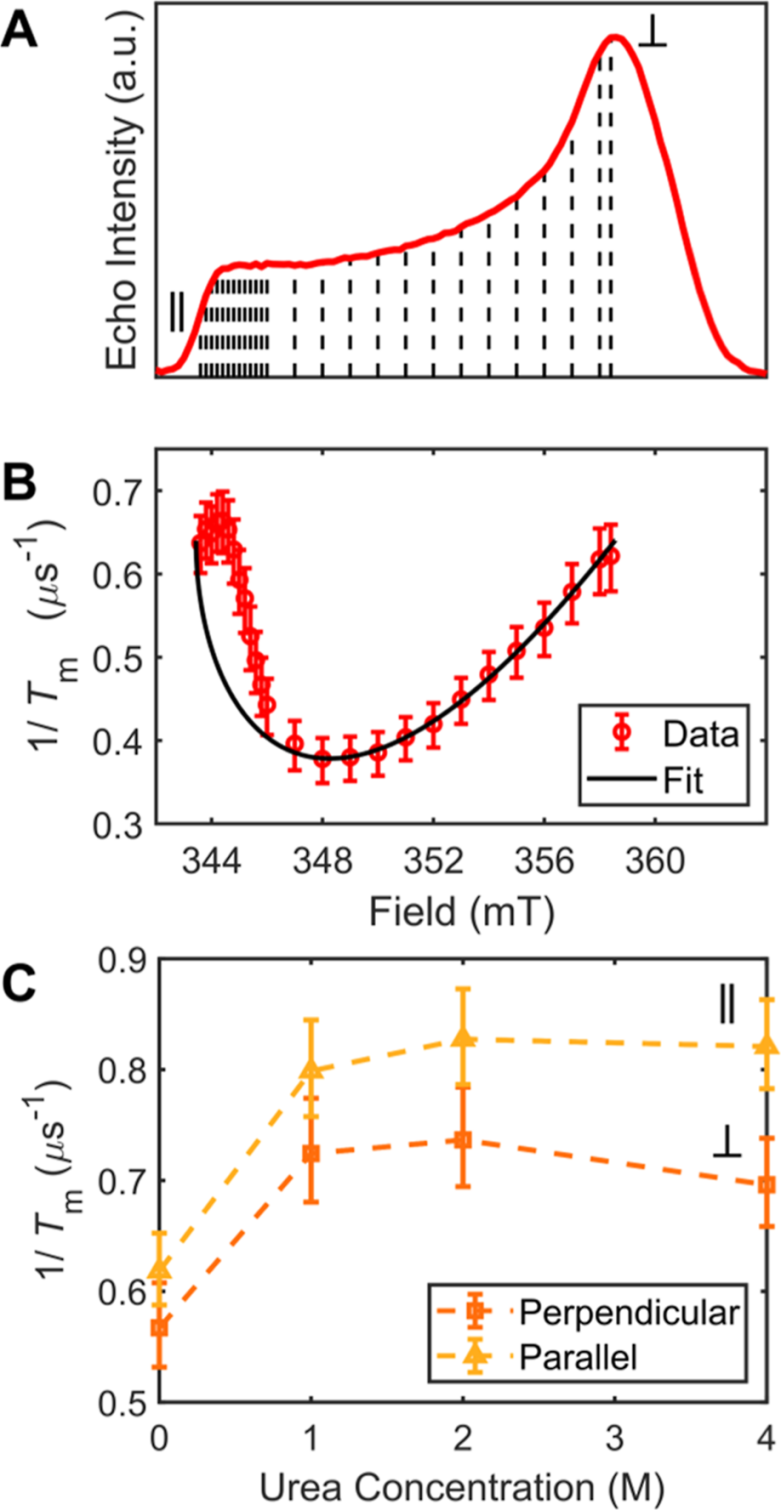
Hyperfine-dominated decoherence in WT Pdx. (A) Two-pulse echo-detected field sweep for WT Pdx collected at 15 K. Black, vertical dashed lines indicate field positions at which Hahn echo decays were collected. (B) Field dependence of *T*_m_^–1^ for WT Pdx collected at 15 K with 95% confidence intervals. Data shown as red circles with fit in black. (C) Experimental *T*_m_^–1^ data (10 K) for WT Pdx collected with a urea gradient (0–4 M) for both canonical positions (perpendicular in orange squares, parallel in yellow triangles).

From the X-band (∼9.730 GHz) echo-detected field-swept spectrum at a temperature of 15 K (Figure 2A), the canonical parallel and perpendicular field positions were 343.6 and 358.4 mT, respectively, consistent with CW-EPR (Figure S36). To gain insight into the decoherence mechanism, *T*_m_ was measured at 0.2 mT increments from 343.6 to 346.0 mT and at 1 mT increments from 346.0 to 358.0 mT (Figure 2B). The decoherence rate (*T*_m_^–1^) was anisotropic, ranging from ∼0.38 to 0.66 μs^–1^ for a ratio of ∼1.7. We note here that very similar absolute values (within 2.5%) were obtained from different protein sample batches/preparations (Section SVII). The field dependence of *T*_m_^–1^ exhibited a concave up behavior, maximizing near the canonical positions and minimizing at 348.0 mT. Upon transformation to angular coordinates, the minimum *T*_m_^–1^ corresponded to a position of ≈57° (1 rad) from the perpendicular plane (Section SII.A & Figure S5).

This angular behavior closely follows the dipolar coupling averaged across all orientations (⟨ω⟩_θ_) (Section SII.A)
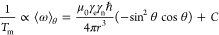
1where μ_0_ is the permeability of free space, γ_e_ is the electron gyromagnetic ratio, γ_n_ is the nuclear gyromagnetic ratio, ℏ is the reduced Planck constant, *r* is the electron–nuclear distance, θ is the angle between the electron–nuclear vector and the applied magnetic field, and *C* is an integration constant. The experimental field-dependent *T*_m_^–1^ could be partially fit to this −sin^2^θ cos θ dependence (Figure 2B, black line), matching well near the perpendicular positions and at intermediate field positions, and deviating slightly near the parallel orientation. This deviation is discussed further in Section SII.A. Additional data points collected at fields greater than the perpendicular orientation (Figures S47–S49) further reveal a decrease in *T*_m_^–1^, qualitatively in agreement with the hyperfine-dominated contributions to decoherence.

Consistent with this mathematical functional form, the minimum experimental *T*_m_^–1^ was close to the magic angle, 54.7°, at which dipolar coupling vanishes to zero from the angular dependence of the anisotropic hyperfine interaction^8^
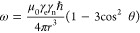
2

Thus, the *T*_m_^–1^ anisotropy reflects a hyperfine-dominated decoherence mechanism in Pdx, contrasting previous observations in molecular systems (*vide infra*, Discussion).^28,29,35,36^

### Approximation and Measurement of WT Pdx Decoherence and Sensitivity to Protein Folding

*T*_m_^–1^ can be used to detect changes in nuclear spin density. The nuclei that contribute to decoherence are located beyond the spin-diffusion barrier. The spin-diffusion barrier is the sphere of strongly coupled nuclear spins surrounding an electron spin. This coupling is too strong to enable decoherence. Proximal (<4–7 Å) nuclei can reside inside of the spin-diffusion barrier and, thus, do not largely contribute to decoherence.^37^ Beyond that barrier, nuclei residing in the shell between 4–14 Å from the electron spin can contribute to hyperfine-mediated decoherence. At distances longer than ∼14 Å, the inverse cubic radial dependence of the dipolar component of the hyperfine coupling will likely lead to diminishing changes to *T*_m_^–1^.^37,38^ At intermediate distances for Pdx, two domains can contribute to decoherence: the intramolecular protein and intermolecular solvent microenvironments. Considering these two influences, their relative effects on *T*_m_^–1^ can be approximated by^39−41^
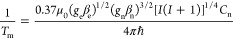
3where *g*_e_ is the electron *g*-factor, β_e_ is the Bohr magneton, *g*_n_ is the nuclear *g*-factor, β_n_ is the nuclear magneton, *I* is the nuclear spin number, and *C*_n_ is the number density of nuclear spins.^8^ Considering only protons and other spin-active nuclei,^39^*T*_m_^–1^ rates of 0.258 and 0.446 μs^–1^ were predicted for protein and solvent, respectively. Notably, these rates are on the same order of magnitude as the average/isotropic experimental value of 0.583 μs^–1^ at 10 K (Figure 2C, Section SII.B & IV).

Equation 3 features two assumptions. First, that abundant protons dominate decoherence. In biomacromolecules and their aqueous buffers at low temperature, this assumption is reasonable. Second, the protons are arranged in a cubic lattice. Though this assumption does not hold in frozen H_2_O due to the hydrogen-bonding lattice, eq 3 nevertheless provided a simple algebraic expression and adequate estimates for *T*_m_^–1^ in Pdx vs experimentally measured values. This mixed contribution may be represented through weighting the protein (*T*_m,p_^–1^) and solvent (*T*_m,s_^–1^) rates to the total decoherence rate with a solvent contribution factor (ϵ_s_)
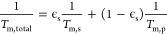
4Importantly, the solvent (ϵ_s_) and protein (1 – ϵ_s_) contribution factors cannot be taken as their volume fraction of the shell of influence on decoherence due to the dipolar coupling’s inverse cubic radial dependence (*r*^–3^).

Such an analysis would suggest decoherence in Pdx may derive from varying contributions from both protein and solvent nuclei. Calculations with eq 3 indicate that the decoherence rate from full solvent exceeds that for full protein due to the greater proton concentration in solvent (Section SII.B). For proteins with partial specific volumes of 0.7–0.76 cm^3^/g, the solvent rate exceeds the protein rate by 15–25%. To experimentally probe these relative contributions to *T*_m_^–1^, Pdx was mildly denatured (Section SIV). Upon addition of 1 M urea, the average *T*_m_^–1^ increased from 0.583 to 0.747 μs^–1^ (Figure 2C), an increase of 28%. The magnitude of increase is similar to the calculated increase in rate from protein to solvent contribution to decoherence (Section SII.B). In addition to these observations, UV CD spectra exhibited decreasing α helix and β sheet secondary structure and increasing random coil secondary structure (Figure S11). EPR spectral changes were also minimal, consistent with a previous study, and indicative of minimal perturbation of the *g* values and, thus, exchange coupling constant (*J*) (Figure S10 & Table S2).^42^ Further addition of urea resulted in more modest changes to *T*_m_^–1^ (Figure 2C).

In summary, *T*_m_^–1^ in Pdx is dominated by electron–nuclear spin hyperfine couplings with protons and is modeled qualitatively by eq 3. *T*_m_^–1^ also reflects both contributions from the protein and solvent accessibility. Because of this, *T*_m_^–1^ also depends on the conformational state of the protein.

### Probing the Effect of ISC Active Site Solvation on Decoherence

As demonstrated above, ISC decoherence is sensitive to macroscopic changes in the nuclear spin bath comprised of both the protein and solvent. Herein, we examined the role of buffer and protein isotope substitution to further probe spin bath contributions to *T*_m_^–1^. The substitution of hydrogens for deuterons has been extensively used to elongate *T*_m_ for advanced pulse EPR techniques, such as distance measurements by double electron–electron resonance (DEER).^43^ Deuterated electron spin–echo envelope modulation (ESEEM) and electron–nuclear double resonance (ENDOR) demonstrated water displacement near a metalloprotein active site.^44^ For Pdx, exchange of hydrogens for deuterons provides a kinetic process by which temporal changes in the decoherence rate can be monitored via perturbation of the nuclear spin bath.

Solvent deuteration was performed by dilution (∼1:10) of a protein stock solution into buffer composed of deuterated water (D_2_O) and 10% glycerol (C_3_D_8_O_3_). Samples were incubated for durations of 1 min to 48 h, then flash frozen.

**Figure 3:**
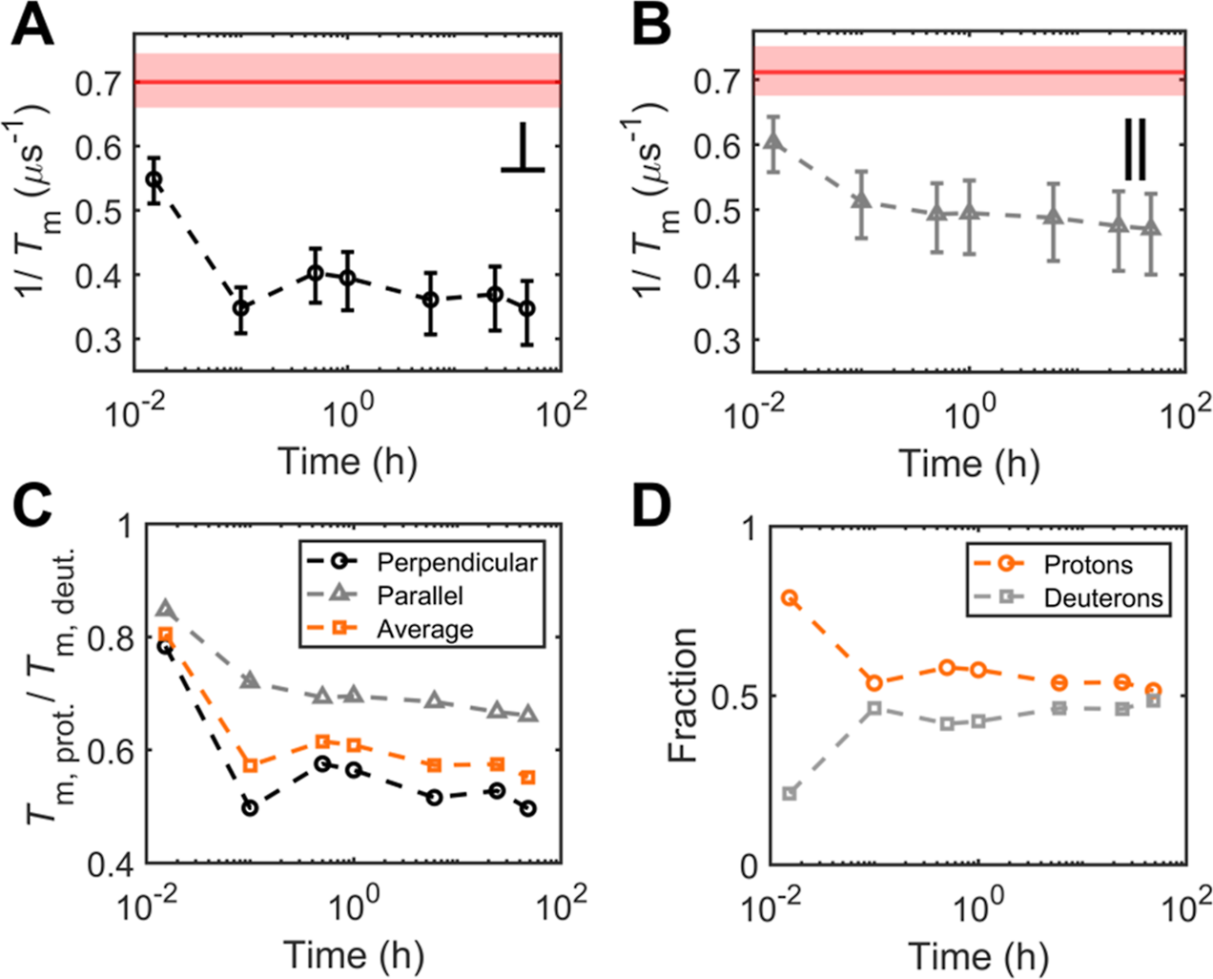
Effect of partial solvent deuteration on WT Pdx decoherence. (A,B) Decoherence rates for WT Pdx samples in protonated buffer (red line) and deuterated buffer with increasing incubation times at the (A) perpendicular (black circles) and (B) parallel (gray triangles) field positions at 15 K with 95% confidence intervals. (C) Deuterated sample *T*_m_^–1^ values normalized by the nondeuterated sample *T*_m_^–1^ value at the perpendicular and parallel field positions vs the incubation time. The calculated average (isotropic) decoherence rate is included (orange squares). (D) The fractional contributions to decoherence rate from protons (orange circles) and deuterons (gray squares).

CW-EPR spectra were nearly identical over the different incubation times (Figure S20 & Table S3). However, after 6 min, coherence was prolonged, and *T*_m_^–1^ decreased in an anisotropic fashion by a factor of ∼2 at the perpendicular position and ∼1.5 at the parallel position (Figure 3A,B). Due to the difference in nuclear spins (^1^H *I* = 1/2; ^2^H *I* = 1) and gyromagnetic ratios (^1^H γ = 42.577 MHz T^–1^; ^2^H γ = 6.536 MHz T^–1^) between the hydrogen isotopes, a 13-fold decrease in *T*_m_^–1^ was expected for complete deuteron exchange. The submaximal decrease observed here was likely due to residual protons from dilution with deuterated buffer and incomplete deuteron exchange of solvent-inaccessible hydrogens within the protein. The *T*_m_ increases (and *T*_m_^–1^ decreases) are consistent with studies of nuclear spin distance effects on decoherence in molecular systems.^37,38^

The difference in normalized *T*_m_^–1^ values (relative to the nondeuterated sample) between the perpendicular and parallel positions indicated *T*_m_^–1^ is an anisotropic probe of changes in nuclear spin distribution (Figure 3C). For example, at the perpendicular orientation, *T*_m_^–1^ decreased abruptly within 6 min, then slowly over 48 h. The sudden decrease in *T*_m_^–1^ indicates sensitivity to solvent and fast-exchanging protons. Conversely, at the parallel orientation, *T*_m_^–1^ decreased more gradually with incubation time, indicating greater sensitivity to more slowly exchanging protons, likely those of the protein.

As described in Section SII.B, eq 4 can be similarly applied to rather account for proton and deuteron contributions to decoherence rates
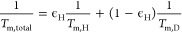
5The proton and deuteron contributions can be related (ϵ_D_ = 1 – ϵ_H_) through the control for *T*_m_^–1^ obtained with natural abundance isotopes. This expression assumes hydrogens overwhelmingly dominate *T*_m_^–1^. Such an assumption is reasonable given the number of hydrogens present and the proton gyromagnetic ratio relative to other spin-active nuclei. After ∼1 min of incubation, *T*_m_^–1^ decreased from 0.70 to 0.57 μs^–1^ due to a change in proton contribution from 100% to 79%, which decreased further to 54% after 6 min. At longer times, the proton and deuteron contributions to *T*_m_^–1^ coalesced toward 50:50. Due to the protein’s embedded active site, these percentage contributions were sensitive to the proportion and positions of exchangeable hydrogens. Note, as indicated in Section SVIII, the 0.70 μs^–1^ value of *T*_m_^–1^ for WT Pdx relative to 0.63 μs^–1^ above is due to the different buffer conditions utilized for the deuteration experiment.

The presence of two canonical field positions provides the capability for enhanced spatial resolution of the exchange process in structurally complex proteins. Different orientations permit varying contributions from different spatially oriented nuclei and, thus, afford additional resolution. In principle, the analysis above may be further extrapolated to account for protons and deuterons from solvent and protein separately (eq S18). However, this would require greater resolution to disambiguate the respective contributions and coefficients due to the nonlinear radial dependence of dipolar coupling.

Following *T*_m_ fitting of the two-pulse decays, fast Fourier transforms of the exponential-subtracted data indicated the presence of protons and deuterons at their respective Larmor precession frequencies with corresponding sum and difference peaks (Figures S27 & S28), consistent with the analysis thus far. However, additional coupled nuclei were also observed at low frequencies. Three-pulse ESEEM and hyperfine sublevel correlation (HYSCORE) spectroscopies indicated coupling to nitrogens in addition to hydrogens (Figures S29–S32 & Table S4). These nitrogens are likely from the Pdx loop of cysteines coordinating the ISC. These relatively strongly coupled nuclei prominent in HYSCORE also likely reside within the spin-diffusion barrier, and do not contribute strongly to decoherence.

In summary, spin-based quantum sensing offers temporal and spatial resolution for monitoring chemical processes, in this case involving isotopic changes. This capability provides mechanistic information for processes occurring near biomolecular quantum sensors.

### Probing the Effect of Single Point Mutations on Decoherence

**Figure 4:**
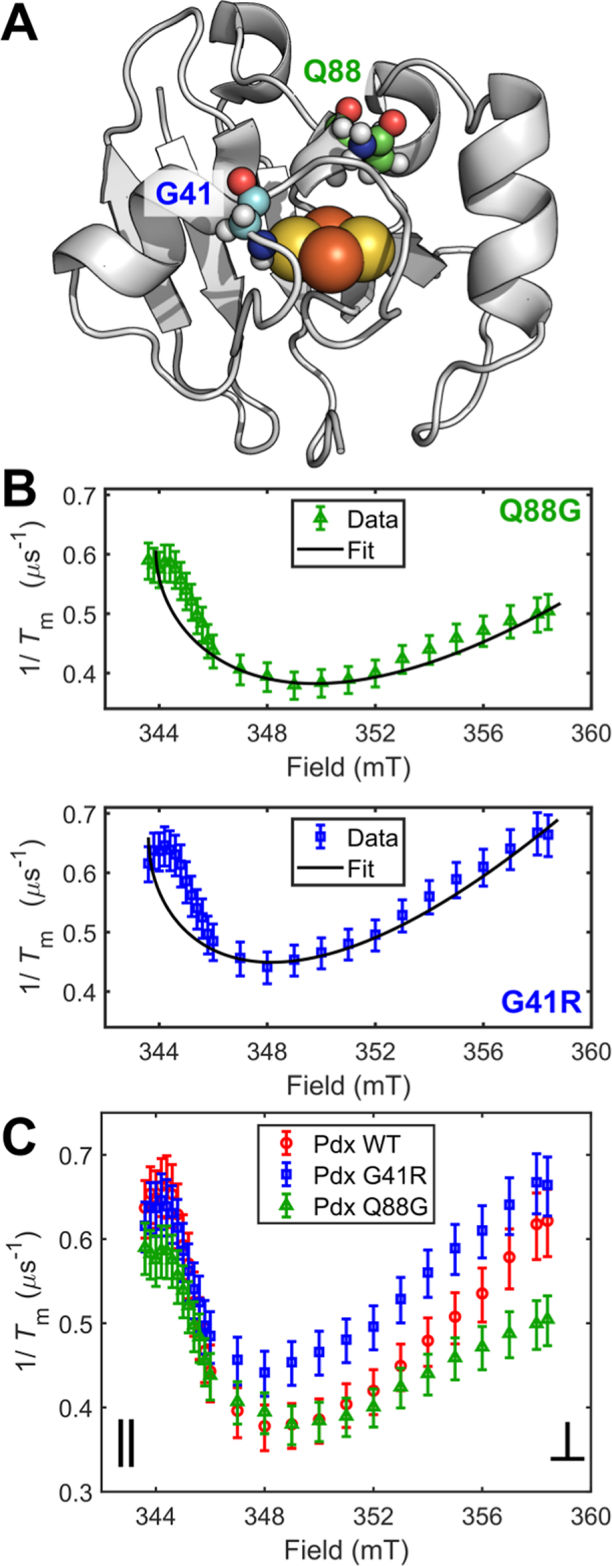
Hyperfine-dominated decoherence in Pdx point mutants. (A) Residue positions relative to the Pdx active site (PDB: 1XLQ). (B) Field dependences of *T*_m_^–1^ of Q88G (green triangles) and Pdx G41R (blue squares) collected at 15 K with accompanying fits. (C) Overlaid field dependences of *T*_m_^–1^ for WT Pdx, G41R, and Q88G collected at 15 K with 95% confidence intervals.

Given the hyperfine-dominated decoherence mechanism, we sought to test the sensitivity of *T*_m_^–1^ to changes in the local nuclear spin bath via site-directed mutagenesis. As a first approach, we targeted point mutations that alter the number and arrangement of spin-active nuclei just outside the spin-diffusion barrier (∼4–14 Å from the center of the ISC, Figure S34).^37,38^ Two mutations were prepared: glycine to arginine at position 41 (G41R) and glutamine to glycine at position 88 (Q88G) (Figure 4). Relative to WT Pdx, G41R added 10 hydrogens while Q88G removed five. It was therefore qualitatively hypothesized *T*_m_^–1^ would increase for G41R and decrease for Q88G.

The CW-EPR spectra of both variants were similar to WT Pdx (Figure S36 & Table S5). Relative to WT, G41R exhibited small changes (*g* = [1.922, 1.937, 2.022] and *g* = [1.926, 1.938, 2.023], respectively), while even smaller changes were observed for Q88G (*g* = [1.922, 1.938, 2.022]). These data suggest only slight structural changes to the ISCs.^33^

While the CW-EPR spectra were similar, both mutations yielded measurable changes in *T*_m_^–1^ (Figure 4C). The field dependence of *T*_m_^–1^ for both mutants retained the −sin^2^θ cos θ dependence and, thus, the hyperfine-dominated decoherence mechanism. Interestingly, point-mutation-induced *T*_m_^–1^ changes were anisotropic in magnitude. At the perpendicular position, *T*_m_^–1^ increased for G41R and decreased for Q88G relative to WT, following the expectation based on the changes to the local spin bath (Figure 4C). However, in the parallel position, *T*_m_^–1^ decreased for Q88G and remained measurably unchanged for G41R relative to WT. Together, the anisotropic *T*_m_^–1^ behavior also seemed consistent with the superior fit of the *T*_m_^–1^ data to the −sin^2^θ cos θ functional form near the perpendicular position rather than the parallel position.

The change in *T*_m_^–1^ for the mutants relative to WT may be due to effects arising from different irons in the ISC. The G41R mutation is located near the solvent-proximal ferrous iron, while the Q88G mutation is located closer to the buried ferric iron.^45−47^ Within the Bertrand–Gayda model,^33^ the iron centers of the reduced ISC affect the *g* values differently. These differences arise from the oxidation state-specific spin projection factors and orbital angular momentum quenching of the partially filled iron d orbitals. Thus, proximity to either iron center may affect the *g* values differently and may exert variable effects on *T*_m_^–1^ at the corresponding field positions. Relative to WT, the Q88G mutation yielded an increase in the Coffman parameter^48^ (χ = *g*_y_ – *g*_x_), converse to the decrease from the G41R mutation. The Q88G mutation was proximal to the ferric (buried) iron, which has a larger impact on the Fe_2_S_2_*g* values. Similarly, the *T*_m_^–1^ rates of Q88G varied more from those of WT at the canonical positions. Conversely, the G41R mutation yielded a lesser effect on *T*_m_^–1^ near the canonical positions, and the mutated residue was nearer to the ferrous (peripheral) iron. In this way, the proximity of the mutated residues relative to the individual iron sites appears to impact the anisotropic decoherence rates.

In summary, *T*_m_^–1^ was sensitive to changes in the nuclear spin bath introduced via single point mutations near the ISC. Furthermore, the position of the mutated residue yields variable impacts on the anisotropic *T*_m_^–1^, potentially due to differential contributions from ferric vs ferrous iron centers in the ISC. Additional mutations at varying positions and orientations relative to the active site are the subject of an ongoing study.

## Discussion

To date, the field of molecular QIS has sought to develop synthetic systems with controllable, atomic scale coherence properties for sensing, computation, and information processing. While the scope of potential applications has yet to be determined, research in molecular QIS has contributed significantly to our fundamental understanding of the quantum dynamics of electron spins. For example, by isolating an electron spin in a V(IV) complex from the nuclear spin bath, millisecond coherence could be achieved at 10 K, longer than the NV^–^ center at a comparable temperature.^49,50^ Additional studies provided insight into which nuclear spins are most important for decoherence, defining the spin-diffusion barrier.^37,38^ Synthetic efforts have further developed multiqubit systems for implementing quantum gates and to create controllable arrays of qubits.^51^ Recently, the NV^–^ center electronic structure was emulated in molecular Cr(IV) complexes, endowing them with optical addressability.^28,29^ To alleviate the cumbersome temperature requirements on exploiting desirable quantum coherence properties, models have been developed to understand the spin-vibrational coupling contributions driving *T*_1_, a significant limitation on *T*_m_ at elevated temperatures.^12,52−55^

In this work, we have sought to expand the platforms for fundamental studies within molecular QIS from systems created via synthetic chemistry to biomolecular systems constructed from biological macromolecules–in this case a metalloprotein. This study utilizes metalloprotein-based electron spins for biological quantum sensing. The resulting behavior constitutes a sensor type affected by the presence and orientations of local nuclear spins relative to a paramagnetic center. Such sensors offer new functionalities, enhanced resolution, and novel sensing targets for accessing local chemical and biological microenvironments.

By leveraging *T*_m_ anisotropy, we could experimentally probe the decoherence mechanism in Pdx and discovered it to be hyperfine-dominated. The concave up *T*_m_^–1^ anisotropy observed for Pdx and its variants (Figures 1 and 4) was qualitatively different relative to molecular systems. The *T*_m_^–1^ anisotropy followed a derived functional form from the random orientation of nuclear spins, with slight deviations near the parallel canonical position. A previous study demonstrated a concave down behavior in molecular systems, which was interpreted to arise due to molecular motion (i.e., librational modes) dominating the decoherence mechanism.^36^ Notably, the concave down pattern was observed even in a nuclear spin-rich solvent environment such as that probed here with Pdx. While future biomolecular *T*_m_ anisotropy studies will be useful, these results are tentatively interpreted as deriving from protein constraints on the *S*_T_ = 1/2 ISC active site—akin to the entatic/rack state^56−59^—which may prohibit contributions to decoherence from rotational motion and librations. Through the hyperfine-dominated decoherence mechanism, we could also demonstrate that *T*_m_^–1^ features contributions from both protein and solvent nuclear spins, which was further confirmed and probed through protein unfolding experiments. These results foreshadow the ability to detect protein binding (*T*_m_^–1^ approaching the protein-only contribution) and unfolding (*T*_m_^–1^ approaching the solvent-only contribution). Additionally, localization of such sensors within the cell and near different organelles and biological structures has the potential to vary the local hydrogen concentration and, thus, *T*_m_^–1^ of single protein molecules.

Solvent deuteration further demonstrated incubation time-dependent changes in *T*_m_^–1^ for temporal sensitivity over the course of several minutes to hours. Substitution of hydrogens for deuterons has been previously used to extend protein *T*_m_ for advanced pulse EPR techniques, such as DEER and ENDOR.^43^ This change in *T*_m_ arises from the difference in gyromagnetic ratios between the ^1^H and ^2^H nuclear spins. Here, the *T*_m_^–1^ changed due to the mixing of deuterated buffer upon sample dilution and the exchange of labile protein protons with deuterons. By affecting *T*_m_^–1^ at resonant fields of the perpendicular and parallel ISC orientations differently, the orientation-dependence engendered a mechanism for monitoring spatial effects. Whereas the parallel orientation contains only the *z*-axis, the perpendicular orientation contains both the *x*- and *y*-axes. In this way, labile protons exchanged to deuterons yielded a greater *T*_m_^–1^ change in the perpendicular than the parallel orientation, demonstrating that chemical changes over time can be monitored with accompanying spatial information. Controlling the presence and patterning of nuclear spins near a paramagnetic center has demonstrated significant *T*_m_ changes in molecular V(IV) qubits.^50,60^ In this study, the mechanistic role of nuclear spins is expanded to yield a methodology for tracking temporal changes in *T*_m_.

Site-directed mutagenesis demonstrated the ability to detect single point mutations via *T*_m_^–1^ with sensitivity and spatial resolution. As evident through these experiments, *T*_m_^–1^ provides an observable handle for biological quantum sensing. The qualitative behavior for these mutants was predicted by changes in the nuclear spin density near the active site. Additionally, the changes in *T*_m_^–1^ relative to WT were anisotropic. These hydrogens’ orientations were relative to the *g*-tensor magic angle, where dipolar coupling vanishes to zero, and the position near the ferric or ferrous iron of the ISC. Such considerations can be used to design ligand scaffolds on transition metal complex qubits with nuclei oriented near the magic angles relative to the orientation of the *g*-tensor. For sensing applications, single point mutants demonstrated both sensitivity (dependent on distance from the irons) and spatial resolution from the dipolar coupling.

As a fundamental mechanistic study into electron spin decoherence in a metalloprotein, this work also highlights areas where future research could improve the prospects of biological quantum sensing. For example, the decoherence measurements reported herein were carried out using an EPR addressable biomolecular qubit at cryogenic temperatures (<20 K) due to rapid Raman-driven *T*_1_ at elevated temperature.^61^ These limitations could be overcome by developing and utilizing room-temperature coherent biomolecular quantum sensors, including endowing them with optical addressability. Room-temperature coherence^9,10,13,62^ and optical addressability^63^ have been accomplished in molecular systems, and these capabilities would need to be transferred to specific biomolecular systems. Further coupling spin dynamics mechanistic studies with structural biology methods such as X-ray crystallography, microscopy, and electron diffraction, will provide high precision insights into decoherence phenomena. Also, while sensitivity to nuclear spins has been useful for select quantum sensing applications, engineering in different decoherence mechanisms may provide the ability to sense a broader range of chemically relevant microenvironmental phenomena, with the ultimate goal of achieving various forms of atomically resolved magnetic resonance imaging. For example, as mentioned above, decoherence in NV^–^ centers has also exhibited sensitivity to electric charges,^64^ magnetic fields,^5^ temperature,^4^ and even intracellular molecular dynamics.^65^ Furthermore, while a native metalloprotein active site has been utilized here, we envision the direct incorporation of molecular spin quantum sensors through protein–ligand interactions/binding or *de novo* protein design will improve the scope of potential applications and fundamental spin dynamics studies.

## Conclusions

This work provides a proof of concept for biological quantum sensing using the decoherence of a paramagnetic metalloprotein. *T*_m_ anisotropy has provided a powerful physical inorganic probe of the decoherence mechanism. In Pdx, the anisotropy reflects electron–nuclear spin hyperfine couplings, a significant change relative to molecular motion in smaller synthetic systems. We hypothesize this change derives from protein-based constraints on the ISC, akin to an entatic/rack state. Given the hyperfine mechanism, we further identified *T*_m_^–1^ could effectively sense distinct changes in the nuclear spin bath, including nuclear spins of the protein vs the surrounding solvent or single point mutations introduced by site-directed mutagenesis. Additional temporal (seconds-to-hours) and spatial quantum sensing sensitivities were also demonstrated. Thus, this study illuminates the utility of biomolecular systems for fundamental mechanistic studies of electron spin decoherence phenomena, as well as the utility of *T*_m_^–1^ as an observable for quantum sensing in biological systems and their surrounding microenvironments.

## Supporting Information Available

Instruments, procedures, methodology for data analysis, expressions for fitting and approximations, continuous-wave EPR spectra, tabulated spin Hamiltonian parameters, pulse EPR traces with fits, ESEEM, HYSCORE, UV–Vis electronic absorption spectra, CD spectra, tabulated *T*_m_ values, and additional plots (PDF)
