# Supplementary material for: Probing Bioinorganic Electron Spin Decoherence Mechanisms with an Fe2S2 Metalloprotein
Source: J Phys Chem B. 2024 Oct 11;128(42):10417–26. doi: 10.1021/acs.jpcb.4c06186 (PMC11514009; doi:10.1021/acs.jpcb.4c06186)
Supplement: Supplementary file 1 — jp4c06186_si_001.pdf [file jp4c06186_si_001.pdf]

Supporting Information

Probing Bioinorganic Electron Spin Decoherence Mechanisms with an Fe_2S_2 Metalloprotein

Christian A. Totoiu[1], Alec H. Follmer[1,‡], Paul H. Oyala[1], Ryan G. Hadt[1,*]

[1]Division of Chemistry & Chemical Engineering, California Institute of Technology, Pasadena, CA, 91125

[‡]Current address: Department of Chemistry, School of Physical Sciences, University of California, Irvine, CA, 92697

*Corresponding Author: rghadt@caltech.edu

Table of Contents

I. Materials & Methods 3

II. Methodology for Calculations 6

A. Hyperfine coupling & molecular motion considerations for T_m^{-1} 6

B. Approximation of T_m 11

III. Hyperfine-dominated Decoherence in Pdx WT 15

IV. Urea Gradient 18

V. Solvent Deuteration 26

VI. Mutants of Pdx 40

VII. Expression Batches of Pdx WT 51

VIII. Tabulated T_m values 53

IX. References 57

I. *Materials & Methods*

Selection of mutants

Pdx mutants were selected from sequence alignment of Pdx to Fe_2S_2 ferredoxins. Sequences were accessed on UniProt on May 10, 2021 and selected for alignment. Five selection criteria were imposed: (1) the sequence was reviewed, (2) the protein binds an iron-sulfur cluster, (3) the protein binds only Fe_2S_2 clusters, (4) four cysteine residues coordinate the cluster, and (5) no sequences were repeated. 629 sequences matched these criteria. From these sequences, the residues flanking the coordinating cysteines were determined, and the frequency of each amino acid per position was determined. Pdx had the second most prevalent amino acid at positions 41, 45, 48, and 88. At these sites, point mutations to the most prevalent amino acid were considered. Mutants G41R and Q88G were selected since the mutations involved a considerable change in steric bulk and, thus, number of nuclei.

Mutagenesis

The wildtype Pdx plasmid was ordered from Genscript in a pET-17b plasmid. Pdx mutants were prepared with a Q5 Site-directed Mutagenesis Kit (New England Biolabs). Primers were designed with the NEBaseChanger application, and sequences were confirmed via Sanger sequencing (Laragen).

Plasmids were transformed into the *E. coli* BL21(DE3) cell line and onto ampicillin LB-Agar plates. The Pdx Q88G plasmid was transformed into the *E. coli* C43(DE3) cell line. After overnight growth, colonies were selected and cultured overnight in LB media. Glycerol stocks were prepared with 500 µL overnight culture and 500 µL 50% glycerol (in ddH_2O) and subsequently stored at -80°C.

Expression

Overnight cultures were initiated with 5-7 mL LB media (Miller, Sigma-Aldrich), 5-7 µL Amp (100 mg/mL), and 5 µL plasmid glycerol stock then grown (37 °C, 225 rpm, overnight). Expression cultures were started with 1000 mL 2xYT media (Sigma-Aldrich), 1000 µL ampicillin stock solution (100 mg/mL), and 5 mL overnight culture. The expression cultures were grown (RT, 80 rpm, in dark) for 2 days, supplemented with 1000 µL ampicillin stock (100 mg/mL), and grown (RT, stationary, in dark) for 2 additional days. Pdx Q88G expression cultures were grown (RT, 80 rpm, in dark) for 1 day then shaking was stopped (RT, stationary, in dark, 6 days). Cell pellets were harvested by centrifugation (6 krpm, 4 °C) and stored at -80°C.

Purification

Cell pellets were resuspended in buffer (20 mM KPi, 5 mM DTT, pH 7.5). Cells were lysed by sonication (70% amplitude, 10-20 cycles 5 s. on, 10 s. off), and soluble protein was separated by ultracentrifugation (12 krpm, 50 minutes, 4 °C). The Pdx Q88G expression pellet was resuspended in 20 mM KPi, 5 mM DTT, pH 7.5, 1x BugBuster (MilliporeSigma, diluted from 10x reagent) and incubated (30 minutes, RT) prior to sonication and ultracentrifugation as previously.

The supernatant was decanted and purified by anion exchange chromatography (DEAE Resin, Bio-Rad). On an equilibrated column, the lysate supernatant was loaded and run. The column was washed with Pdx buffer (20 mM KPi, 5 mM DTT, pH 7.5). Fractions were eluted by increasing KCl concentration (50, 100, 150, 200, 250, 300, 400, 600 mM) and collected.

Fractions containing Pdx (deduced by a brown color) were combined and concentrated (3000-10000 MWCO, 3 krpm, 4 °C).

Concentration was calculated with the following expression (Equation S1).

$$[Pdx\ (mM)] = \frac{A_{455}}{(5.9\ mM^{-1}cm^{-1})(l)} \qquad \text{Equation S1}$$

From the absorbance at 455 nm (A_{455}) and the cuvette path length (l), the concentration was calculated for oxidized Pdx.[1] Sample purity was determined by the ratio of the absorbance at 412 nm (Pdx-specific) to the absorbance at 280 nm (general to proteins).

UV-Vis and Circular Dichroism

Protein concentration and purity was quantified by electronic absorbance (UV-Vis) spectroscopy (Cary 500 Scan UV-Vis-NIR or Agilent 8453 UV-Vis spectrophotometer).

Circular dichroism spectra were collected for WT Pdx in buffer and under mild denaturing conditions (Urea Gradient, *vide infra*). Spectra were collected with a CD spectrometer (Aviv Biomedical Model 430 CD Spectrometer) at 190-260 nm. Spectra were truncated where the dynode current exceeded the 850 V threshold (Figure S11).

Urea Gradient

Samples of 150 μL were prepared of WT Pdx in Pdx sample buffer (50 mM KPi, 100 mM KCl, 10% glycerol, pH 7.5) containing 0, 1, 2, or 4 M urea. WT Pdx stock solution was diluted with buffer containing urea to 145 μL then incubated for 30 minutes at room temperature. 5 μL of 60 mM sodium dithionite in sample buffer was added (final concentration of 2 mM, 10-fold excess relative to Pdx). Samples were transferred to Norell EPR tubes (S-4-EPR-250S) and flash-frozen in liquid nitrogen. Final Pdx WT concentrations were 200 μM, and final urea concentrations were 0, 1, 2, and 4 M.

Mutants

Pdx mutants were buffer exchanged into Pdx buffer (50 mM KPi, 100 mM KCl, 10% glycerol, pH 7.5) and concentrated. Protein concentrations were quantified via UV-Vis with Equation S1. Samples of 145 μL were prepared for each protein variant and reduced with 5 μL 60 mM sodium dithionite (2 mM final concentration) in Pdx sample buffer. Final concentrations were WT Pdx at 200 μM, Pdx G41R at 200 μM, and Pdx Q88G at 79 μM. Samples were transferred to Norrell EPR tubes (S-4-EPR-250S) and flash-frozen.

Deuteration

WT Pdx samples were incubated in buffer prepared in deuterium oxide (D_2O) for varying durations (1 min, 6 min, 30 min, 1 hour, 6 hours, 24 hours, and 48 hours). WT Pdx stock (2 mM) was diluted (10-15 uL) in deuterated buffer (50 mM KPi, 1 M KCl, 10% d8-glycerol (CIL, 99%), pD 7.5, 5 mM BME in D_2O (Thermo Scientific, 99.8%)) and incubated on ice (shorter times) or 4 °C (longer times) for the necessary duration. A control was prepared with the WT Pdx stock diluted in nondeuterated buffer (50 mM KPi, 1 M KCl, 10% glycerol, pH 7.5, 5 mM BME in H_2O) and incubation for 1 minute. Reduction was performed with 5 uL sodium dithionite (60 mM in appropriate buffer), and samples were transferred to Norrell EPR tubes (S-4-EPR-250S) and flash frozen in liquid nitrogen. Samples were 150 μL (except for the 1 hour sample, which was 100 μL) with 200 μM WT Pdx. The Pdx in H_2O buffer control and the Pdx in D_2O buffer (t=48 h.) samples were analyzed by three-pulse ESEEM and HYSCORE at 15 K.

Electron Paramagnetic Resonance (EPR)

X-band continuous-wave (CW) EPR (Bruker EMX) spectra were collected for each sample. For X-band pulse EPR spectroscopy, a Bruker ElexSys E580 pulse EPR spectrometer was used, equipped with an MD4 dielectric ENDOR resonator. Temperature control was achieved using a ColdEdge closed-loop cryogen-free cryostat. Two-pulse echo-detected field-swept spectra and two-pulse (Hahn echo) decays were collected using a $\pi/2$–τ–π–echo pulse sequence. Field-swept spectra were used to determine the perpendicular (≈ 3584 G = 358.4 mT) and parallel (≈ 3436 G = 343.6 mT) field positions used to collect the two-pulse decays ($\frac{\pi}{2}$ = 8 ns). The spectra were collected at temperatures of 10, 15, and 20 K.

Data Processing, Simulation, and Fitting

CW-EPR spectra were simulated with EasySpin's least-squares fitting ('esfit') using the 'pepper' (solid state) core function.[2] Two-pulse decays were fit using a MATLAB script. Two-pulse decays were fit to the conventional phase-memory relaxation expression (Equation S2).

$$I = I_0 + k_m e^{-\frac{2\tau}{T_m}} \qquad \text{Equation S2}$$

The fit exponential was subtracted from the data followed by apodization with a positive Hamming window ('ham+') and zero-filling (8-fold filling), and a fast Fourier transform (FFT) was taken (two-pulse ESEEM). Three-pulse ESEEM and HYSCORE spectra were simulated with the 'saffron' (solid-state pulse EPR) core function.

II. Methodology for Calculations

A. Hyperfine coupling & molecular motion considerations for T_m^{-1}

Previous literature set the precedent that T_m anisotropy (via field or orientation dependence) was dominated by molecular motion. This was demonstrated by Du *et al.* in frozen solutions of copper (2 mM CuDTC and 1 mM CuTTP in 2:1 toluene:chloroform) and vanadyl (2 mM VOTTP-COOH in 9:1 toluene:THF) complexes.[3] Experimental data was correlated to calculations of $dB_{res}/d\theta$, which indicates the change in the resonant magnetic field position (of the EPR transition) with the angle between the molecular z-axis and the magnetic field. The magnetic field and molecular frame of reference are related through the experimental *g*-values. Resonant magnetic fields and *g*-values are related by Equation S3.

$$\mathrm{B}_{\mathrm{res}} = \frac{h\nu_{MW}}{\beta g} \qquad \text{Equation S3}$$

For axial systems, the *g*-value is easily related to the angle (θ) formed by the external magnetic field with the molecular z-axis (Equation S4).

$$\mathrm{g} = \sqrt{\mathrm{g}_{\perp}^2 \sin^2\theta + \mathrm{g}_{\parallel}^2 \cos^2\theta} \qquad \text{Equation S4}$$

The angle formed with the molecular z-axis is conventionally used. In this way, Equations S3 and S4 can be combined (Equation S5).

$$\mathrm{B}_{\mathrm{res}} = \frac{h\nu_{MW}}{\beta\sqrt{\mathrm{g}_{\perp}^2 \sin^2\theta + \mathrm{g}_{\parallel}^2 \cos^2\theta}} \qquad \text{Equation S5}$$

This expression can then be differentiated with respect to θ (Equation S6).

$$\frac{d\mathrm{B}_{\mathrm{res}}}{d\theta} = \frac{h\nu_{MW}\left(g_{\parallel}^2 - g_{\perp}^2\right) sin\theta cos\theta}{\beta\left(\mathrm{g}_{\perp}^2 \sin^2\theta + \mathrm{g}_{\parallel}^2 \cos^2\theta\right)^{\frac{3}{2}}} \qquad \text{Equation S6}$$

The plot of $dB_{res}/d\theta$ versus θ yields a concave down parabola with the maximum just offset above 45° (π/4 rad).

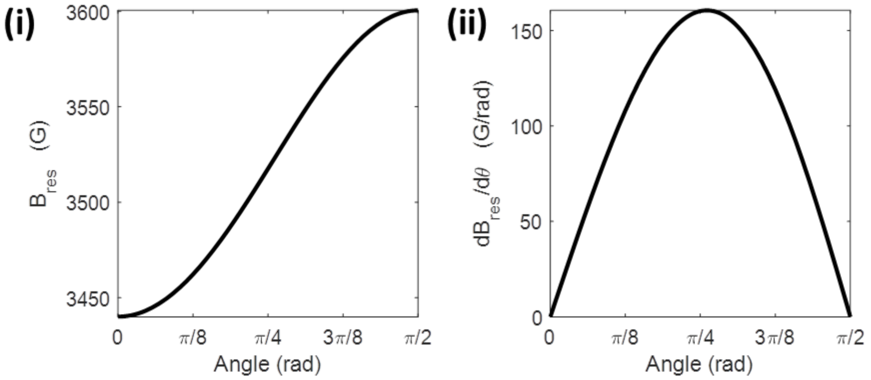

Figure S1. Plots of (i) B_{res} vs θ and (ii) $dB_{res}/d\theta$ versus θ. Here, the *g*-values used are $g_{\perp} = 1.93$ and $g_{//} = 2.02$, and the microwave frequency is set to 9.725 GHz.

The resemblance of experimental T_m^{-1} versus B_0 data to the latter plot (Figure S1.ii) was used as an indication that molecular motion, especially the sensitivity of motion relative to the molecular z-axis, dominated T_m anisotropy in CuDTC, CuTTP, and VOTTP-COOH.[3]

Conversely, the convention for the *g*-value can be flipped via trigonometric identities ($cos\theta = sin\,(\frac{\pi}{2} - \theta)$) to define the angle against the perpendicular plane made by the molecular x- and y-axes. In this way, a new angle ($\theta' = \frac{\pi}{2} - \theta$) can be defined. The analogous form of Equation S4 can be written (Equation S7).

$$g = \sqrt{g_{\parallel}^2 \sin^2\theta' + g_{\perp}^2 \cos^2\theta'} \qquad \text{Equation S7}$$

Subsequently, Equations S5 and S6 can be similarly converted to Equations S8 and S9, respectively.

$$B_{res} = \frac{h\nu_{MW}}{\beta\sqrt{g_{\parallel}^2 \sin^2\theta' + g_{\perp}^2 \cos^2\theta'}} \qquad \text{Equation S8}$$

$$\frac{dB_{res}}{d\theta'} = \frac{h\nu_{MW}\left(g_{\perp}^2 - g_{\parallel}^2\right) sin\theta cos\theta}{\beta\left(g_{\parallel}^2 \sin^2\theta' + g_{\perp}^2 \cos^2\theta'\right)^{\frac{3}{2}}} \qquad \text{Equation S9}$$

In this convention, the plot of $dB_{res}/d\theta'$ versus θ' yields a concave up parabola with the minimum just offset below 45° (π/4 rad).

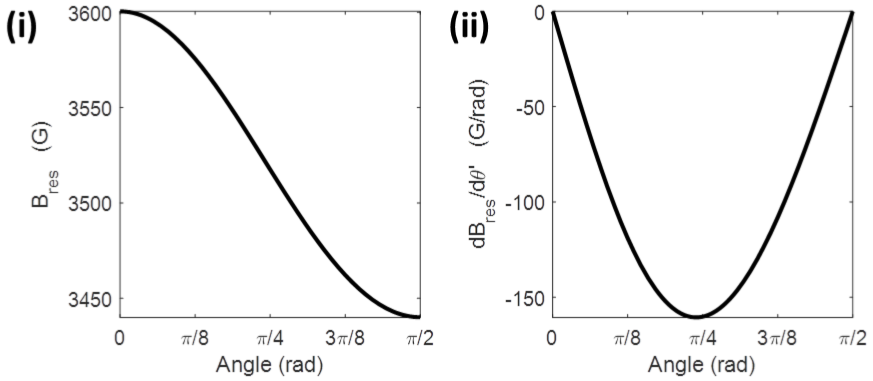

Figure S2. Plots of (i) B_{res} vs θ' and (ii) $dB_{res}/d\theta'$ versus θ'. Same as in Figure S1, the *g*-values used are $g_{\perp} = 1.93$ and $g_{/\!/} = 2.02$, and the microwave frequency is set to 9.725 GHz.

Nonetheless, at low temperatures, hyperfine contributions are expected to dominate.[4] In this way, the T_m anisotropy is expected to be governed by the angular component of the dipolar coupling rather than molecular motion. Dependence on a different trigonometric function (the angular dependence of the dipolar coupling) is expected: $|1 - 3cos^2\theta|$ (Figure S3).

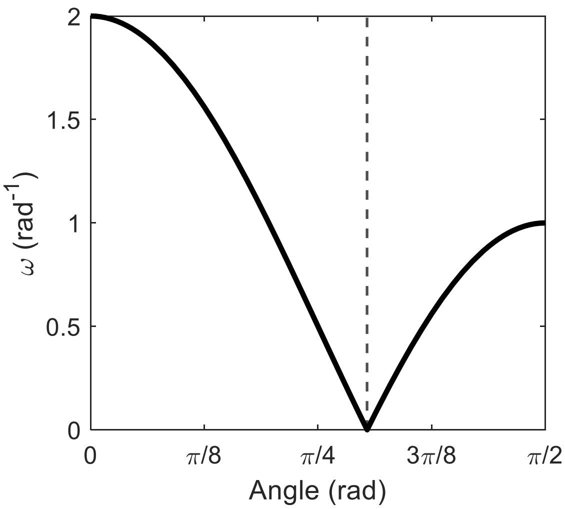

Figure S3. A plot of the angular component ($|1 - 3cos^2\theta|$) of the dipolar coupling. The dashed vertical line denotes the magic angle (0.955 rad = 54.7°).

The minimum from dipolar coupling is defined by the magic angle, where dipolar coupling vanishes to zero ($|1 - 3cos^2\theta| = 0$), and is significantly offset above π/4. Intuitively, for systems where decoherence is dominated by dipolar coupling, the angular position (and, thus, external magnetic field) without dipolar coupling should yield the slowest decoherence rate (and longest decoherence time). Conversion of the magnetic field position (via *g*-values) for the minimum T_m^{-1} value to the angular position can be used to elucidate the dominating relaxation mechanism between hyperfine coupling and molecular motion.

In frozen samples, the paramagnetic molecule will be present at all possible orientations and, thus, angles with the external magnetic field. For determining a functional form, the anisotropic component of the dipolar coupling provides a logical starting point (Equation S10).

$$\omega = \frac{\mu_0 \gamma_e \gamma_n \hbar}{4\pi} \times \frac{(1 - 3\cos^2\theta)}{r^3} \qquad \text{Equation S10}$$

Considering the condition of all random molecular orientations, the dipolar coupling can be adjusted to consider all angular orientations via integration over the surface of a sphere. Integration over the surface of a sphere can be simplified from three dimensions to two dimensions due to the presence of axial symmetry. In this way, the angular integral with respect to the two-dimensional polar angle of the anisotropic dipolar coupling multiplied by $\sin\theta$ yields this average over all orientations (Equation S11).

$$\langle\omega\rangle_\theta = \frac{\mu_0 \gamma_e \gamma_n \hbar}{4\pi r^3} \times \int (1 - 3\cos^2\theta)\sin\theta\, d\theta \qquad \text{Equation S11}$$

Indefinite integration in this way yields an expression with a trigonometric dependence on $\sin^2\theta\cos\theta$ (Equation S12).

$$\langle \omega \rangle_\theta = \frac{\mu_0 \gamma_e \gamma_n \hbar}{4\pi r^3} \times (-\sin^2\theta\cos\theta) + C \qquad \text{Equation S12}$$

This functional form can be visualized (Figure S4).

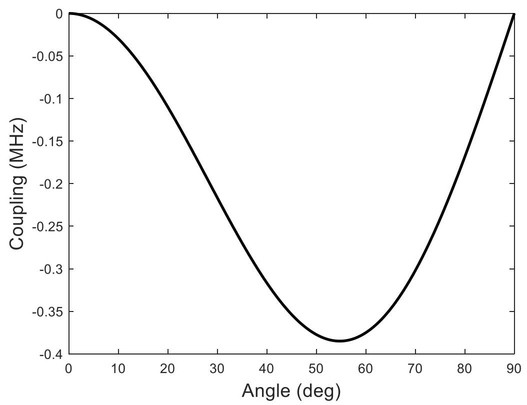

Figure S4. The behavior of the anisotropic dipolar coupling taken over all random orientations. This plot utilizes Equation S12.

Evidently, this matches well with the T_m^{-1} anisotropy observed experimentally taken in the θ' convention (with the angle of the magnetic field taken relative to the xy-plane rather than the z-axis) (Figure S5).

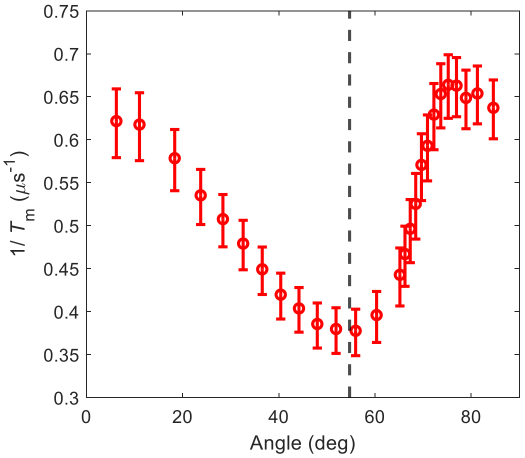

Figure S5. The T_m^{-1} anisotropy of Pdx WT converted to angular coordinates with respect to the xy-plane. Experimental data collected at 15 K and 9.730962 GHz. Conversion to angular coordinates utilized $g_\perp$ = 1.93905 and $g_{/\!/}$ = 2.02438. This vertical dashed line indicates the position of the magic angle (54.7° = 0.955 radians).

Conversely, the functional form derived in Equation S12 can be converted to magnetic field coordinates via the microwave power and *g*-values through Equations S3 and S7 (Figure S6).

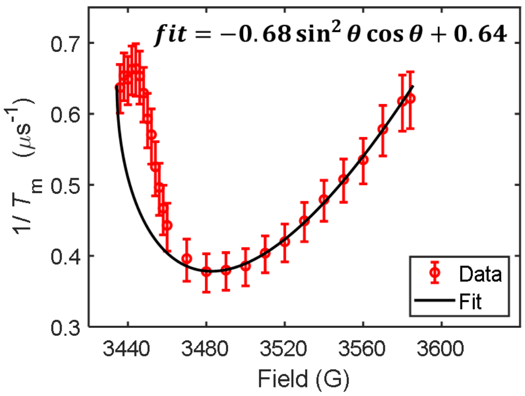

Figure S6. Fitting of the T_m^{-1} field dependence for Pdx WT at 15 K and 9.730962 GHz. Conversion from angular coordinates utilized $g_\perp$= 1.93905 and $g_\parallel$ = 2.02438. The fit used for this dataset is: $fit = -0.68\sin^2\theta\cos\theta + 0.64$.

Near $\frac{\pi}{2}$ radians for the angular plot (Figure S6) or the parallel position for the field plot (Figure S6), the T_m^{-1} anisotropy deviates from the derived expression. Notably, previous T_m anisotropy observed sensitivity to the molecular z-axis orientation near the parallel position.[3,5] This behavior can also potentially be similarly justified. Additionally, WT Pdx's spin environment does incorporate a slight amount of rhombicity and significant ESEEM (from coupling to adjacent nuclei that don't contribute to decoherence) that may deviate the experimental behavior from ideality, as well. Thus, for hyperfine-dominated decoherence in frozen solutions, the decoherence rate appears to be consistent with the following functional form (Equation S13).

$$\frac{1}{T_m} \propto \langle\omega\rangle_\theta = \frac{\mu_0\gamma_e\gamma_n\hbar}{4\pi r^3} \times (-\sin^2\theta\cos\theta) + C \qquad \text{Equation S13}$$

A proportionality is observed between $1/T_m$ and $\langle\omega\rangle_\theta$. Integration of the dipolar component of the hyperfine coupling with respect to the angle (θ) provides the averaged dipolar coupling across all orientations. In a large macromolecular system, like a metalloproteins, the large number and arrangement of protons past the spin-diffusion barrier may be approximated by such averaging over all orientations. In a frozen solution spectrum, the absorptive features of a field-swept spectrum indicate the orientations of molecules on-resonance at a particular magnetic field strength. Furthermore, the observation of this proportionality may indicate collinearity between the components of the g- and A-tensors, as is observed in uniaxially-symmetric systems. In this way, the magic angle is defined relative to the molecule's unique axes, namely relative to the plane of the cluster.

B. Approximation of T_m

In systems with many protons, hyperfine interactions can dominate decoherence through spin-spin relaxation mediated by spin flips.[4] The electron spin of interest undergoes spin flips paired with surrounding nuclear spins. Protons bear a nuclear spin of I = ½ and thus can undergo such spin flips. For systems such as proteins, the network of nuclear spins surrounding the electron spin center gives rise to a hyperfine field, through electron-nucleus dipolar coupling. Spatial and temporal fluctuations within this hyperfine field contribute to the decoherence rate (phase memory rate, T_m^{-1}) of the electron spin.

The hyperfine field's contribution to decoherence rate can be approximated from the concentration of local nuclear spins. Assuming abundant protons and a cubic lattice of nuclear spins around the electron spin, the accompanying decoherence rate reduces to the following algebraic expression (Equation S14).

$$\frac{1}{T_m} = \frac{0.37\mu_0(g_e\beta_e)^{\frac{1}{2}}(g_n\beta_n)^{\frac{3}{2}}[I(I+1)]^{\frac{1}{4}}C_n}{4\pi\hbar} \qquad \text{Equation S14}$$

Here, μ_0 is the permeability of free space, g_e is the electron g-factor, β_e is the Bohr magneton, g_n is the nuclear g-factor, β_n is the nuclear magneton, I is the nuclear spin number, $\hbar$ is the reduced Planck constant, and C_n is the number density of nuclear spins.[6]

The use of a cubic lattice approximation assumes an isotropic system. As noted in the main text, Pdx displays axial CW-EPR spectra. Thus, the anisotropic experimental decoherence times can be averaged to an isotropic value (Equation S15).

$$\frac{1}{T_{m,iso}} = \frac{2}{3}\left(\frac{1}{T_{m,\perp}}\right) + \frac{1}{3}\left(\frac{1}{T_{m,\parallel}}\right) \qquad \text{Equation S15}$$

This calculation is analogous to the averaging of $g_\perp$ and $g_\parallel$ to g_{iso}. With the control data from the urea gradient experiment and Equation S15, the calculated $T_{m,iso}$ is 1.714 µs (Table S1). Similarly, the isotropic T_m values for the 1 M, 2 M, and 4 M urea samples were calculated to 1.338, 1.307, and 1.364 µs, respectively (Table S1). These data indicate, that upon introduction of urea, the decoherence rate increases by 28%, 31%, and 26%, respectively. Notably, assuming a protein partial specific volume of 0.7 - 0.76 cm^3/g for Equation S14, the solvent rate exceeds the protein rate by 15 - 25% (Table S1).

Since biomolecules both contain and reside in microenvironments containing abundant protons, equation S14 can be applied to them. This main text does so for Pdx. Naturally, the cubic lattice approximation is invalid for most molecular systems, but it enables a simplified estimation of T_m^{-1}. At low temperatures and spin-dilute conditions, hyperfine-mediated spin-spin relaxation is expected to dominate decoherence. For molecular and biomolecular systems, two zones of influence and, thus, contributions are expected: intramolecular and intermolecular interactions. The former arise from dipolar coupling between the electron spin and nuclei within the protein (both on the backbone and on the amino acid residues/side chains). The latter is from the same type of coupling to (mostly) aqueous solvent nuclei but also to other solute species, such as buffering reagents. Both of these regions yield additive components to the overall decoherence rate (Equation S16).

$$\frac{1}{T_{m,total}} = \frac{1}{T_{m,solvent}} + \frac{1}{T_{m,protein}} \qquad \text{Equation S16}$$

The protein and solvent contributions were approximated from protein structural information and bulk solution properties. PDB protein crystal structures were used to determine the concentration of hydrogens and other nonzero spin nuclei within the protein. Similarly, the protein buffer provided the concentration of nuclei within the solvent. This method of approximation assumes similar protein conformations between the solid crystal and frozen solution states. Yet, in this way, it may offer information regarding flexibility of protein structure in solution. From only mixed contributions from the protein and solvent, a solvent contribution factor (ϵ_s) can be introduced that indicates the T_m contribution from the solvent (ϵ_s) and from the protein ($1 - \epsilon_s$) (Equation S17).

$$\frac{1}{T_{m,total}} = \epsilon_s \frac{1}{T_{m,s}} + (1 - \epsilon_s) \frac{1}{T_{m,p}} \qquad \text{Equation S17}$$

Equation S16 can be similarly applied to also account for proton and deuteron contributions to the decoherence rates from both the solvent and protein hydrogens (Equation 18) upon deuteration of the microenvironment.

$$\frac{1}{T_{m,total}} = \epsilon_s \left[\frac{\alpha_H}{T_{m,s,H}} + \frac{\alpha_D}{T_{m,s,D}}\right] + (1 - \epsilon_s) \left[\frac{\beta_H}{T_{m,p,H}} + \frac{\beta_D}{T_{m,p,D}}\right]$$

Equation S18

$$= \epsilon_s \left[\frac{\alpha_H}{T_{m,s,H}} + \frac{1 - \alpha_H}{T_{m,s,D}}\right] + (1 - \epsilon_s) \left[\frac{\beta_H}{T_{m,p,H}} + \frac{1 - \beta_H}{T_{m,p,D}}\right]$$

As previously, the proton and deuteron contributions to the solvent and protein decoherence rates can be related ($\alpha_D = 1 - \alpha_H$ and $\beta_D = 1 - \beta_H$) since they provide the upper and lower bounds on the decoherence rate. Due to solvent mixing during the exchange process and prior to freeze-quenching, it can be reasonably assumed that the proton (α_H) and deuteron (α_D) contributions to the solvent term are constant from sample preparation. From this, the proton and deuteron contributions to the protein term vary with incubation time. An intermediate regime may be present from solvent molecules hydrogen bonding to the protein that are more difficult to exchange, but that is omitted here for simplicity. The presence of two canonical positions provides the capability for enhanced spatial resolution to the exchange process in structurally complex proteins. In that way, different orientations permit varying contributions from different nuclei and, thus, additional resolution.

Table S1. Comparison of experimental and approximated T_m values.

	Phase memory time (T_m, μs)
**Experimental value (0 M Urea control, 10 K, perpendicular)**	1.763
**Experimental value (0 M Urea control, 10 K, parallel)**	1.617
➔ **Resultant $T_{m,iso}$**	1.714
**$T_{m,iso}$ (1 M Urea)**	1.338
**$T_{m,iso}$ (2 M Urea)**	1.307
**$T_{m,iso}$ (4 M Urea)**	1.364
Approximations:	
**Protein only contribution (all nuclei; uniform sphere)**	3.814
**Protein only contribution (hydrogens only; uniform sphere)**	3.834
**Protein only (v = 0.76 cm^3/g; hydrogens only)**	2.796
**Protein only (v = 0.70 cm^3/g; hydrogens only)**	2.575
**Solvent only contribution (all nuclei)**	2.245

Methodology for approximated table values

The "protein only contribution (all nuclei; uniform sphere)" is calculated from a Pdx crystal structure (PDB: 1XLQ with removal of C73S mutation). Within 13 Å (radius) of the Fe_2S_2 center, the number of nuclei is counted (360 hydrogens, 64 nitrogens, 224 carbons, 67 oxygens, and 10 sulfurs). These correspond to isotopes with nonzero nuclear spins: ^1H, ^2H, ^{14}N, ^{15}N, ^{13}C, ^{17}O, and ^{33}S. Each of these values is divided by the volume of a sphere with radius 13 Å to yield a concentration. Each concentration (C_n) is used (with the appropriate I and g_n values) to calculate a decoherence rate per isotope via Equation S1. These rates added to yield a total rate and, thus, $T_{m,total}$ (= 3.814 μs) (Equation S19).

$$\frac{1}{T_{m,total}} = \sum_{i=\,isotopes} \frac{1}{T_{m,i}} \qquad \text{Equation S19}$$

Naturally, this methodology assumes a uniform distribution of nuclei within the sphere, which is not physically accurate. Thus, it underestimates the concentration (C_n) and overestimates T_m.

The "protein only contribution (hydrogen only; uniform sphere)" calculation follows the same methodology but only accounts for protons (^1H). The difference is 0.5% of the *all nuclei* value above due to the proton's large gyromagnetic ratio and concentration.

For the "protein only (v = 0.76 cm^3/g; hydrogens only)" and "protein only (v = 0.70 cm^3/g; hydrogens only)" cases, a methodology was used to determine the protein volume (rather than a sphere with 13 Å radius). Literature indicates that the average range of partial specific volumes for proteins is 0.70 to 0.76 cm^3/g. Using these values along with the molecular mass of Pdx (11.55 kDa), the volume of a single Pdx protein was calculated. The number of hydrogens was counted (782) within a single Pdx molecule (PDB: 1XLQ). With these two values, the density of hydrogens within a single Pdx molecule is determined and used as C_n in Equation S14.

This assumes that the hydrogen concentration within the whole protein is uniform. This assumption is a better than the *uniform sphere* cases above because it doesn't encroach into the solvent accessible regions around Pdx. T_m values were calculated for both the upper and lower limits of average protein partial specific volume, and both of these values yield T_m's above that for the solvent only case. Thus, increasing solvent accessibility should decrease decoherence time. This result was supported experimentally through the urea gradient experiment (*vide infra*).

Finally, the "solvent only contribution (all nuclei)" is calculated from the concentrations of all nonzero nuclear spin isotopes within the Pdx buffer. The concentration of each buffer component containing these elements is multiplied by the number of each $I \neq 0$ element in the molecular formula to give the elemental concentrations for each individual component. Then, the individual nuclear concentrations are summed to a total solution nuclear concentration (per isotope) that is used as C_n (with the appropriate I and g_n values) in Equation S14. The concentration is also multiplied by the relative abundance of each isotope. Finally, the individual decoherence rates from each isotope are added and converted to a T_m value. By far, the major contributor is protons from water ([H_2O (l)] = 55.5 M & [H_{H2O}] = 111 M). For simplicity, this assumes that volumes (of the buffering components) are additive.

III. Hyperfine-dominated Decoherence in Pdx WT

Note: CW-EPR of Pdx WT and simulation parameters shown alongside mutants spectra in Section VI.

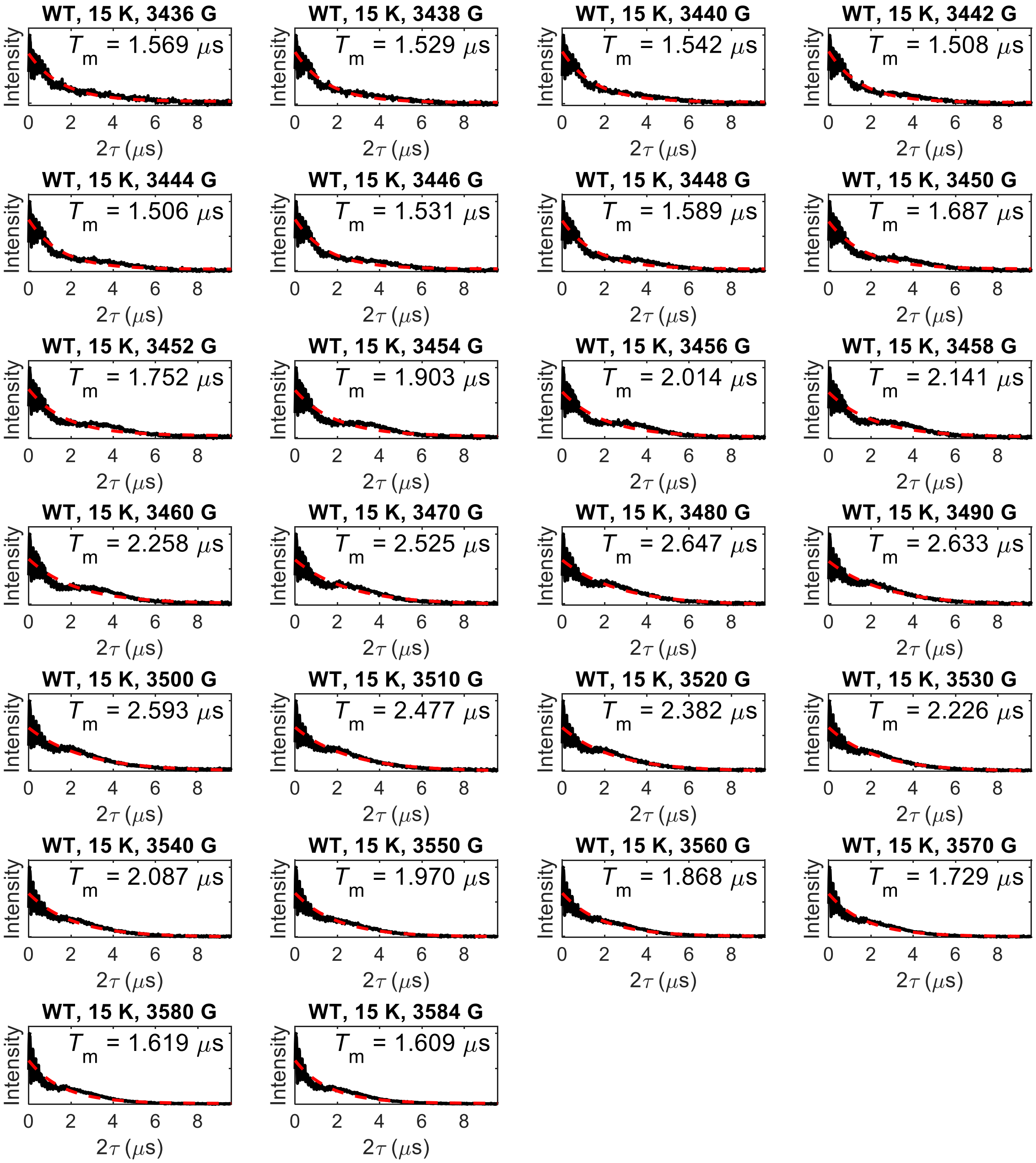

Figure S7. Two-pulse decays for Pdx WT at 15 K and 9.73 GHz at fields from 3436 to 3584 G (1 G = 0.1 mT). Data are shown as black, solid lines with fits as red, dashed lines. Decoherence times are inlaid.

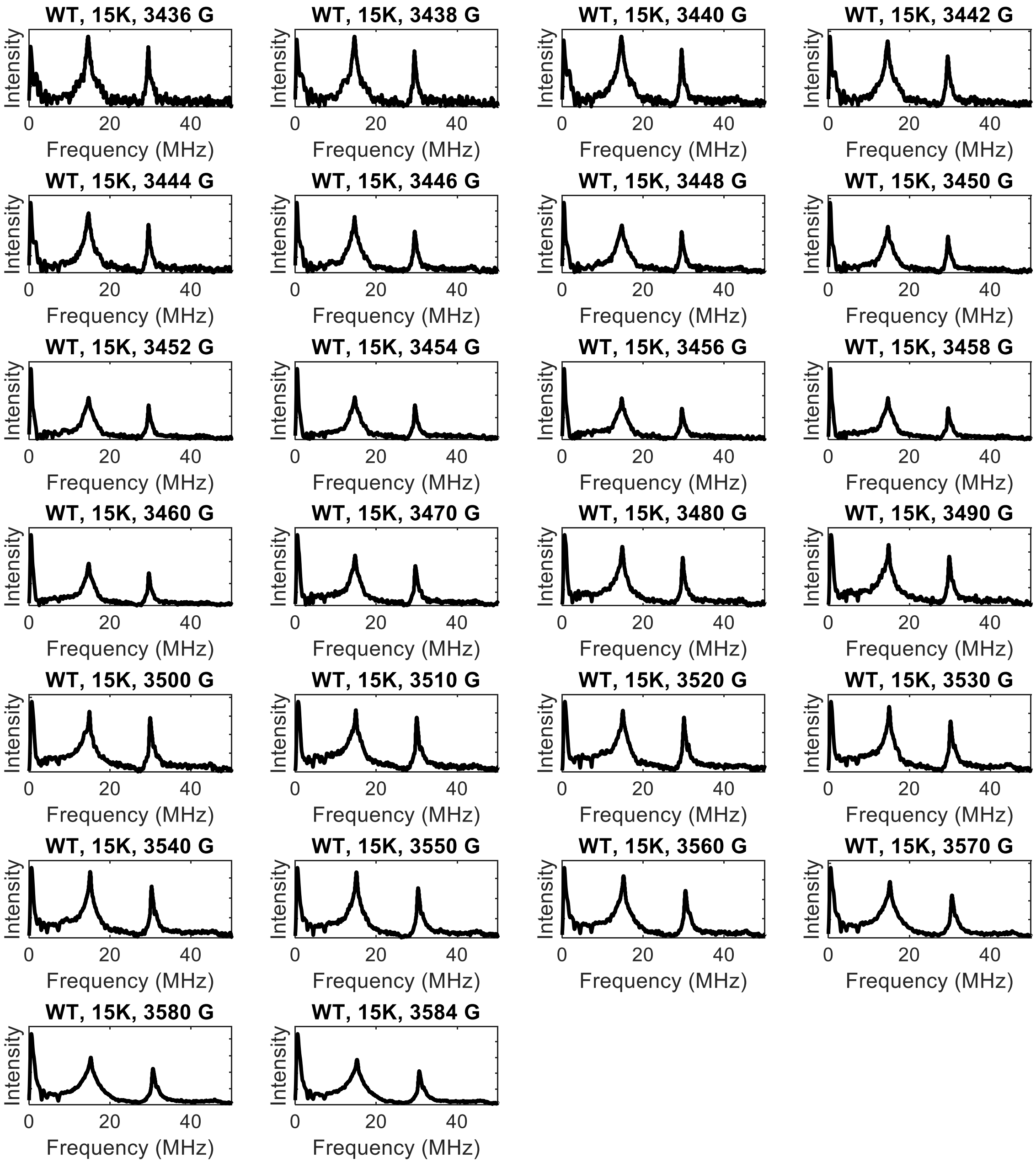

Figure S8. Fast Fourier transforms (FFTs) for Pdx WT at 15 K and 9.73 GHz at fields from 3436 to 3584 G. Data are shown as black, solid lines following subtraction of fit from two-pulse decay traces and subsequent FFT (two-pulse ESEEM). Data (600 points) were apodized with a positive Hamming window (‘ham+’) and zero-filled (4800 zeros).

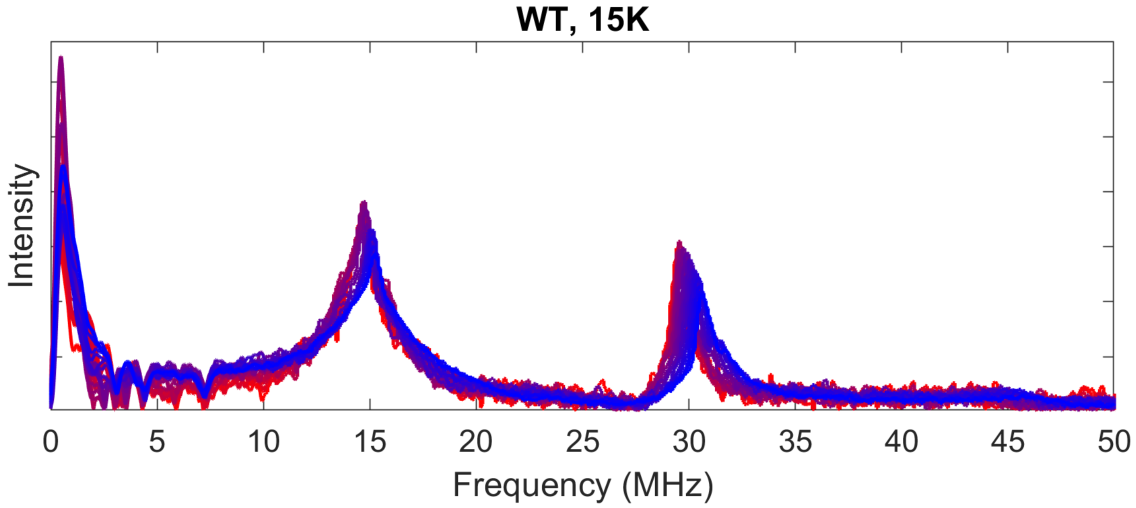

Figure S9. Overlaid FFTs for Pdx WT at 15 K and 9.73 GHz at fields from 3436 to 3584 G. Data are shown as solid lines from 3436 G (red) to 3584 G (blue).

IV. Urea Gradient

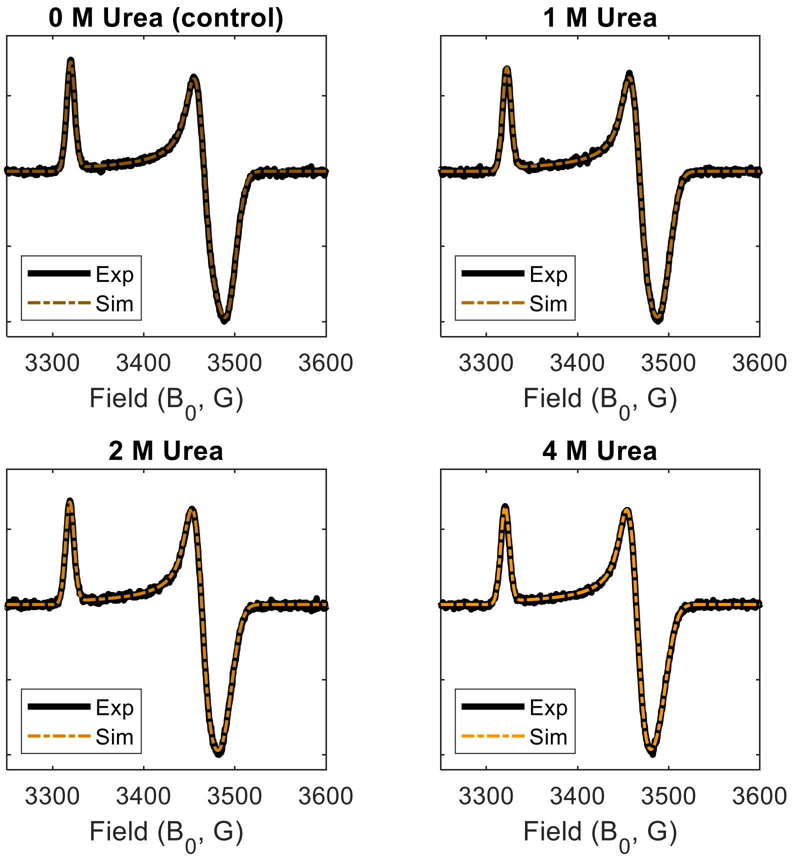

Figure S10. CW-EPR data (black, points) with simulations (red, dashed line) for Pdx WT with buffer containing 0-4 M urea at 77 K. Spectra were collected with 3 scans and 1 G modulation amplitude.

Table S2. CW-EPR simulation parameters for Pdx WT samples with urea gradient with *g* strain[7] at 77 K.

Pdx WT	$g_\perp$ (x, y)	$g_\parallel$ (z)	g-Strain(1)	g-Strain(2)	g-Strain(3)
0 M urea (control)	(1.9215, 1.9362)	2.0214	0.012661	0.011307	0.0065265
1 M urea	(1.9227, 1.9365)	2.0211	0.013320	0.011133	0.0067102
2 M urea	(1.9238, 1.9370)	2.0215	0.014101	0.010991	0.0067218
4 M urea	(1.9243, 1.9369)	2.0212	0.014781	0.010818	0.0068308

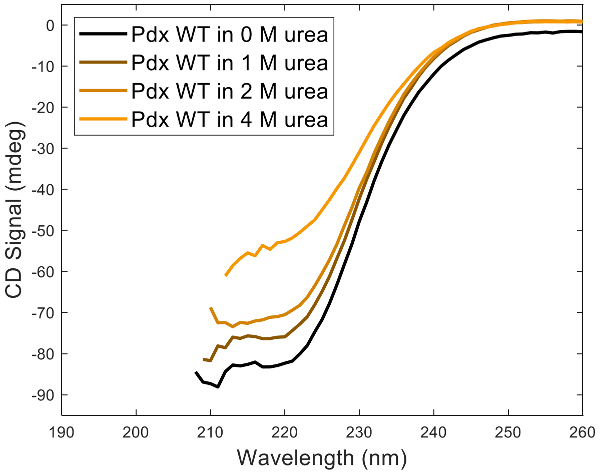

Figure S11. CD spectra of Pdx WT in buffers with increasing concentrations of urea (0 – 4 M) to demonstrate protein unfolding through loss of characteristic secondary structure (mainly α-helix) CD intensity. Plots are truncated at wavelengths where the dynode signal exceeds 850 V.

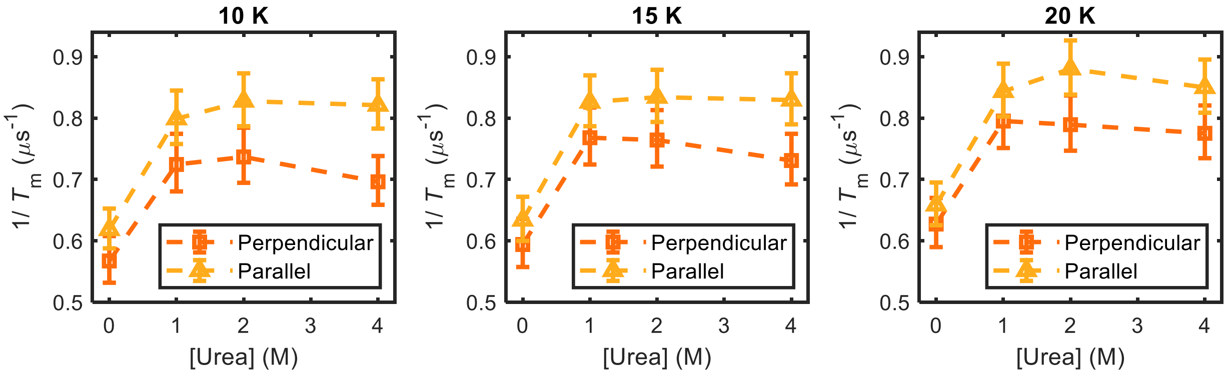

Figure S12. 1/T_m vs [Urea]. Concentration dependence of the decoherence rate (1/T_m, μs^{-1}) on urea concentration (M) at 10, 15, and 20 K with 95% confidence intervals.

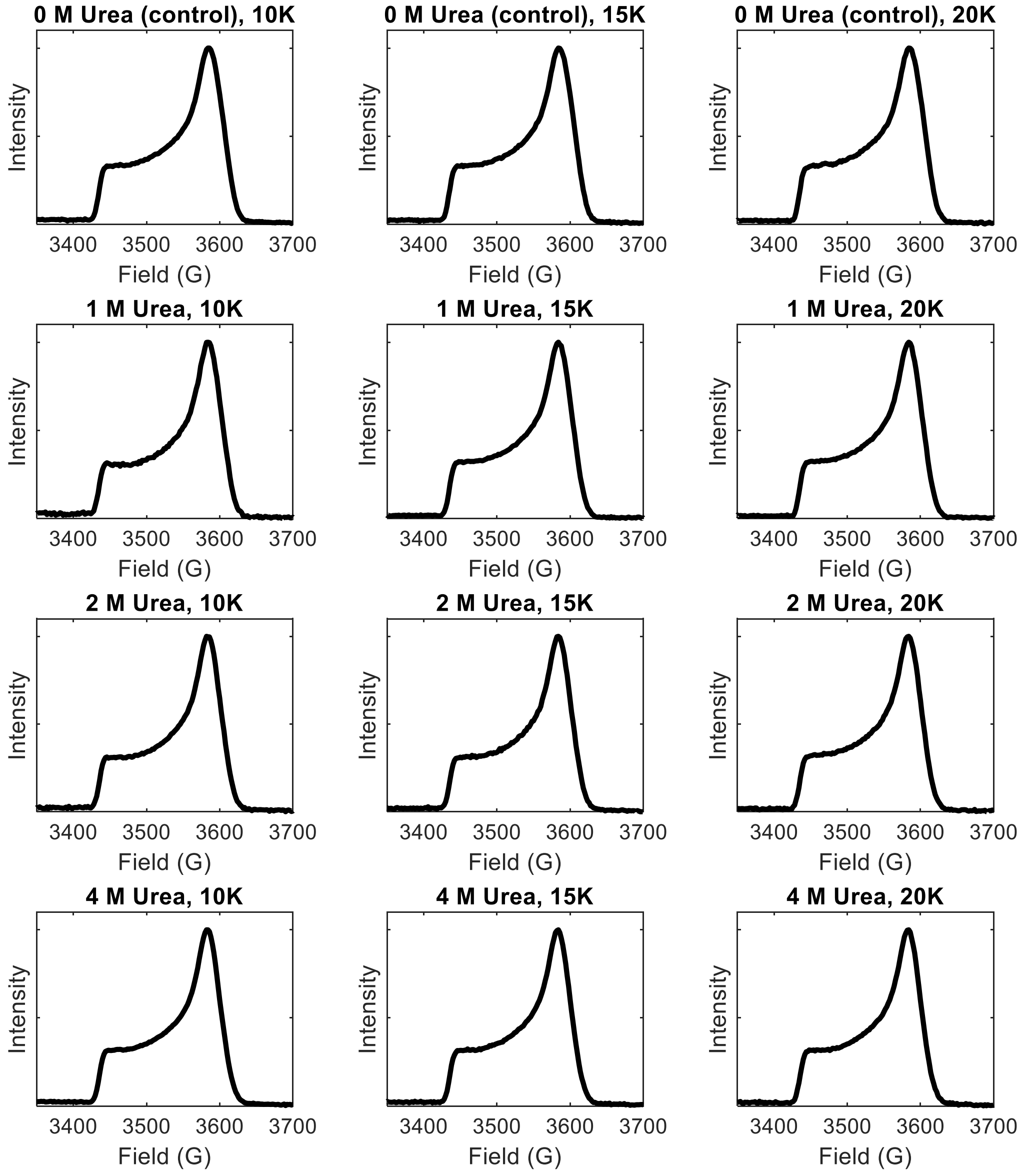

Figure S13. Echo-detected field sweeps of urea gradient Pdx WT samples at 10, 15, and 20 K at 9.73 GHz. Intensity are normalized and in arbitrary units.

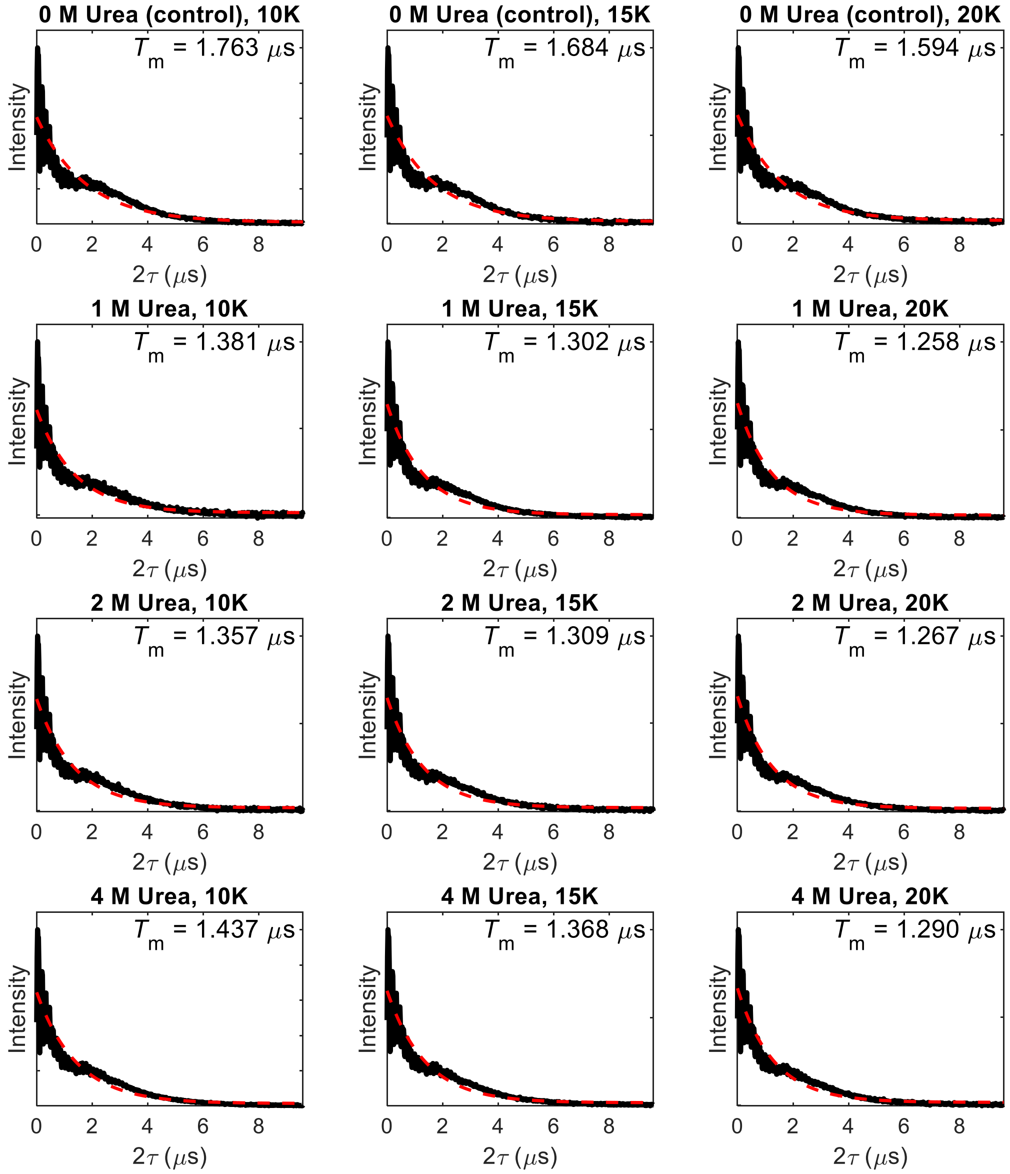

Figure S14. Two-pulse decay data (black, solid line) of urea gradient Pdx WT samples at the perpendicular field position (3584 G) and at 10, 15, and 20 K at 9.73 GHz with accompanying fits (red, dashed line). Decoherence times are inlaid.

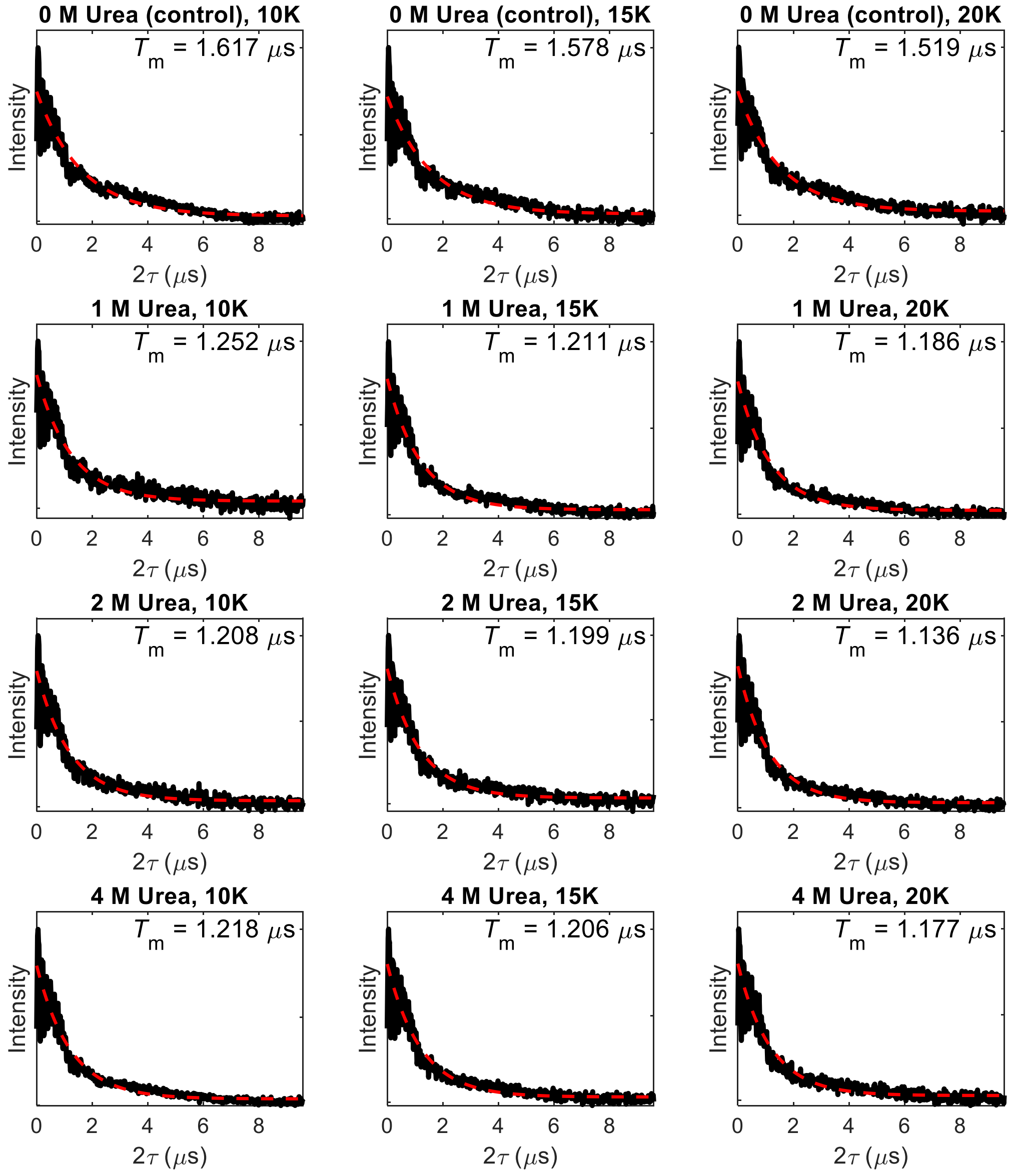

Figure S15. Two-pulse decay data (black, solid line) of urea gradient Pdx WT samples at the parallel field position (3436 G) and at 10, 15, and 20 K at 9.73 GHz with accompanying fits (red, dashed line). Decoherence times are inlaid.

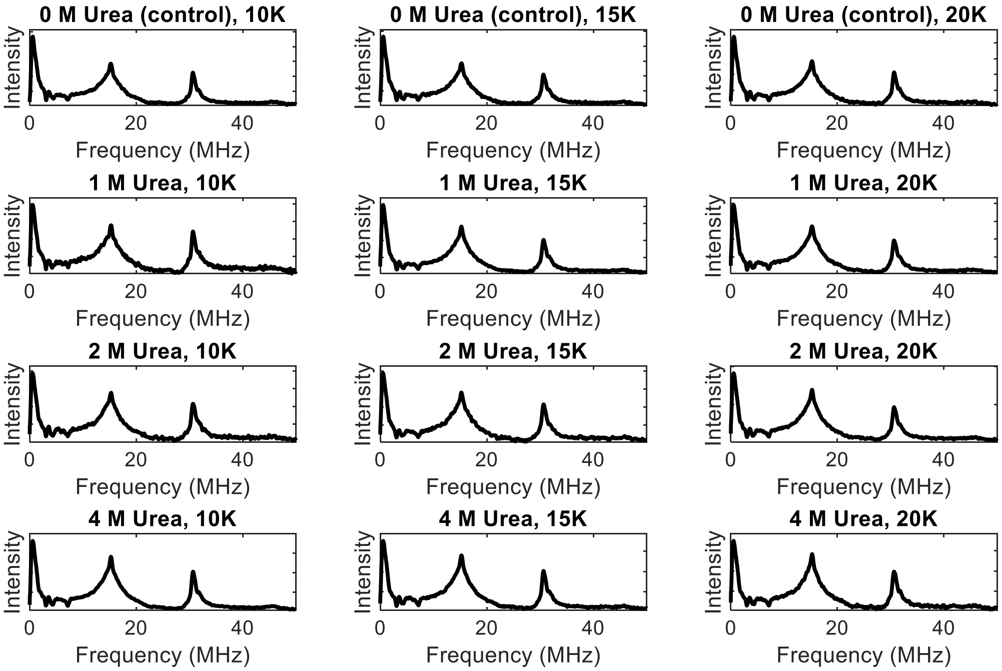

Figure S16. FFTs of fit-subtracted two-pulse decay data (two-pulse ESEEM) at the perpendicular field position (3584 G) at 10, 15, and 20 K. Data (600 points) were apodized with a positive Hamming window ('ham+') and zero-filled (4800 zeros).

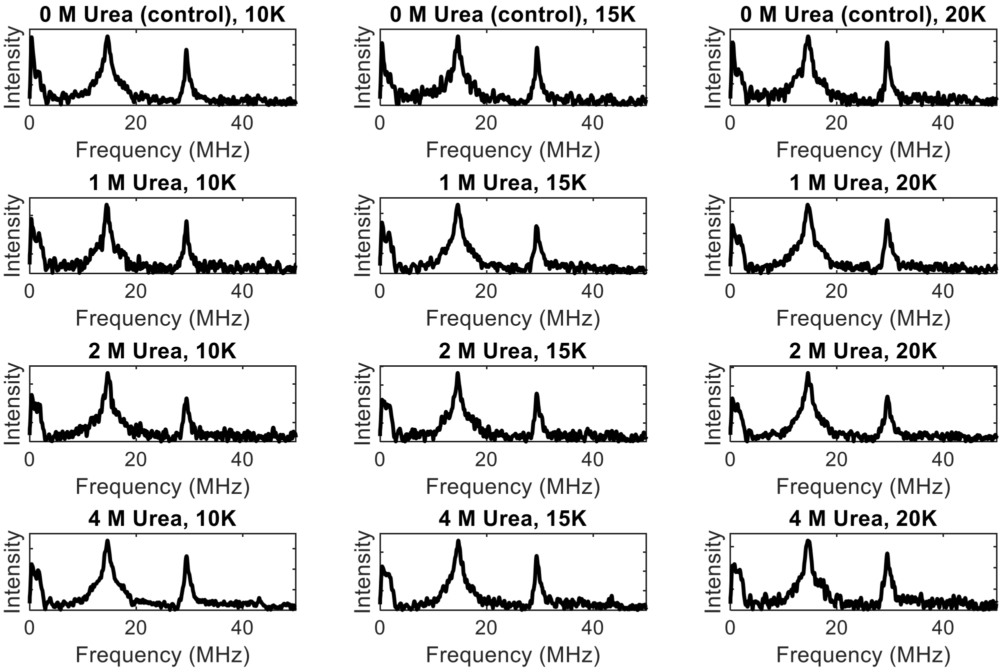

Figure S17. FFTs of fit-subtracted two-pulse decay data (two-pulse ESEEM) at the parallel field position (3436 G) at 10, 15, and 20 K. Data (600 points) were apodized with a positive Hamming window ('ham+') and zero-filled (4800 zeros).

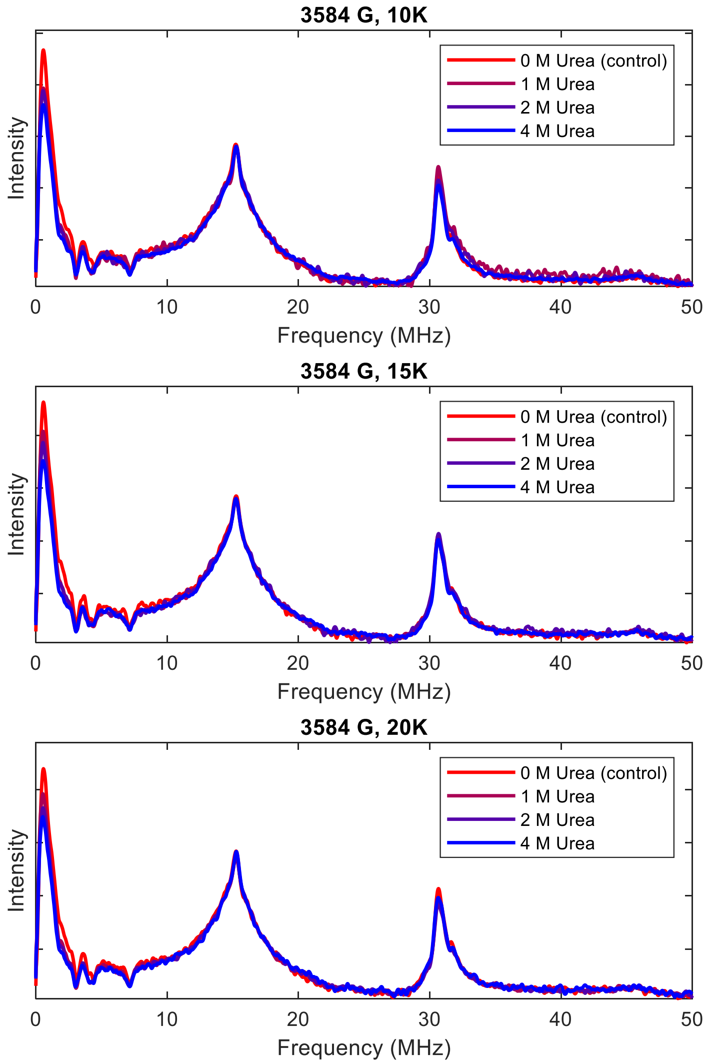

Figure S18. Overlaid FFTs of fit-subtracted two-pulse decay data at perpendicular (3584 G) field position at 10, 15, and 20 K at 9.73 GHz.

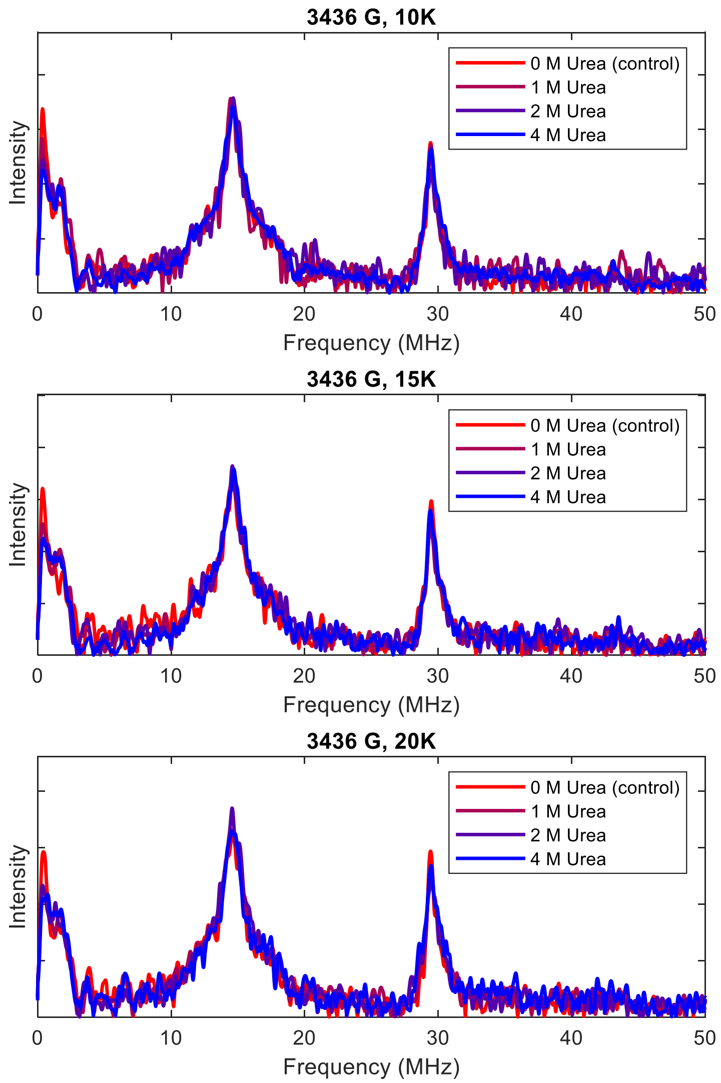

Figure S19. Overlaid FFTs of fit-subtracted two-pulse decay data at parallel (3436 G) field position at 10, 15, and 20 K at 9.73 GHz.

V. *Solvent Deuteration*

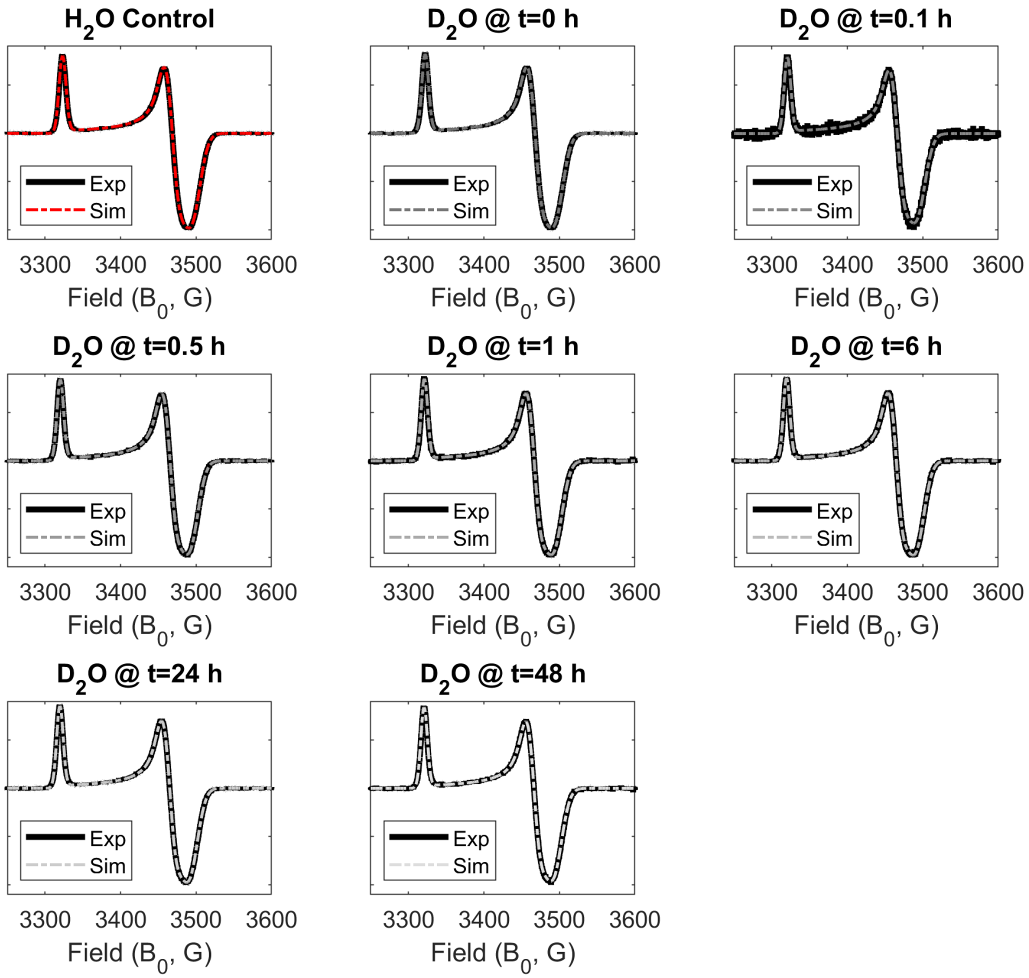

Figure S20. CW-EPR data (black, points) at 77 K with simulations (red, dashed line) for Pdx WT in H_2O (control) and D_2O buffers at varying incubation times (0-48 h). Spectra were collected with 3 scans and 1 G modulation amplitude.

Table S3. CW-EPR simulation parameters for Pdx WT samples in deuterated buffer (variable incubation time) with *g* strain.

Pdx WT	Incubation Time	$g_\perp$ (x,y)	$g_\parallel$ (z)	g-Strain(1)	g-Strain(2)	g-Strain(3)
H_2O	-	(1.9214, 1.9364)	2.0211	0.014211	0.011151	0.0065694
t = 0	1 min	(1.9216, 1.9367)	2.0215	0.014144	0.011103	0.0064354
t = 0.1	6 min	(1.9217, 1.9368)	2.0216	0.014150	0.011067	0.0063343
t = 0.5	30 min	(1.9217, 1.9369)	2.0217	0.014081	0.010963	0.0062524
t = 1	1 h	(1.9217, 1.9369)	2.0217	0.014034	0.010886	0.0062170
t = 6	6 h	(1.9218, 1.9369)	2.0217	0.014033	0.010867	0.0061904
t = 24	24.1 h	(1.9217, 1.9369)	2.0217	0.013994	0.010898	0.0061866
t = 48	48.1 h	(1.9217, 1.9369)	2.0217	0.014014	0.010870	0.0061914

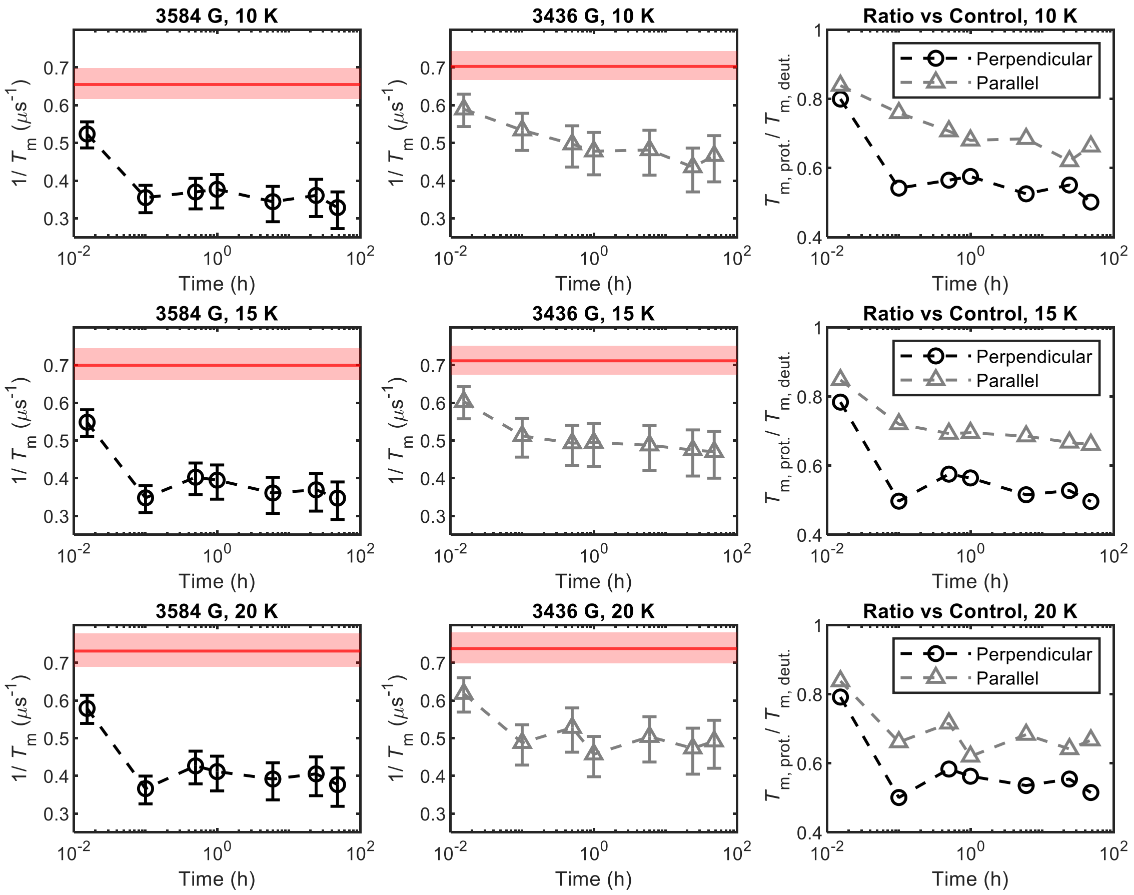

Figure S21. 1/T_m vs incubation time. The dependence of the decoherence rate (1/T_m, μs^{-1}) on incubation time at the perpendicular (3584 G) and parallel (3436 G) field positions at 10, 15, and 20 K. The experimental rates (black circles and gray triangles) are shown relative to the nondeuterated control (red line) with 95% confidence intervals. The rightmost column displays the ratio of deuterated rate versus control (nondeuterated) rate for both field positions and all three temperatures.

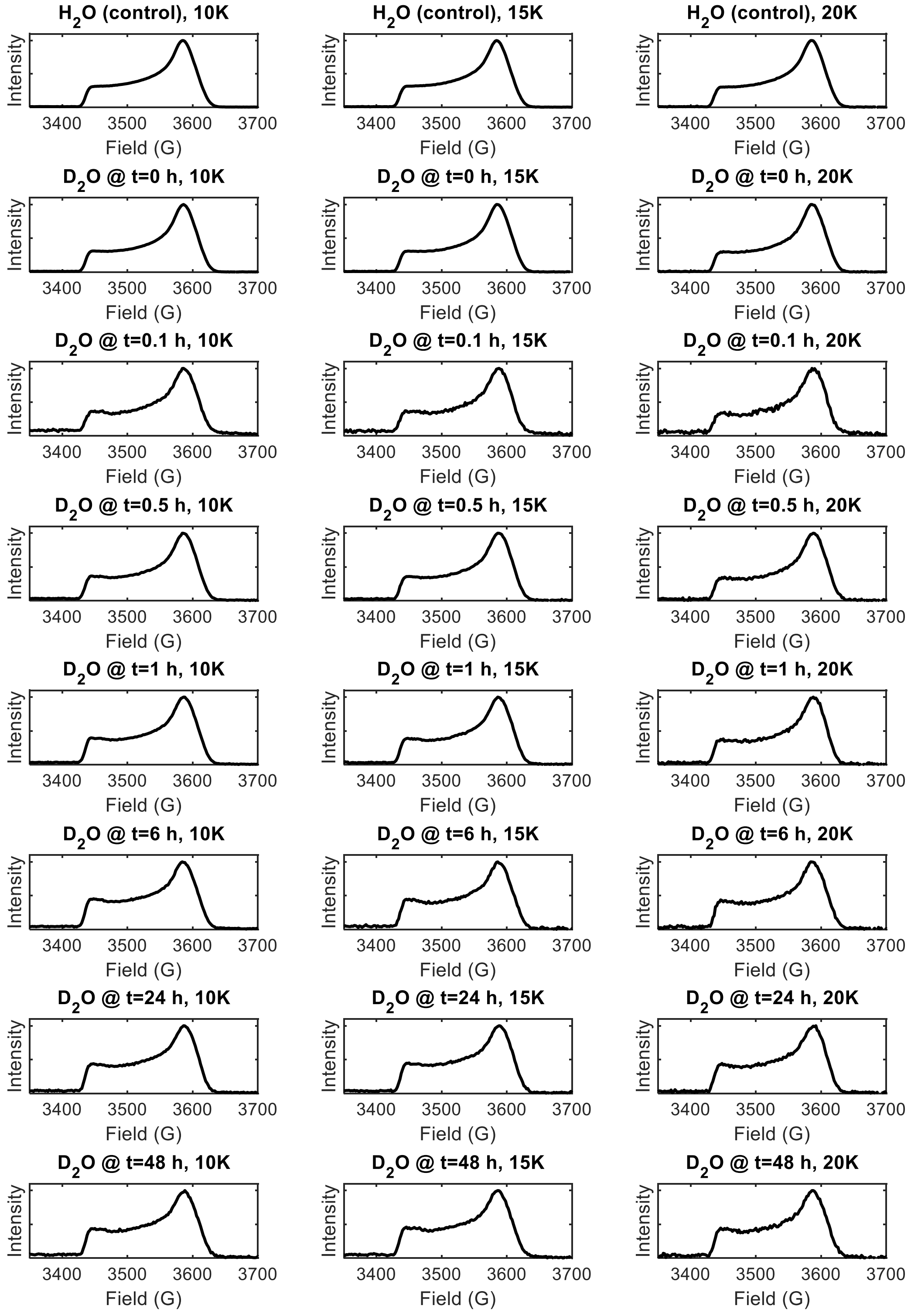

Figure S22. Echo-detected field sweeps of deuterated Pdx WT samples at 10, 15, and 20 K at 9.73 GHz. Intensity are normalized and in arbitrary units.

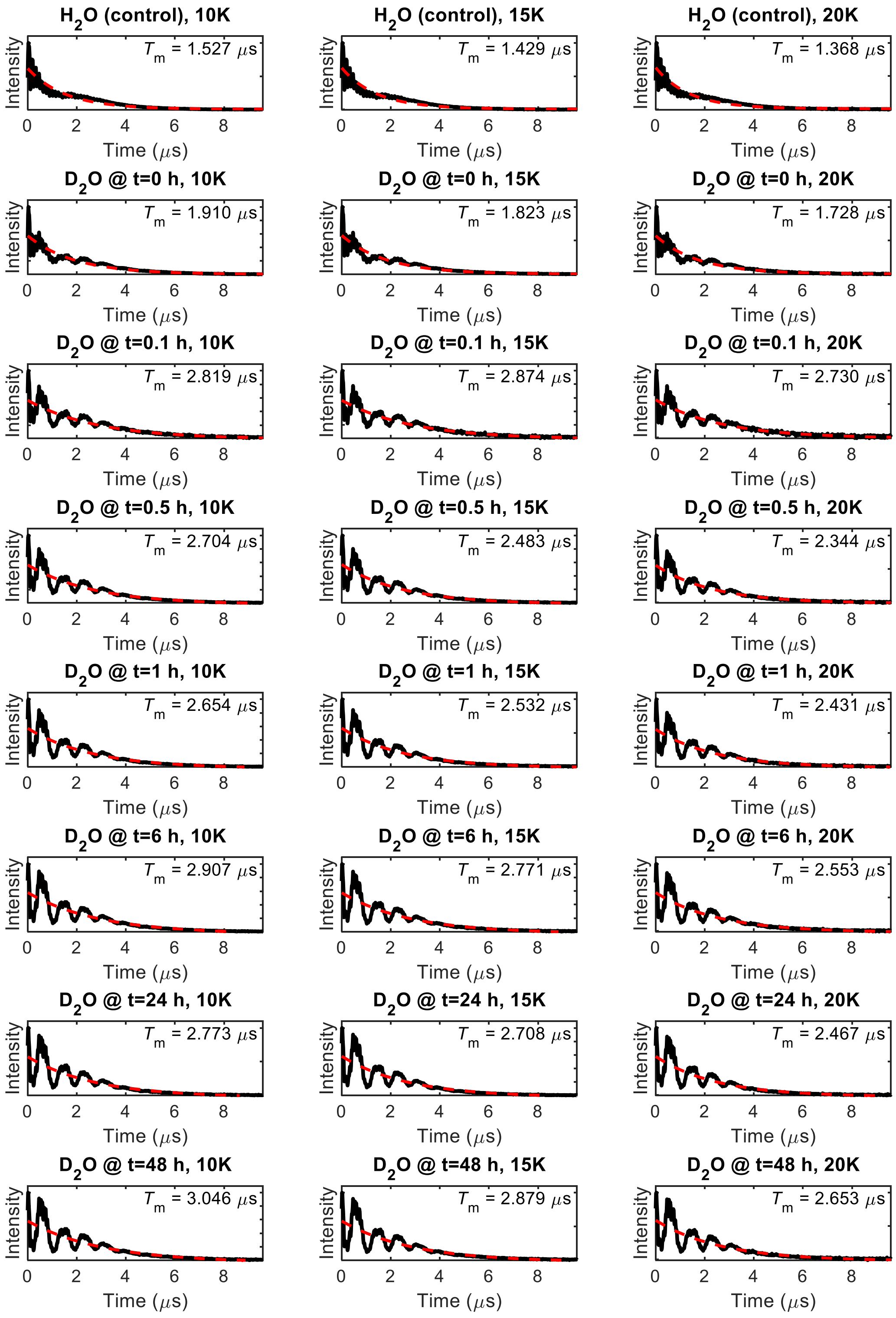

Figure S23. Two-pulse decay data (black, solid line) of deuterated Pdx WT samples at the perpendicular field position (3584 G) and at 10, 15, and 20 K at 9.73 GHz with accompanying fits (red, dashed line). Decoherence times are inlaid.

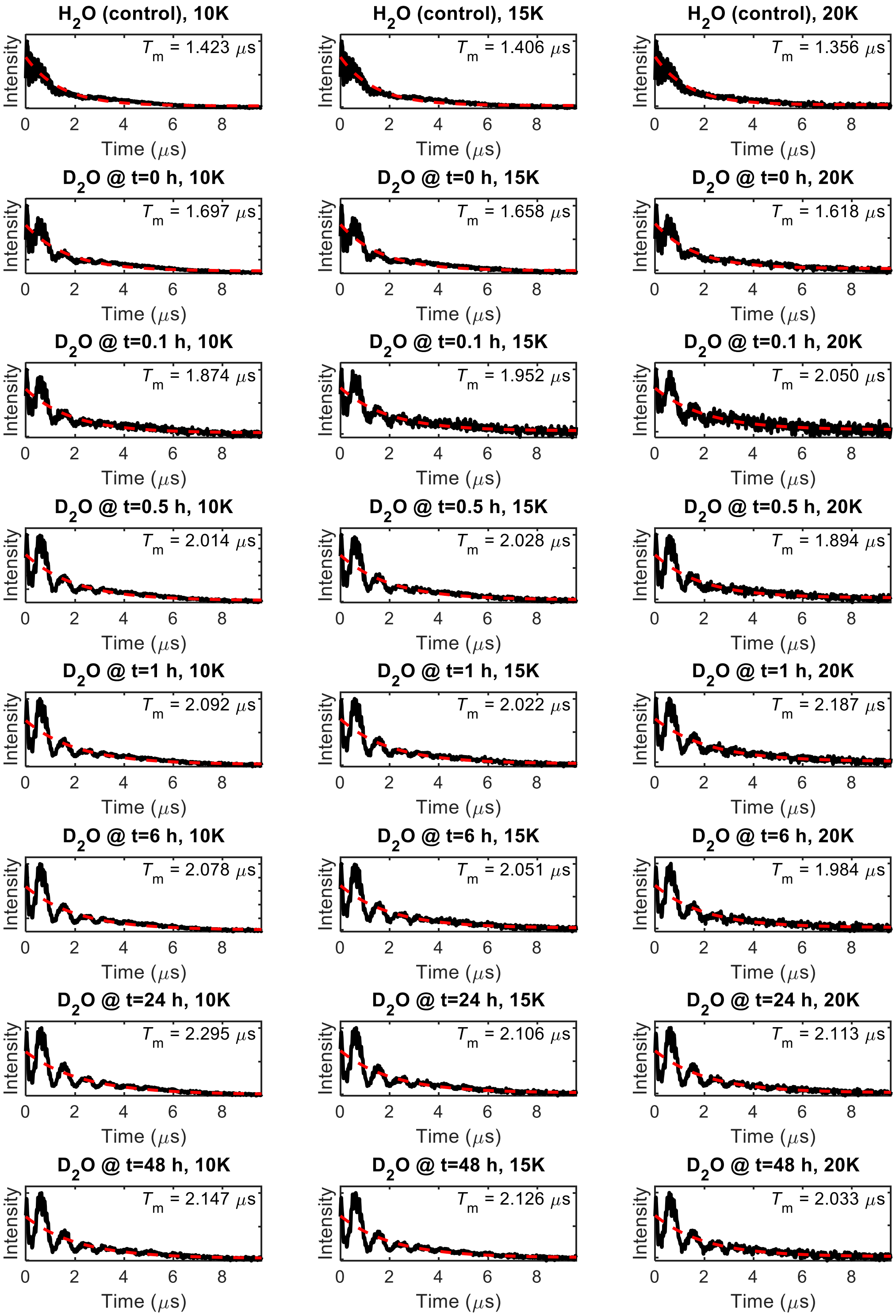

Figure S24. Two-pulse decay data (black, solid line) of deuterated Pdx WT samples at the parallel field position (3436 G) and at 10, 15, and 20 K at 9.73 GHz with accompanying fits (red, dashed line). Decoherence times are inlaid.

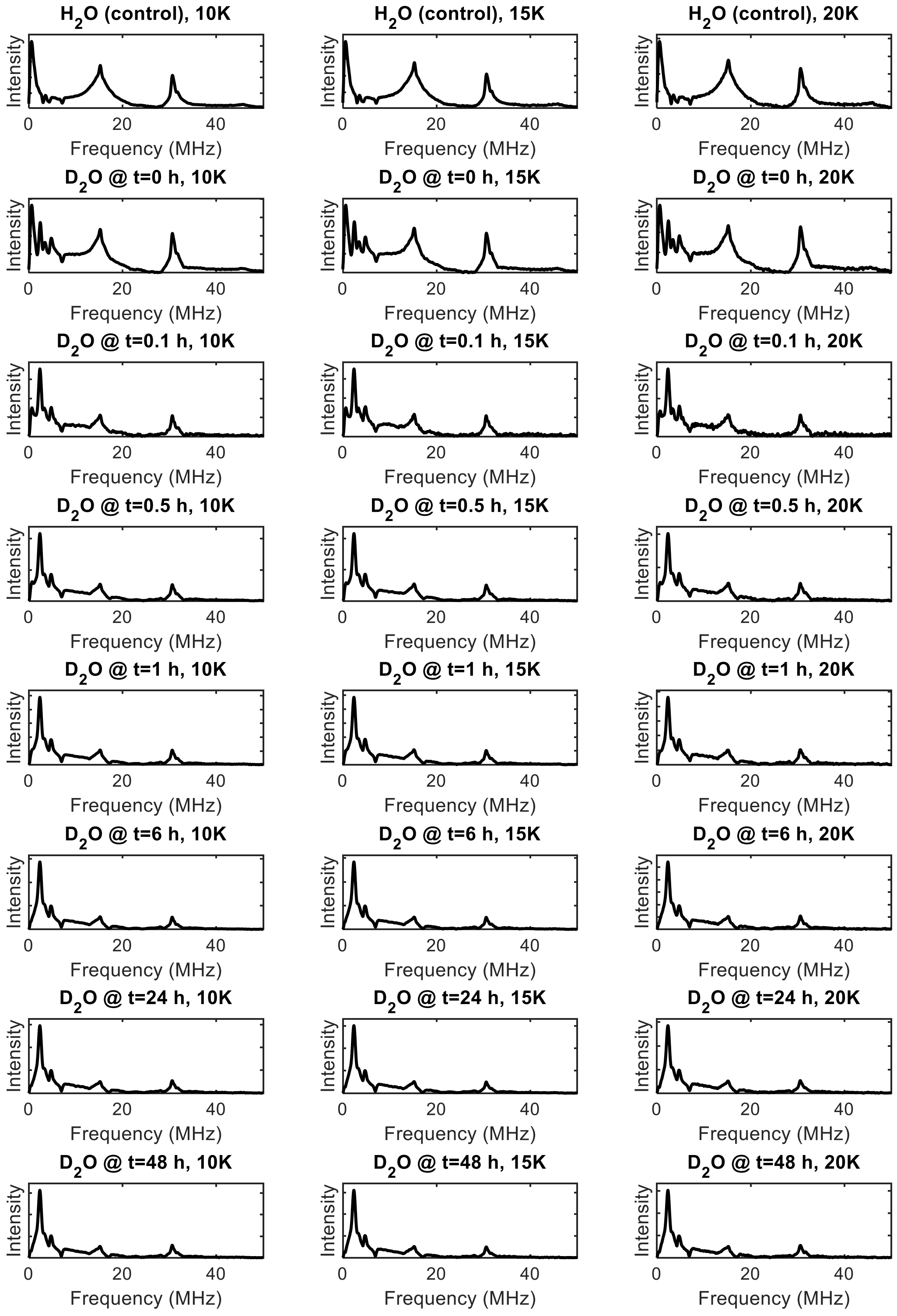

Figure S25. FFTs of fit-subtracted two-pulse decay data (two-pulse ESEEM) at the perpendicular field position (3584 G) at 10, 15, and 20 K at 9.73 GHz. Data (600 points) were apodized with a positive Hamming window (‘ham+’) and zero-filled (4800 zeros).

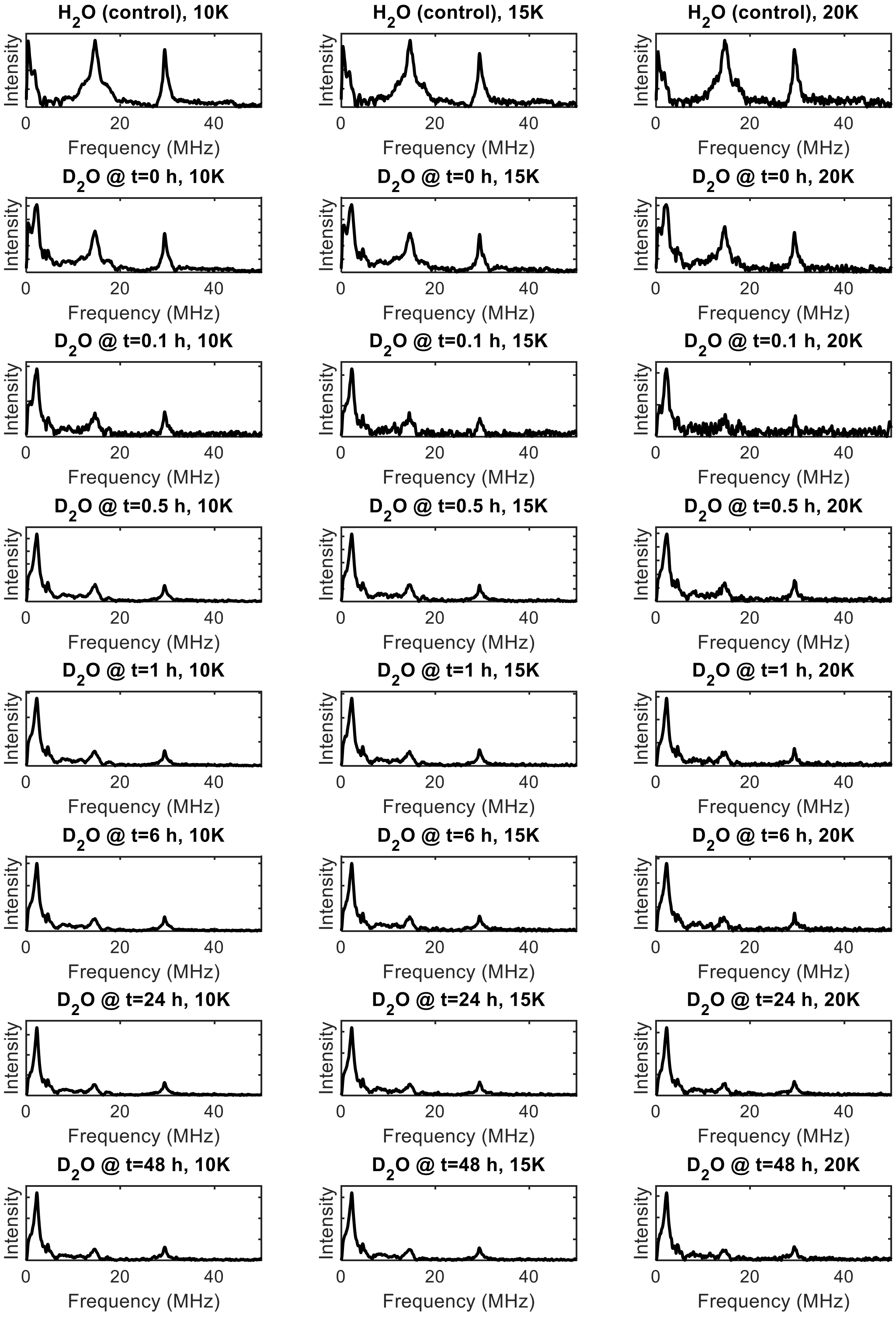

Figure S26. FFTs of fit-subtracted two-pulse decay data (two-pulse ESEEM) at the parallel field position (3436 G) at 10, 15, and 20 K at 9.73 GHz. Data (600 points) were apodized with a positive Hamming window ('ham+') and zero-filled (4800 zeros).

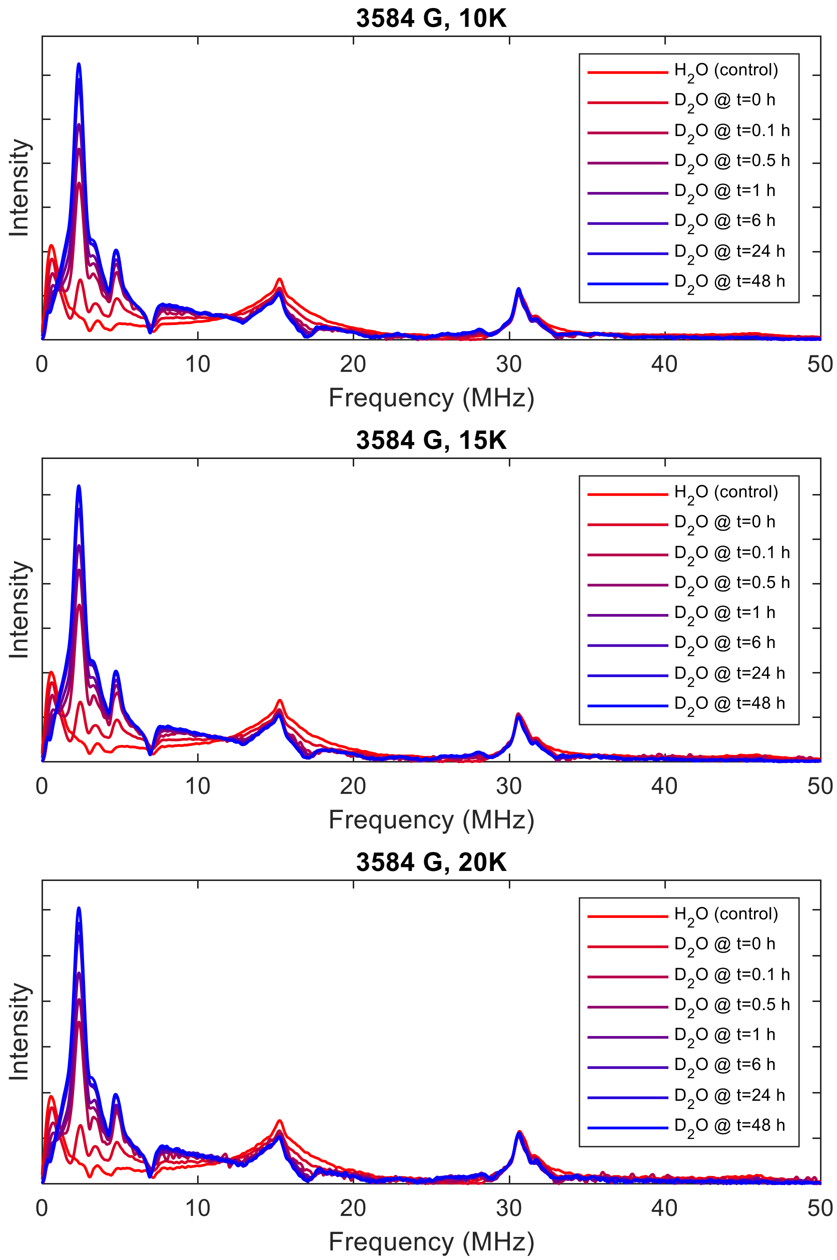

Figure S27. Overlaid FFTs of fit-subtracted two-pulse decay data at perpendicular (3584 G) field position at 10, 15, and 20 K.

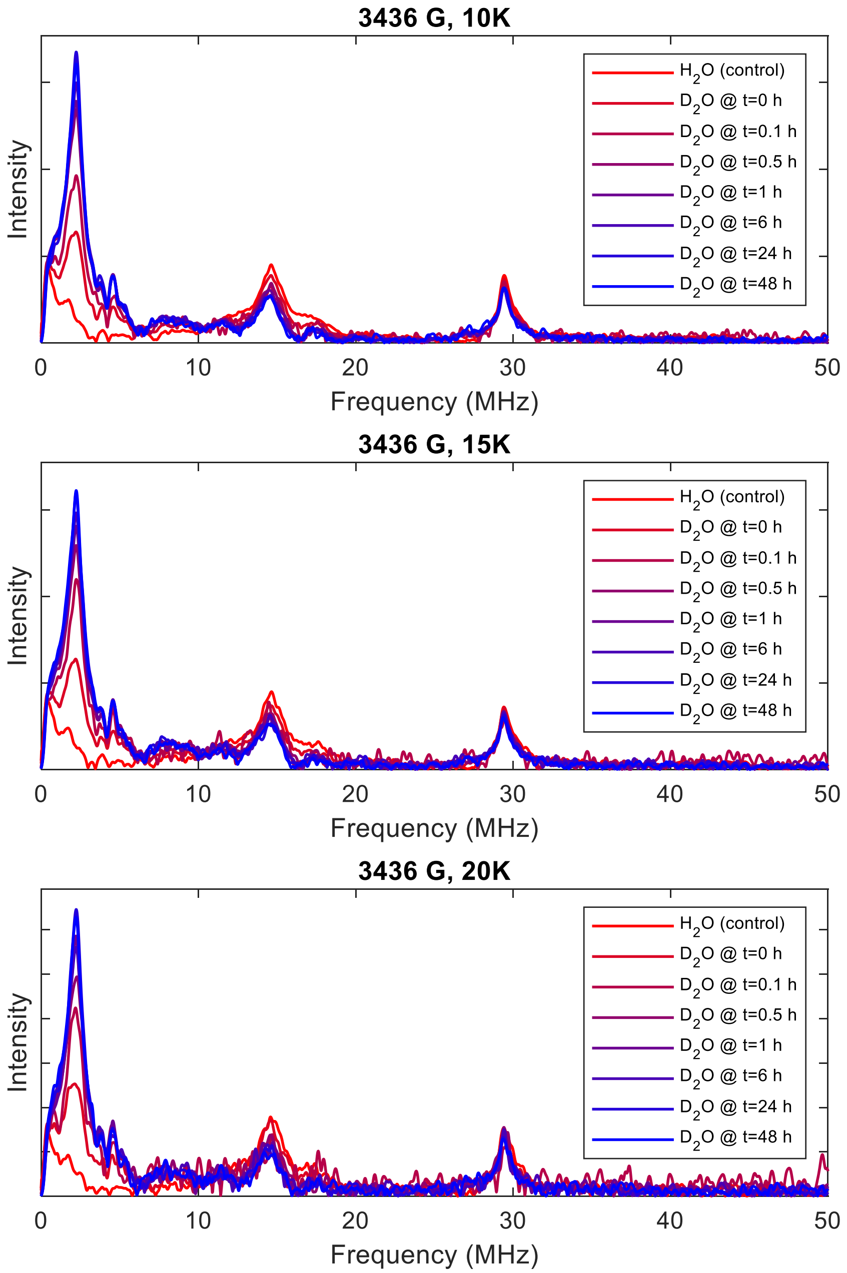

Figure S28. Overlaid FFTs of fit-subtracted two-pulse decay data at parallel (3436 G) field position at 10, 15, and 20 K.

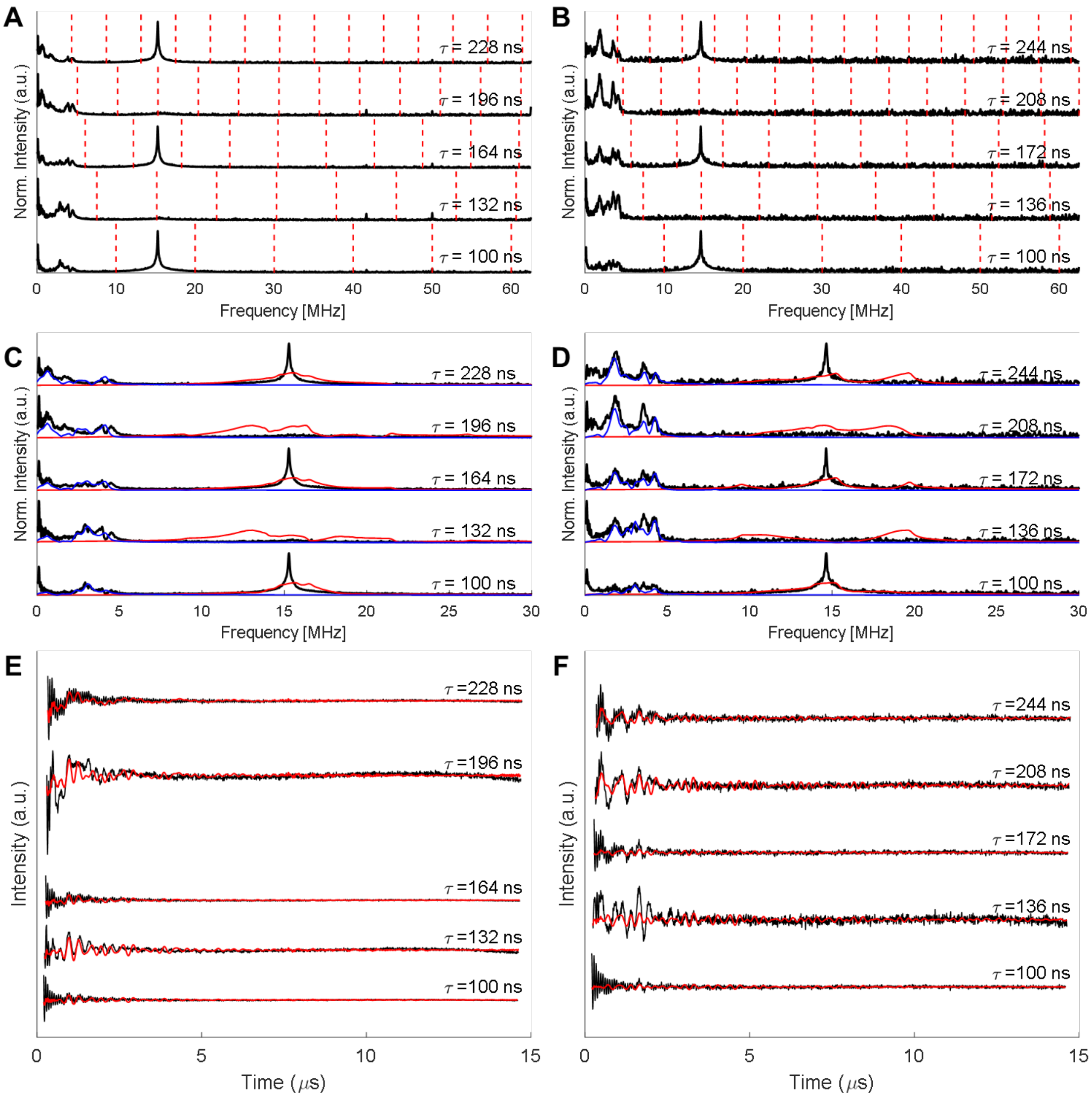

Figure S29. Three-pulse ESEEM data for control at 3584 and 3436 G. (A,B) Frequency domain three-pulse ESEEM spectra for the control sample at (A) 3584 G and (B) 3436 G with blind spots (dashed, red lines) shown. (C,D) Frequency domain data and simulation with ^{1}H (red) and ^{14}N (blue) at (C) 3584 G and (D) 3436 G. (E,F) Time domain spectra and simulations with ^{1}H (red) and ^{14}N (blue) at (E) 3584 G and (F) 3436 G.

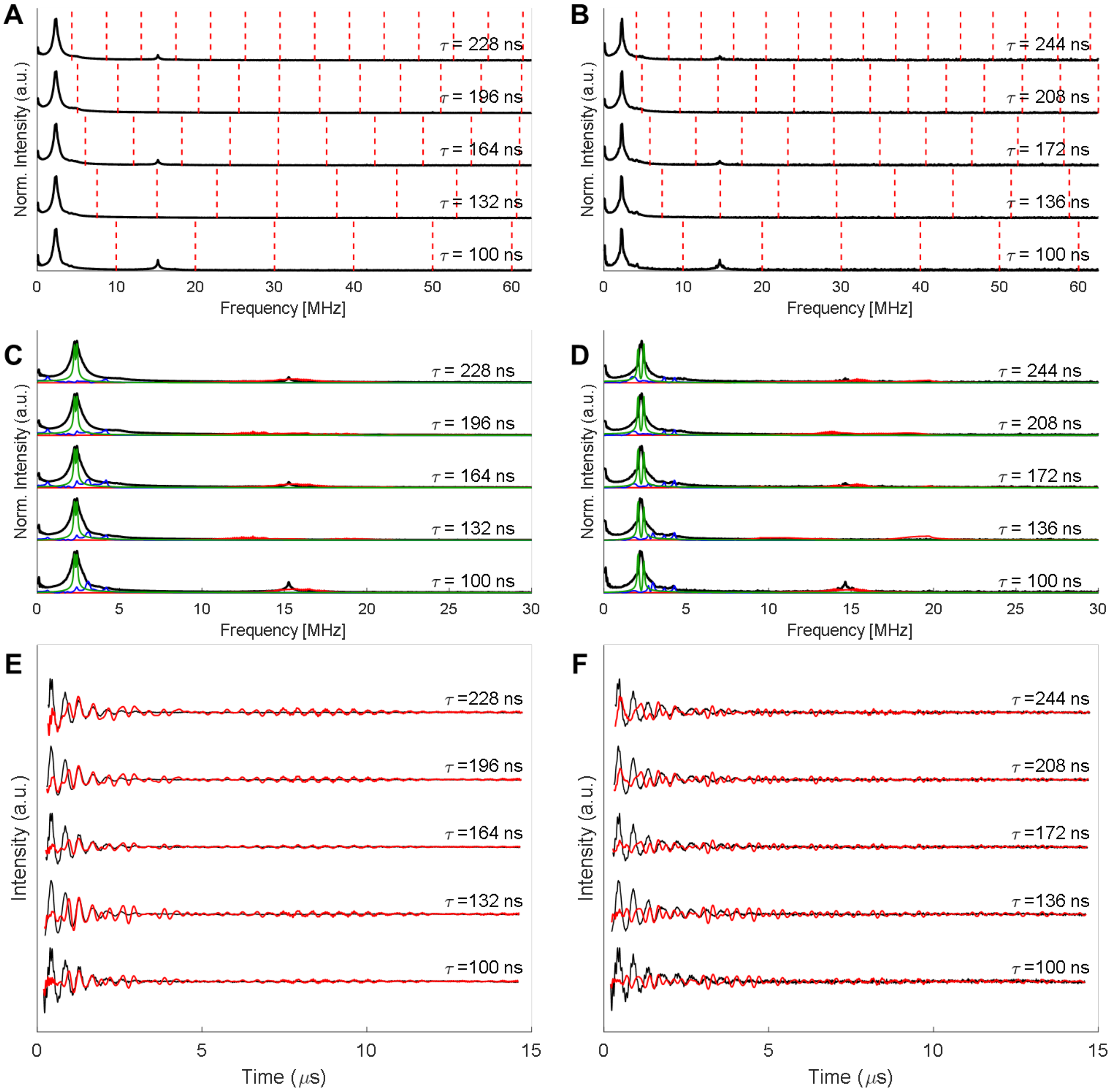

Figure S30. Three-pulse ESEEM for deuterated sample (t=48 h) at 3584 and 3436 G. (A,B) Frequency domain three-pulse ESEEM spectra for the deuterated sample at (A) 3584 G and (B) 3436 G with blind spots (dashed, red lines) shown. (C,D) Frequency domain data and simulation with ^{1}H (red), ^{14}N (blue), and ^{2}H (green) at (C) 3584 G and (D) 3436 G. (E,F) Time domain spectra and simulations with ^{1}H, ^{14}N, and ^{2}H at (E) 3584 G and (F) 3436 G.

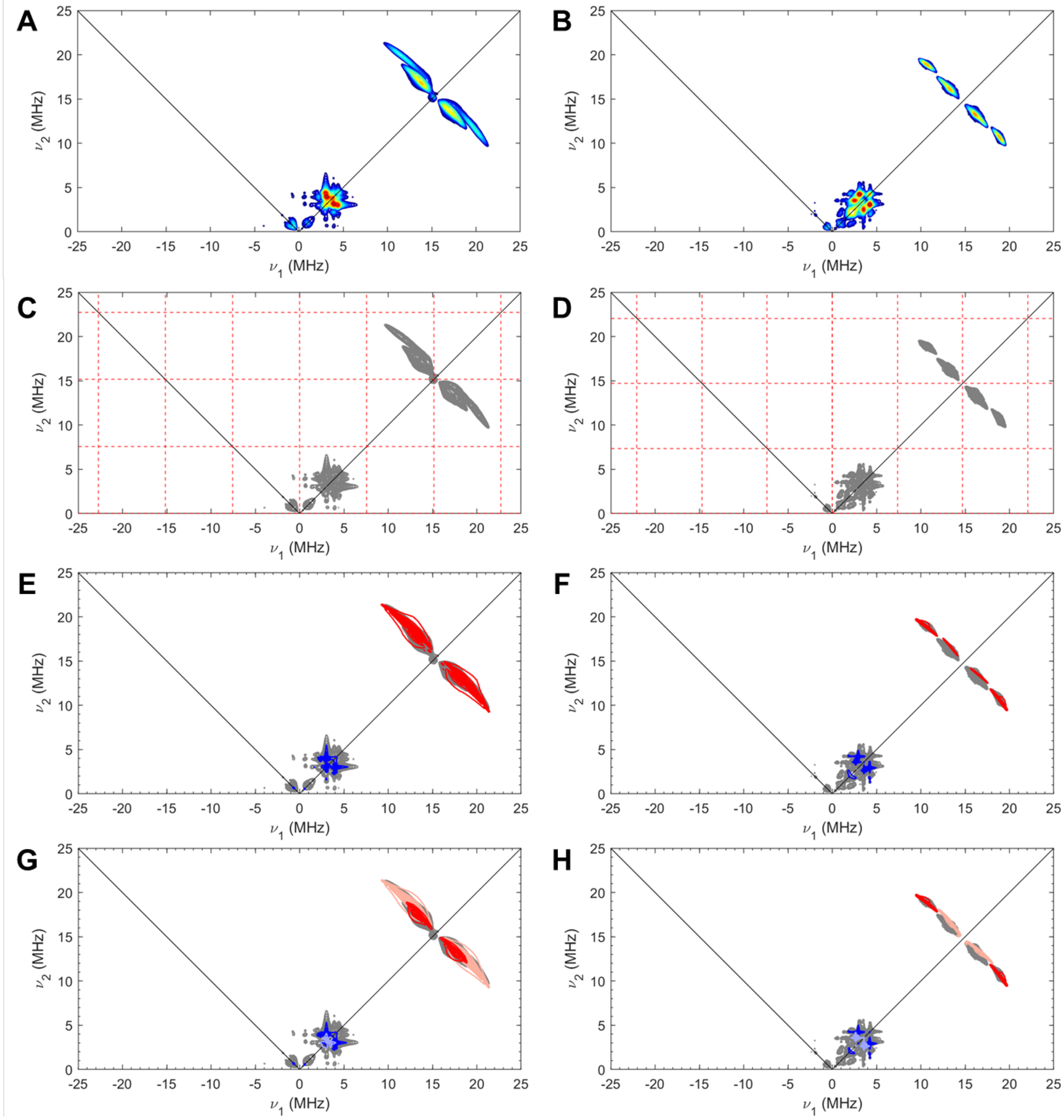

Figure S31. HYSCORE spectra for control sample at 3584 and 3436 G. (A,B) Frequency spectra for control sample at (A) 3584 and (B) 3436 G. (C,D) Frequency spectra with blind spots (dashed, red lines) for (C) 3584 and (D) 3436 G. (E,F) Simulated frequency spectra with ^{1}H (red) and ^{14}N (blue) features at (E) 3584 and (F) 3436 G. (G,H) Simulated frequency spectra with ^{1}H (red) and ^{14}N (blue) features at (G) 3584 and (H) 3436 G showing individual classes of nuclei. Experimental parameters were 9.7322 GHz, τ of 132 ns (3584 G) or 136 ns (3436 G), spacing (dt) of 12 ns for both time axes, and initial time values (t1 & t2) of 100 ns.

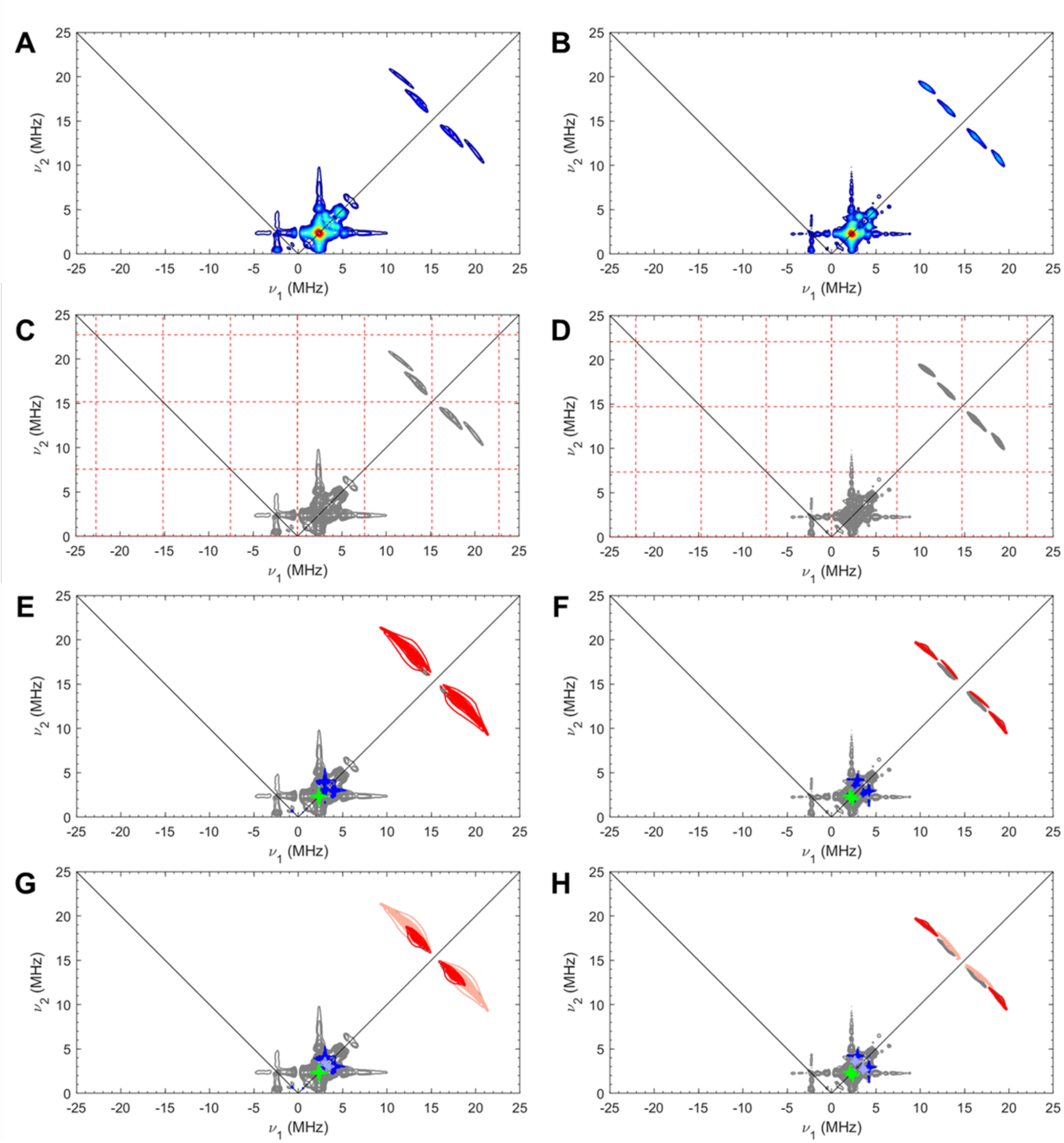

Figure S32. HYSCORE for deuterated (t=48 h) sample at 3584 and 3436 G. (A,B) Frequency spectra for deuterated (t=48 h) sample at (A) 3584 and (B) 3436 G. (C,D) Frequency spectra with blind spots (dashed, red lines) for (C) 3584 and (D) 3436 G. (E,F) Simulated frequency spectra with ^{1}H (red), ^{14}N (blue), and ^{2}H (green) features at (E) 3584 and (F) 3436 G. (G,H) Simulated frequency spectra with ^{1}H (red), ^{14}N (blue), and ^{2}H (green) features at (G) 3584 and (H) 3436 G showing individual classes of nuclei. Experimental parameters were 9.7328 (3584 G) or 9.7329 (3436 G) GHz, τ of 132 ns (3584 G) or 136 ns (3436 G), spacing (dt) of 12 ns for both time axes, and initial time values (t1 & t2) of 100 ns.

Table S4. HYSCORE and Three-pulse ESEEM Simulation Parameters. Initial simulation was informed by previous HYSCORE data on Fe_2S_2 proteins with ^{1}H coupling[8] and ^{14}N coupling[9,10].

Nucleus	A [MHz]	A_{iso} [MHz]	T [MHz]	eeQq/h [MHz]	η	Notes
**H** $^{\alpha}$	[10.3 -1.2 0.7]	3.3	[7.0 -4.5 -2.6]	-	-	
**H** $^{\beta}$	[12.6 -2.5 -1.4]	2.9	[9.7 -5.4 -4.3]	-	-	
**N** $^{\gamma,\delta}$	[0.70 0.70 1.15]	0.85	[-0.15 -0.15 0.30]	3.1	≈ 0.55	
**N** $^{\varepsilon,\zeta}$	[0 0 0.75]	0.25	[-0.25 -0.25 0.50]	2.65	≈ 0.5	
^{2}**H**	[-0.2 -0.2 0.4]	0	[-0.2 -0.2 0.4]	-	-	Deuterated sample, t = 48 h.

$^{\alpha}$ Relative to the *g*-tensor, the hyperfine ^{1}H hyperfine tensor is oriented (α, β, γ) = (0°, 105°, 0°).

$^{\beta}$ Relative to the *g*-tensor, the hyperfine ^{1}H hyperfine tensor is oriented (α, β, γ) = (0°, 20°, 0°).

$^{\gamma}$ Relative to the *g*-tensor, the hyperfine ^{14}N hyperfine tensor is oriented (α, β, γ) = (0°, 160°, 60°).

$^{\delta}$ Relative to the *g*-tensor, the hyperfine ^{14}N quadrupole tensor is oriented (α, β, γ) = (-30°, 50°, 0°).

$^{\varepsilon}$ Relative to the *g*-tensor, the hyperfine ^{14}N hyperfine tensor is oriented (α, β, γ) = (70°, 0°, 70°).

$^{\zeta}$ Relative to the *g*-tensor, the hyperfine ^{14}N quadrupole tensor is oriented (α, β, γ) = (30°, 80°, 0°).

VI. Mutants of Pdx

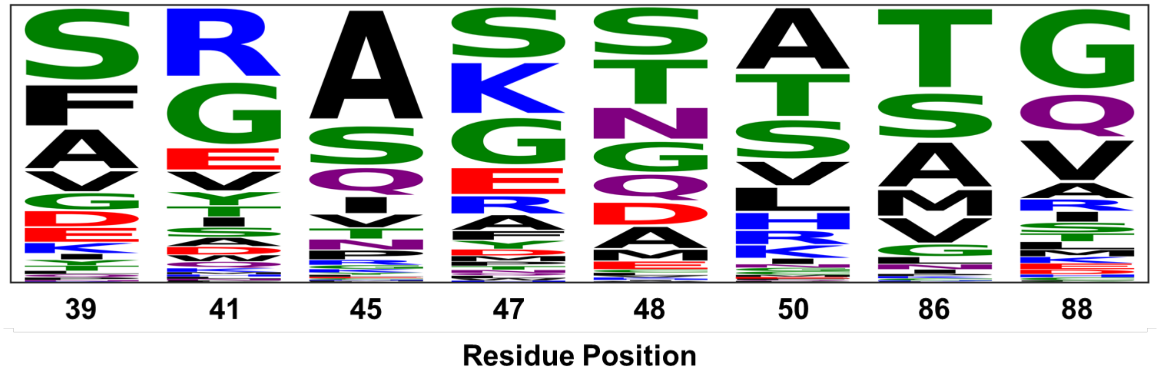

Figure S33. Most prevalent amino acids at the positions flanking coordinating cysteines in Pdx across aligned Fe_2S_2 ferredoxins. The height of each letter indicates the relative prevalence with the most prevalent amino acids at the top.

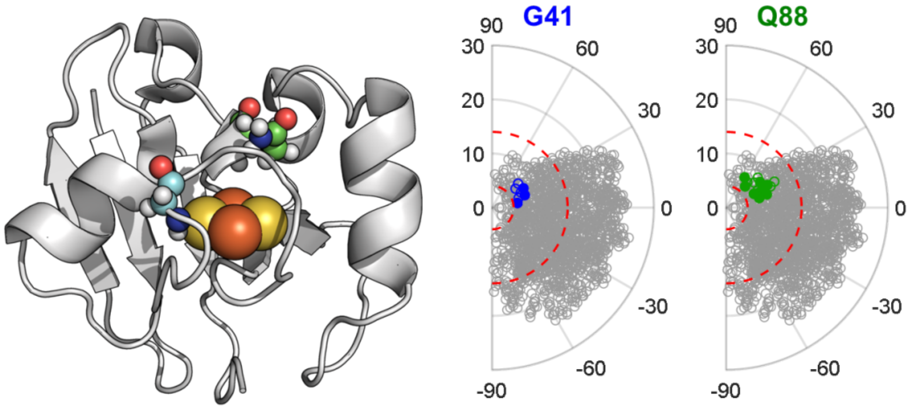

Figure S34. Reduced Pdx structure (PDB: 1XLQ) with polar plots of atomic coordinates with positions of glycine 41 (blue) and glutamine 88 (green) shown. Residue hydrogens shown as filled circles and remaining atoms as empty circles. Orientations are relative to the Fe_2S_2 cluster with the z-axis normal to the cluster plane. The red, dashed circles indicate the 4 and 14 Å shells, within which nuclei most greatly contribute to decoherence.

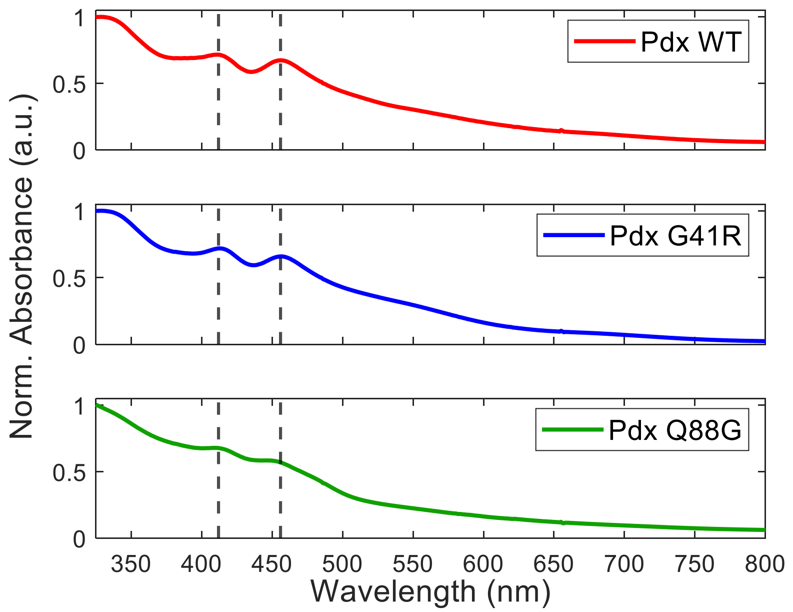

Figure S35. Normalized electronic absorption spectra of Pdx WT, G41R, and Q88G. Characteristic peaks are denoted near 412 and 456 nm. Spectra normalized at 326 nm. Purity ratios (A_{412}/A_{280}) were 0.249, 0.155, and 0.031, respectively.

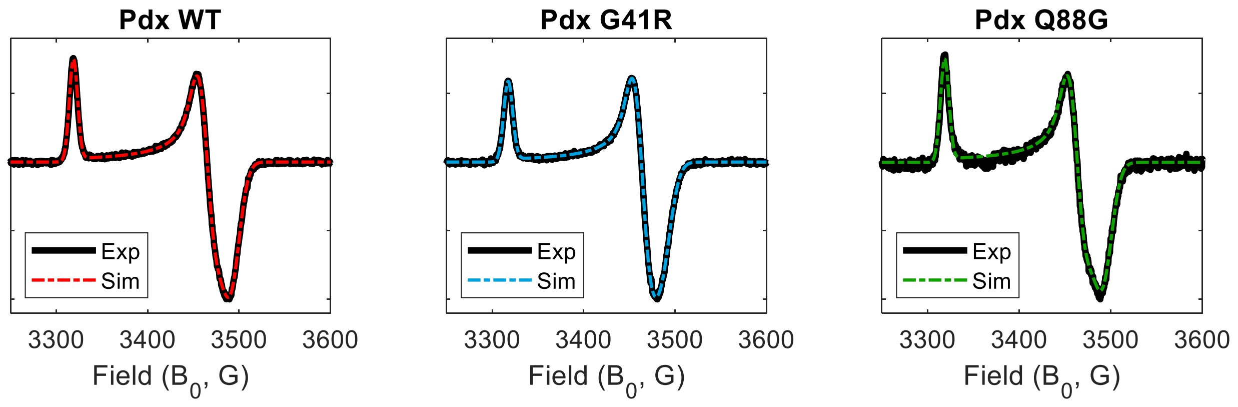

Figure S36. CW-EPR spectra (black, points) collected at 77 K with simulations (color, dashed line) for Pdx WT and mutants (G41R and Q88G). Pdx WT and G41R spectra were collected with 3 scans and 1 G modulation amplitude, and Pdx Q88G spectrum was collected with 9 scans and 1 G modulation amplitude.

Table S5. CW-EPR simulation parameters for Pdx WT and mutants (G41R and Q88G) with *g* strain.

**Pdx**	**$g_\perp$ (x, y)**	**$g_\parallel$ (z)**	**g-Strain(1)**	**g-Strain(2)**	**g-Strain(3)**
WT	(1.9222, 1.9369)	2.0221	0.012652	0.011259	0.0064558
G41R	(1.9261, 1.9380)	2.0234	0.013829	0.010818	0.0070727
Q88G	(1.9216, 1.9375)	2.0218	0.013061	0.011234	0.0066896

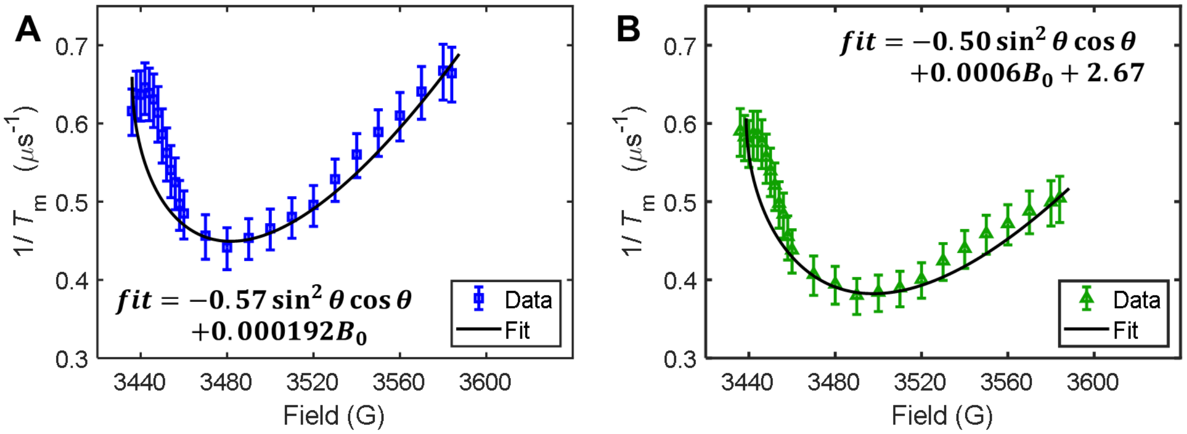

Figure S37. Fitting of mutant 1/T_m field dependence. (A) Fitting for Pdx G41R (blue). (B) Fitting for Q88G (green).

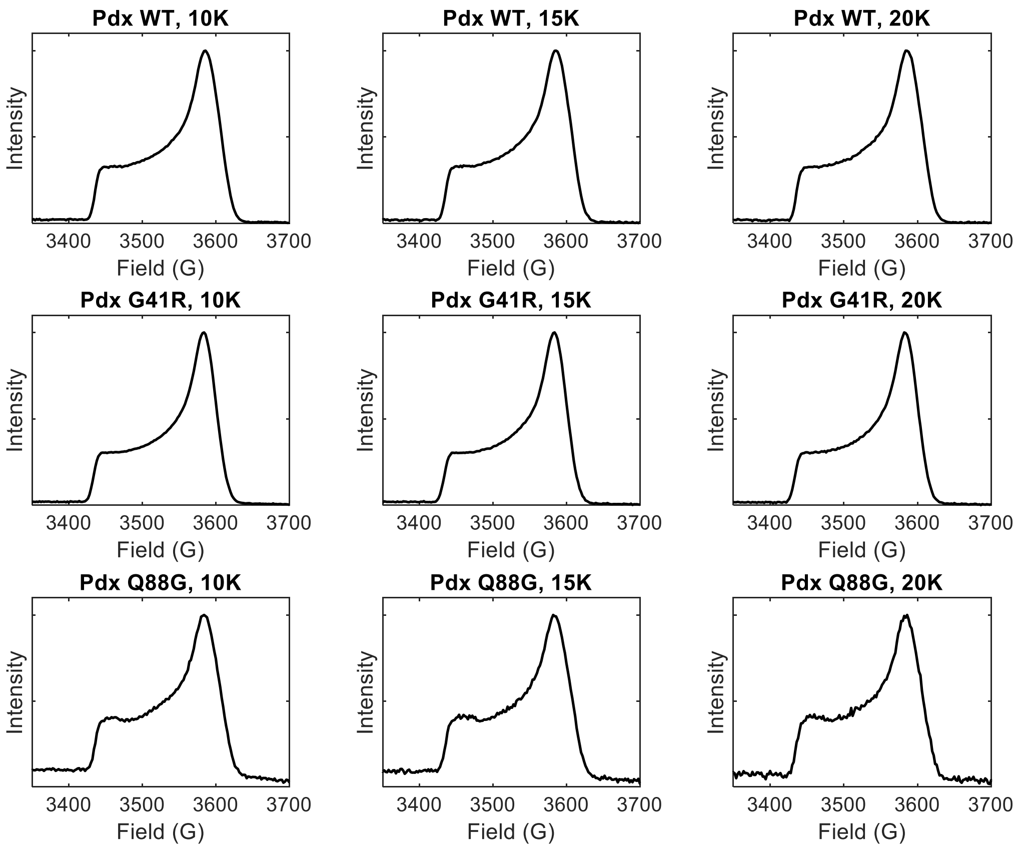

Figure S38. Echo-detected field sweeps of Pdx mutant samples at 10, 15, and 20 K at 9.73 GHz. Intensity are normalized and in arbitrary units.

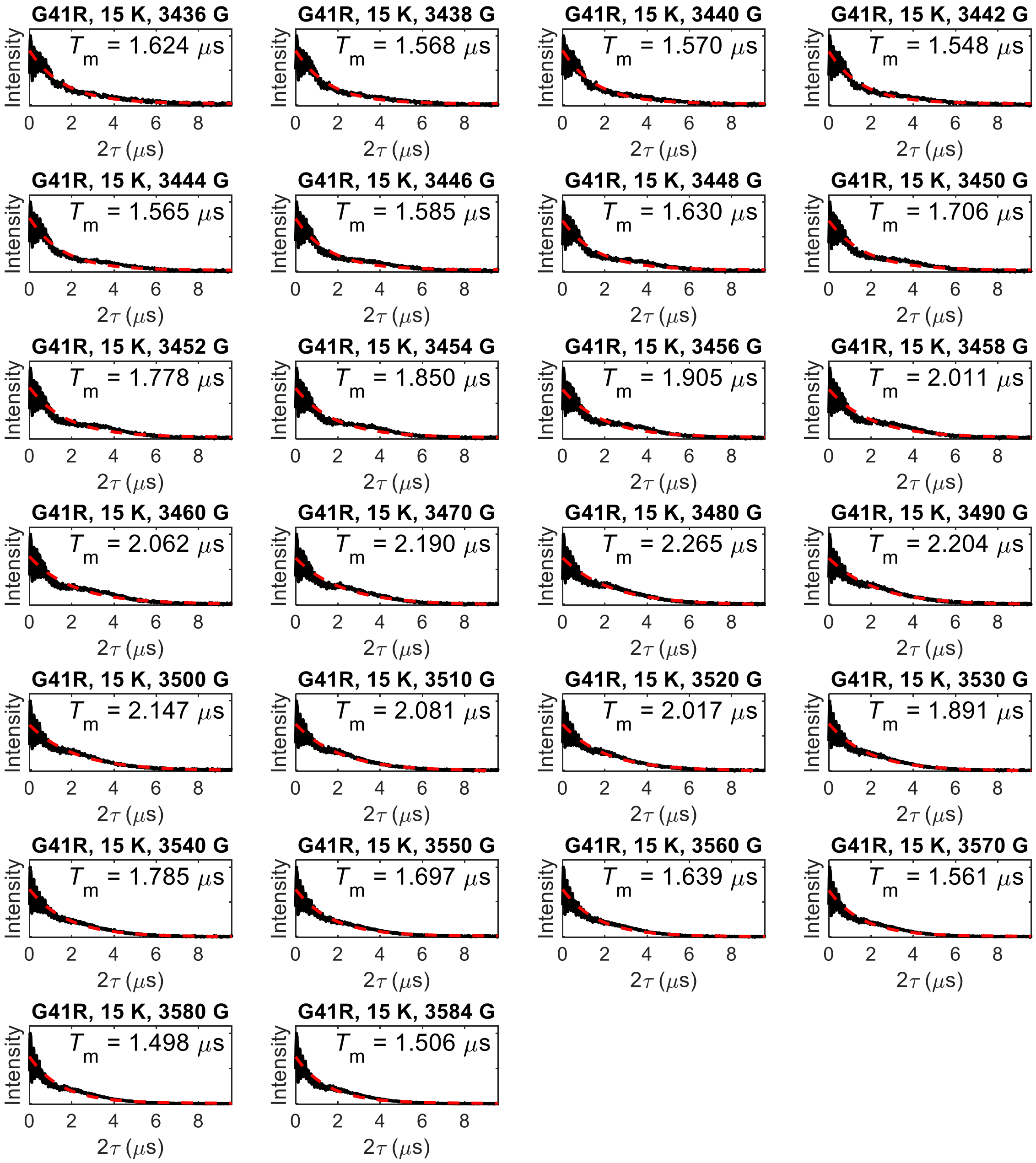

Figure S39. Two-pulse decays for Pdx G41R at 15 K and 9.73 GHz at fields from 3436 to 3584 G. Data are shown as black, solid lines with fits as red, dashed lines. Decoherence times are inlaid.

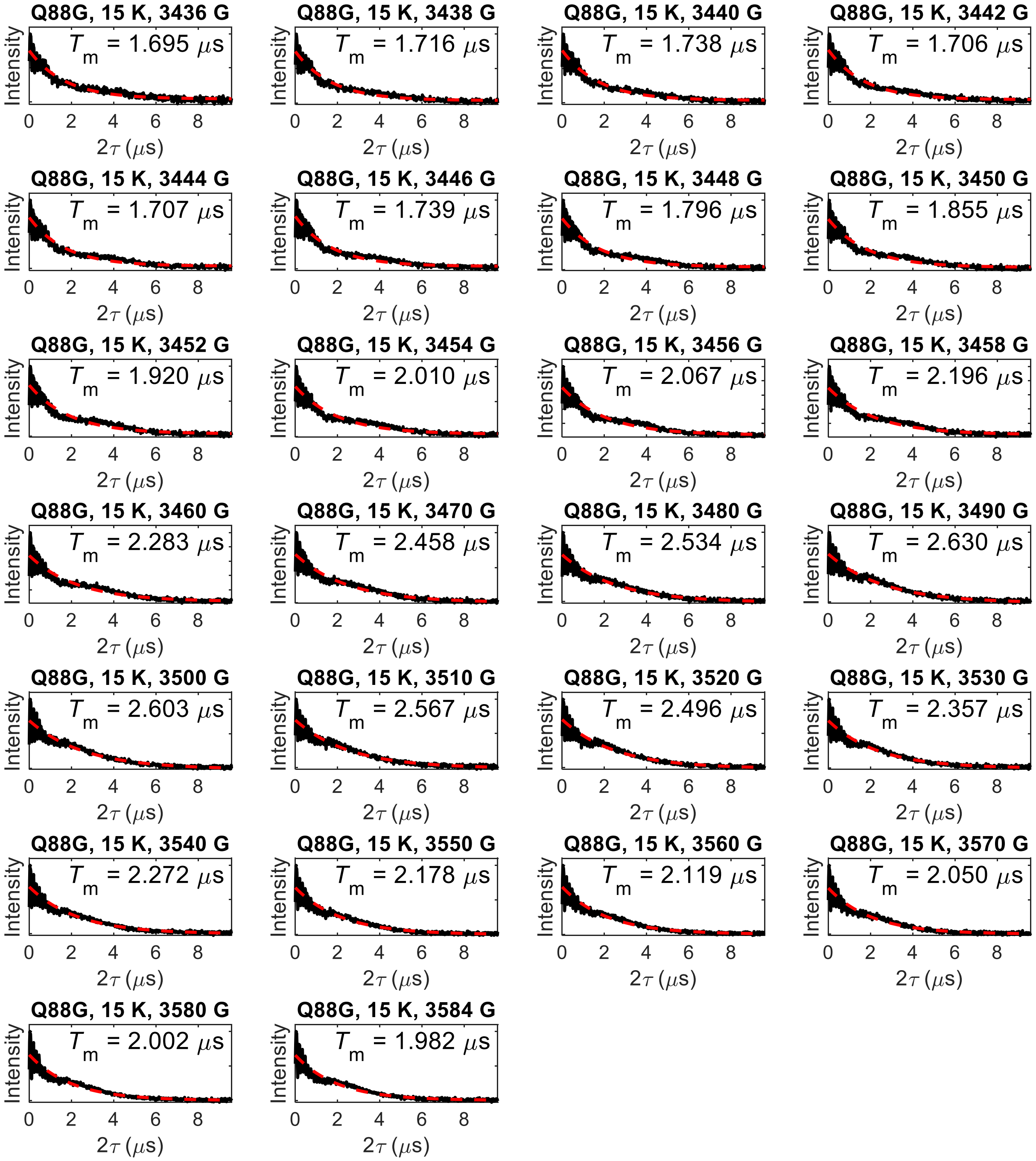

Figure S40. Two-pulse decays for Pdx Q88G at 15 K and 9.73 GHz at fields from 3436 to 3584 G. Data are shown as black, solid lines with fits as red, dashed lines. Decoherence times are inlaid.

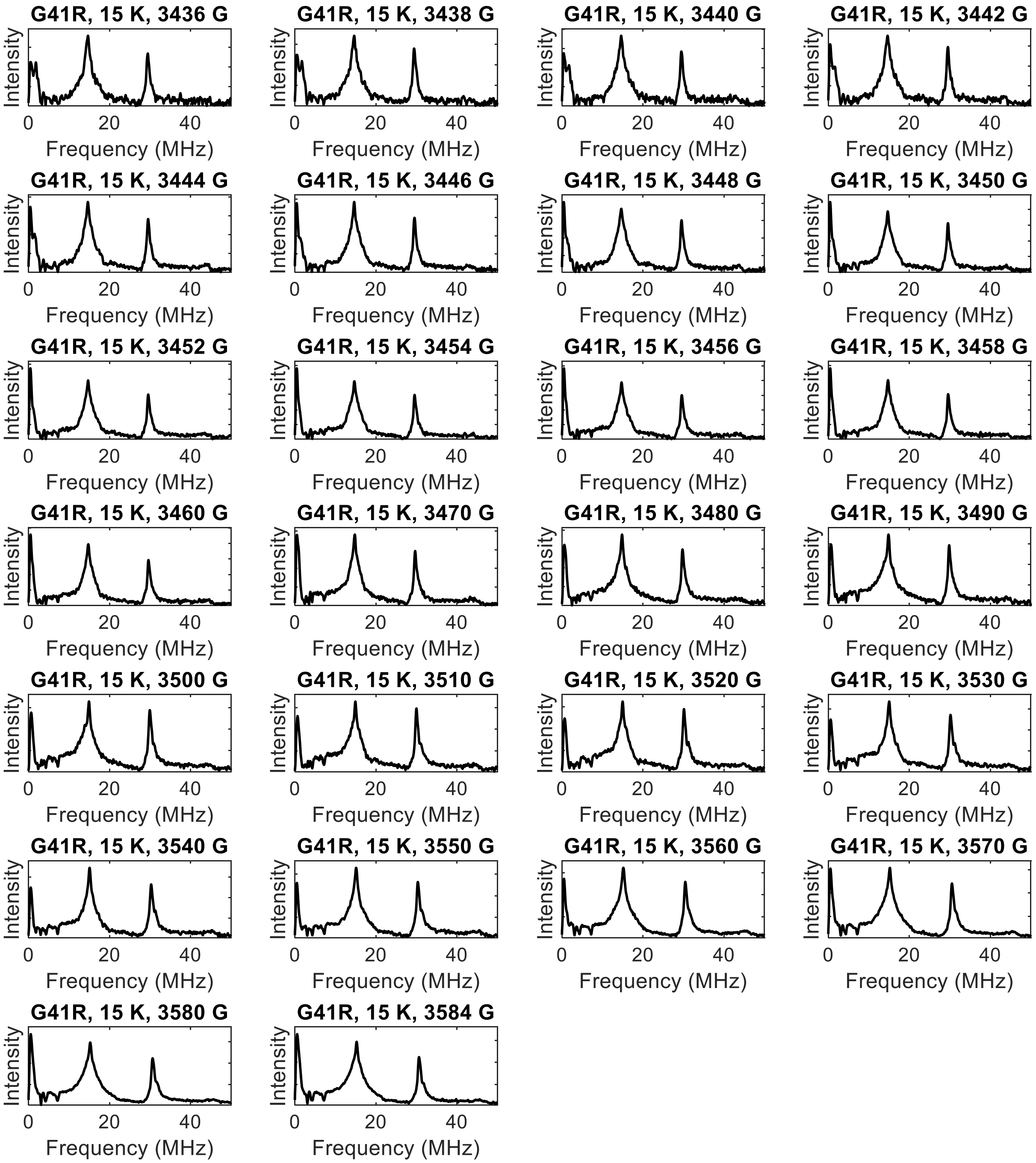

Figure S41. FFTs for Pdx G41R at 15 K and 9.73 GHz at fields from 3436 to 3584 G. Data are shown as black, solid lines following subtraction of fit from two-pulse decay traces and subsequent FFT (two-pulse ESEEM). Data (600 points) were apodized with a positive Hamming window ('ham+') and zero-filled (4800 zeros).

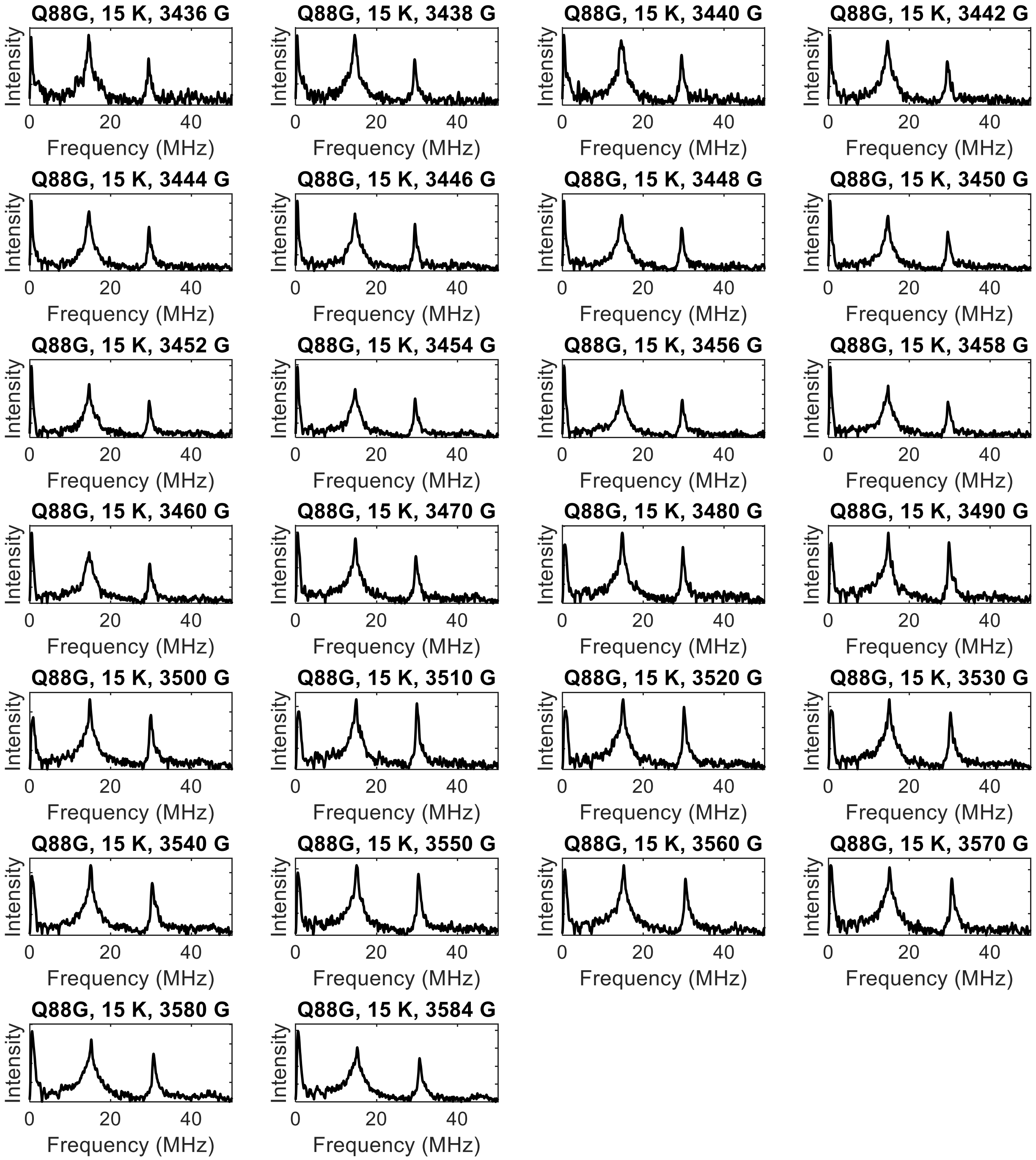

Figure S42. FFTs for Pdx Q88G at 15 K and 9.73 GHz at fields from 3436 to 3584 G. Data are shown as black, solid lines following subtraction of fit from two-pulse decay traces and subsequent FFT (two-pulse ESEEM). Data (600 points) were apodized with a positive Hamming window ('ham+') and zero-filled (4800 zeros).

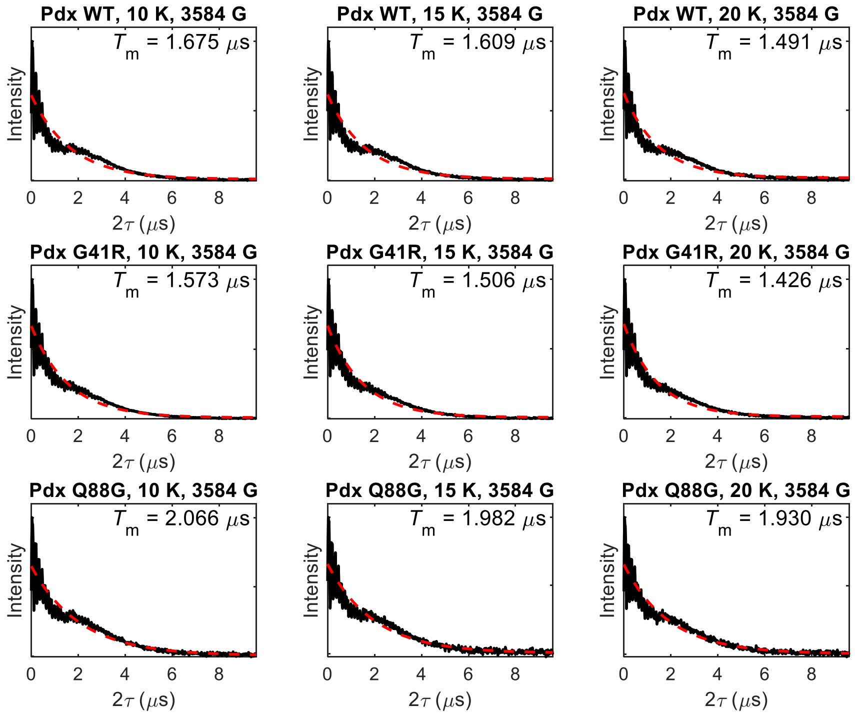

Figure S43. Two-pulse decay data (black, solid line) of Pdx samples at the perpendicular field position (3584 G) and at 10, 15, and 20 K at 9.73 GHz with accompanying fits (red, dashed line).

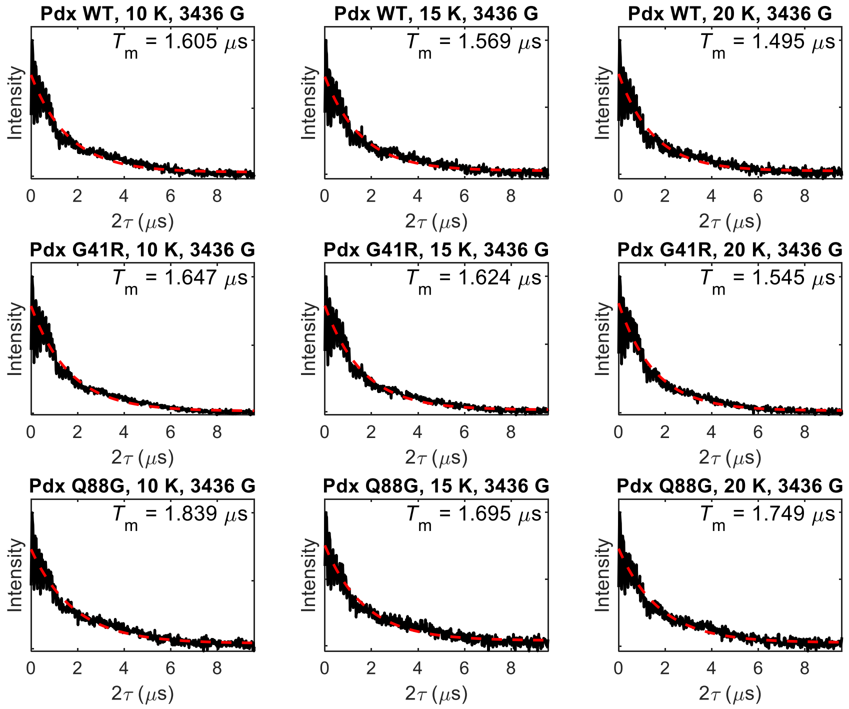

Figure S44. Two-pulse decay data (black, solid line) of Pdx samples at the parallel field position (3436 G) and at 10, 15, and 20 K at 9.73 GHz with accompanying fits (red, dashed line).

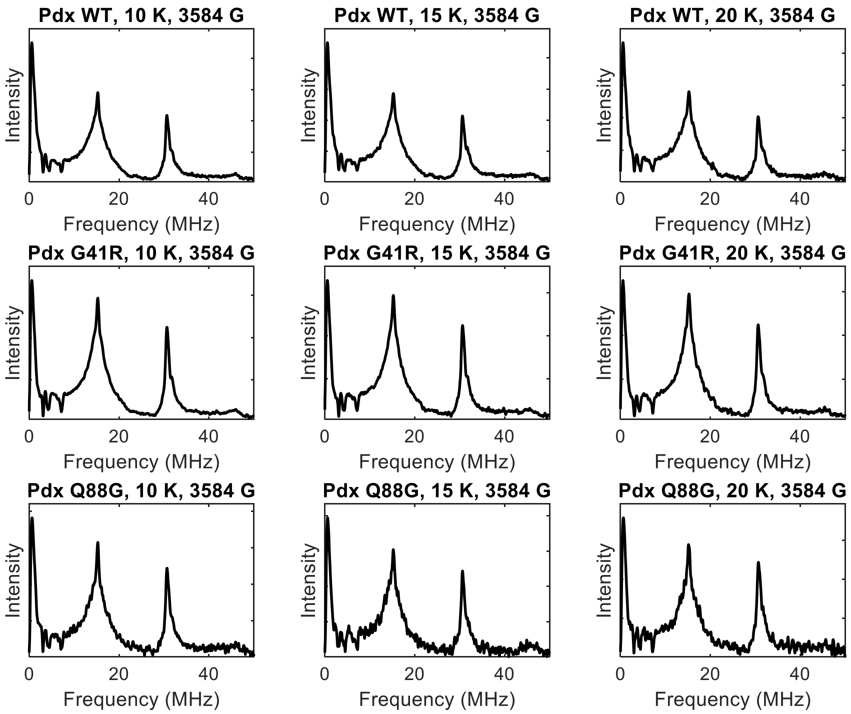

Figure S45. FFTs of fit-subtracted two-pulse decay data (two-pulse ESEEM) at the perpendicular field position (3584 G) at 10, 15, and 20 K at 9.73 GHz.

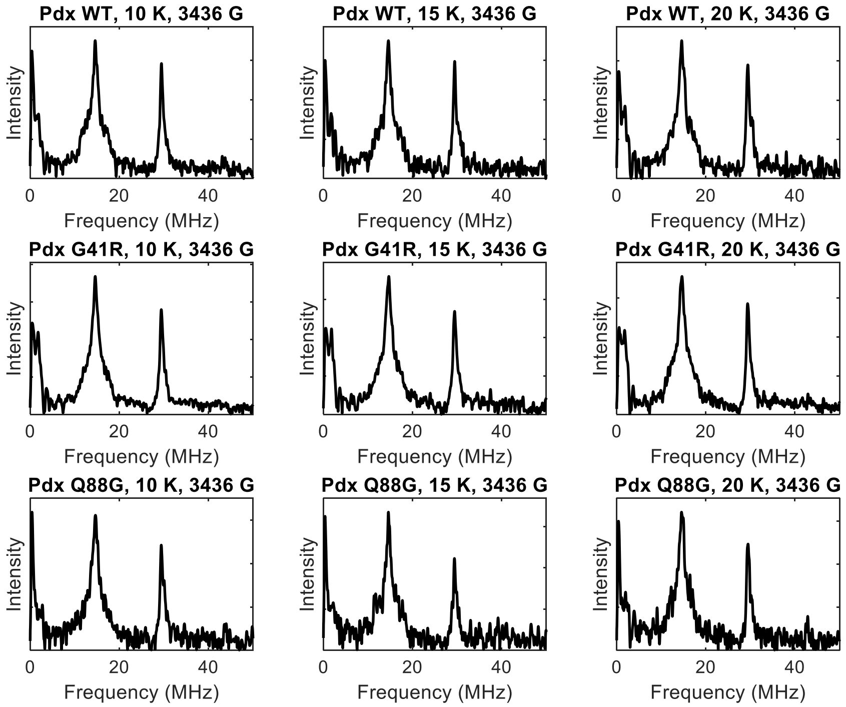

Figure S46. Fast Fourier transforms of fit-subtracted two-pulse decay data (two-pulse ESEEM) at the parallel field position (3436 G) at 10, 15, and 20 K at 9.73 GHz.

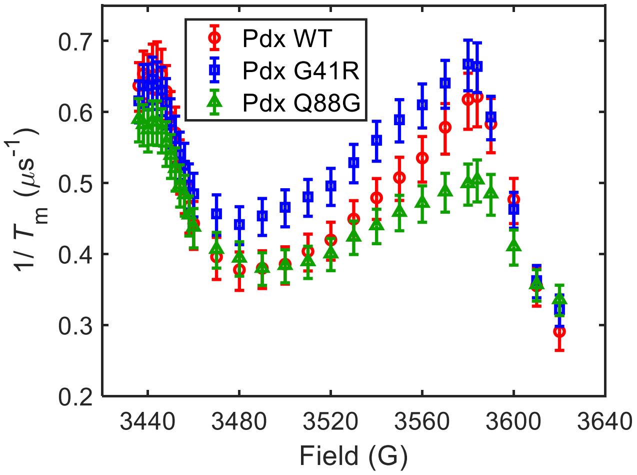

Figure S47. Overlaid T_m^{-1} field dependences, including positions beyond perpendicular orientation, of Pdx mutants with 95% confidence intervals. Decoherence rates were collected at 15 K.

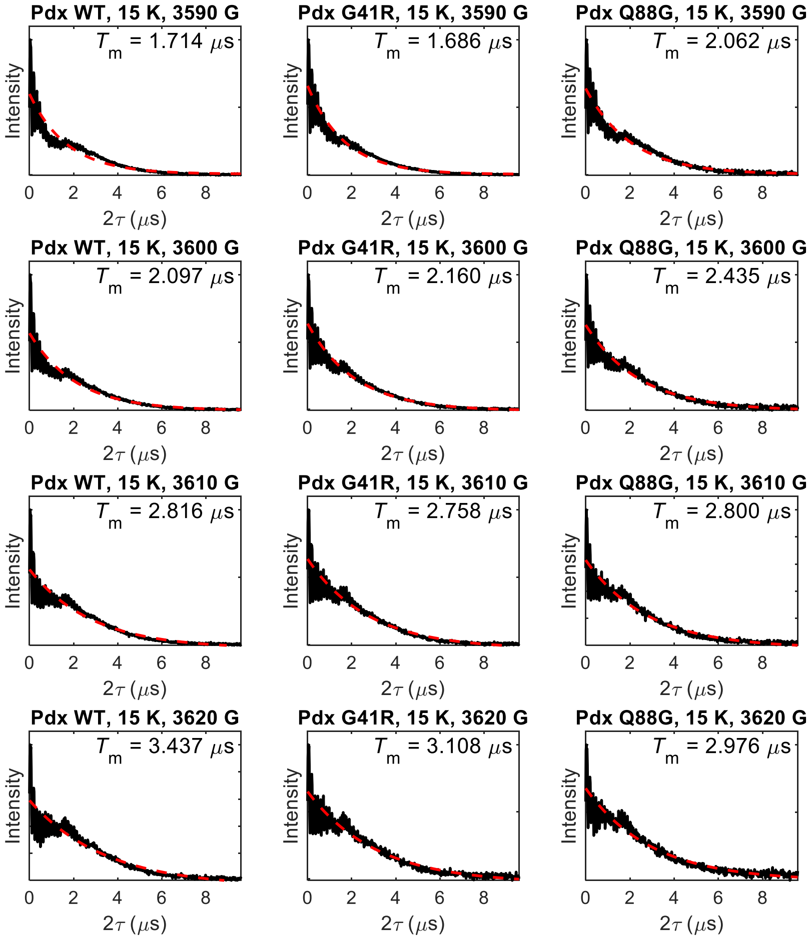

Figure S48. Two-pulse decays for Pdx WT, G41R, and Q88G at 15 K and 9.73 GHz at fields from 3590 to 3620 G. Data are shown as black, solid lines with fits as red, dashed lines. Decoherence times are inlaid.

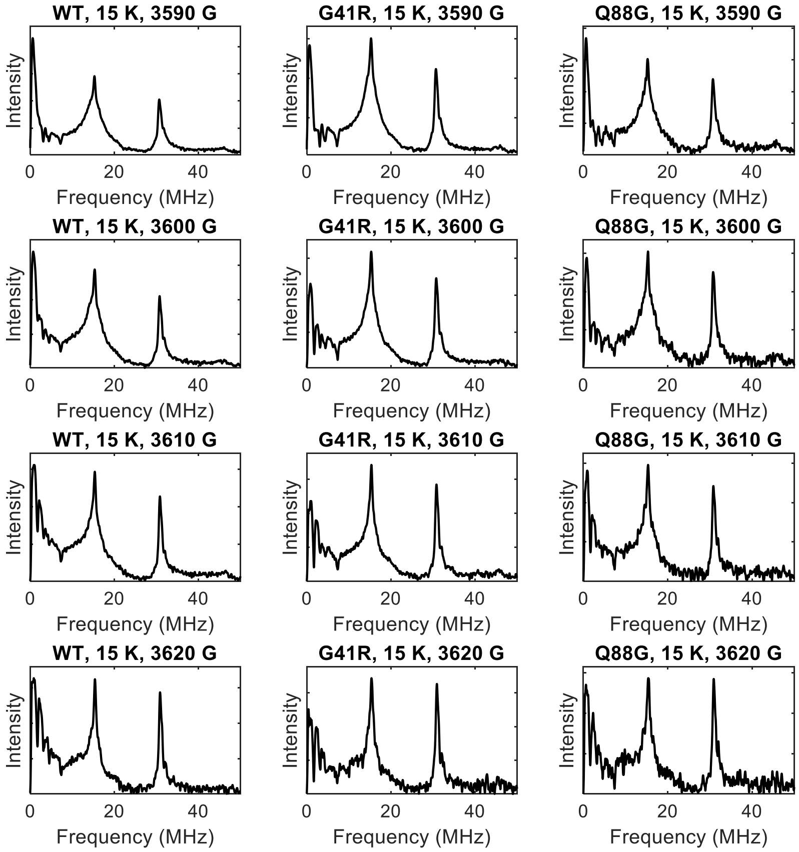

Figure S49. FFTs for Pdx WT, G41R, and Q88G at 15 K and 9.73 GHz at fields from 3590 to 3620 G. Data are shown as black, solid lines following subtraction of fit from two-pulse decay traces and subsequent FFT (two-pulse ESEEM). Data (600 points) were apodized with a positive Hamming window ('ham+') and zero-filled (4800 zeros).

VII. Expression Batches of Pdx WT

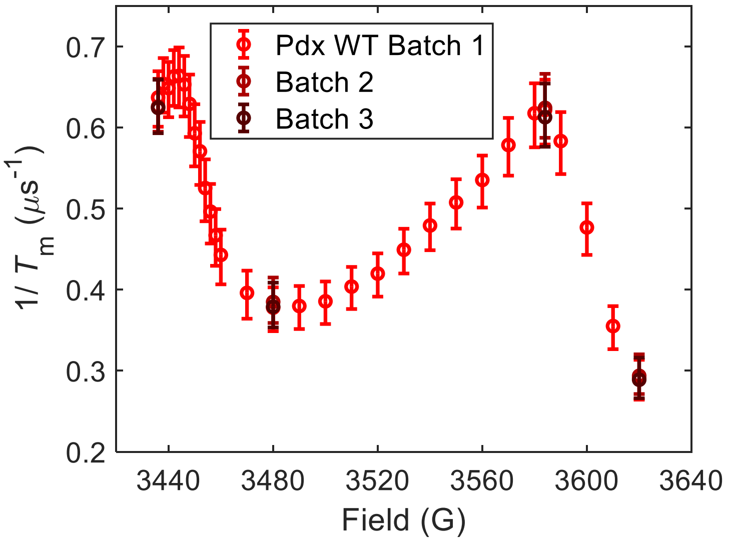

Figure S50. 1/T_m of multiple Pdx WT expression batches. Decoherence rates at various fields and 15 K for three expression batches of Pdx WT with 95% confidence intervals.

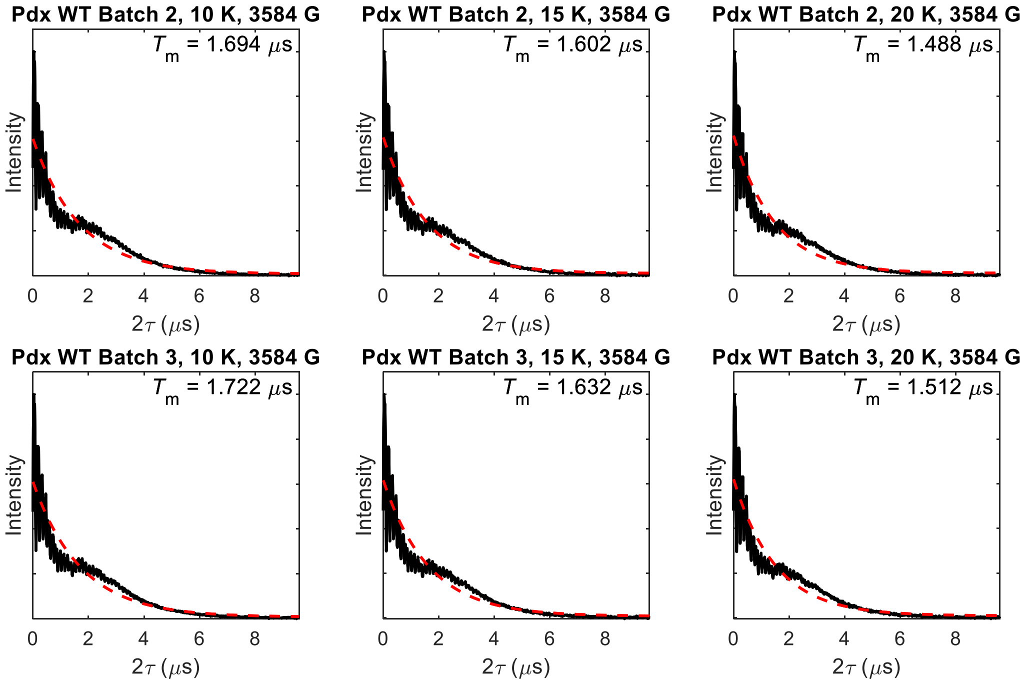

Figure S51. Two-pulse decay data (black, solid line) of Pdx WT batches at the perpendicular field position (3584 G) and at 10, 15, and 20 K at 9.73 GHz with accompanying fits (red, dashed line).

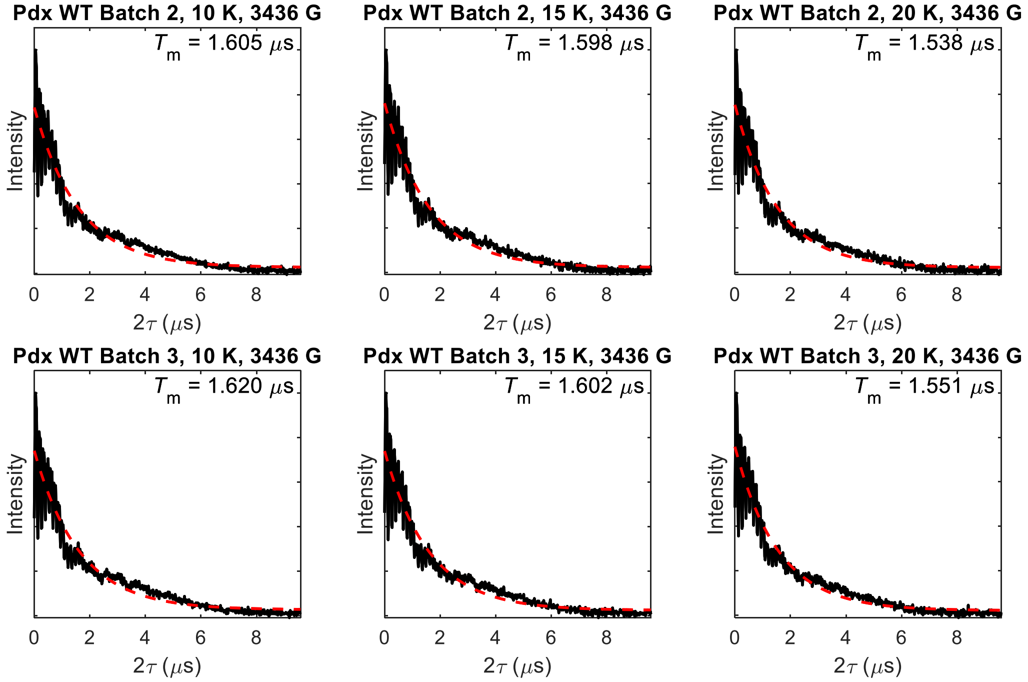

Figure S52. Two-pulse decay data (black, solid line) of Pdx WT batches at the parallel field position (3436 G) and at 10, 15, and 20 K at 9.73 GHz with accompanying fits (red, dashed line).

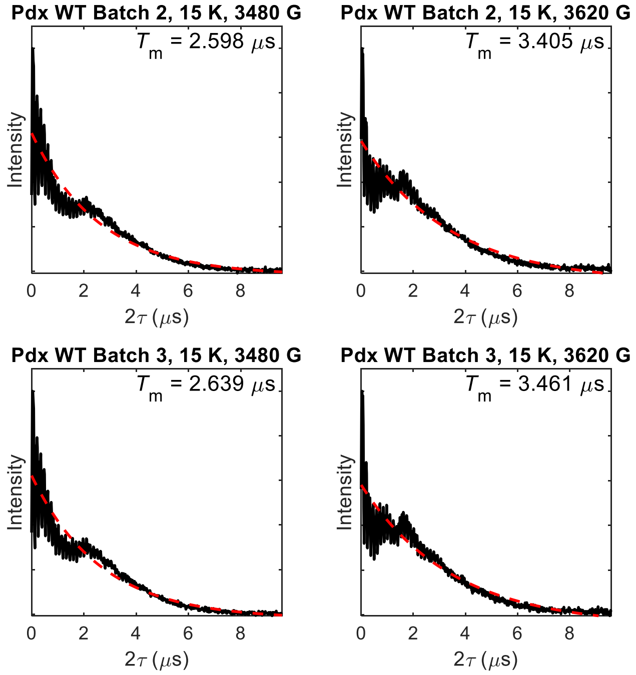

Figure S53. Two-pulse decay data (black, solid line) of Pdx WT batches at an intermediate field position (3480 G) and at 15 K at 9.73 GHz with accompanying fits (red, dashed line).

VIII. Tabulated T_m values

Table S6. Tabulated T_m values at 10 K.

	T_m (μs)	
**Sample**	***Perpendicular***	***Parallel***
*Conditions Set #1**		
Pdx WT, 0 M urea	1.763	1.617
Pdx WT, 1 M urea	1.381	1.252
Pdx WT, 2 M urea	1.357	1.208
Pdx WT, 4 M urea	1.437	1.218
*Conditions Set #2***		
Pdx WT	1.675	1.605
Pdx G41R	1.573	1.647
Pdx Q88G	2.066	1.839
*Conditions Set #3****		
Pdx WT, control (prot.)	1.527	1.432
Pdx WT, deuterated, t=0 h	1.910	1.697
Pdx WT, deut., t=0.1 h	2.819	1.874
Pdx WT, deut., t=0.5 h	2.704	2.014
Pdx WT, deut., t=1 h	2.654	2.092
Pdx WT, deut., t=6 h	2.907	2.078
Pdx WT, deut., t=24 h	2.773	2.295
Pdx WT, deut., t=48 h	3.046	2.147

* 50 mM KPi, 100 mM KCl, 0-4 M urea, 10% glycerol, pH 7.5
** 50 mM KPi, 100 mM KCl, 10% glycerol, pH 7.5
*** 50 mM KPi, 1000 mM KCl, 10% glycerol, pH 7.5
or 50 mM KPi, 1000 mM KCl, 10% d8-glycerol, pD = 7.5

Table S7. Tabulated T_m values at 15 K.

	T_m (μs)	
**Sample**	***Perpendicular***	***Parallel***
*Conditions Set #1**		
Pdx WT, 0 M urea	1.684	1.578
Pdx WT, 1 M urea	1.302	1.211
Pdx WT, 2 M urea	1.309	1.199
Pdx WT, 4 M urea	1.368	1.206
*Conditions Set #2***		
Pdx WT	1.609	1.569
Pdx G41R	1.506	1.624
Pdx Q88G	1.982	1.695
*Conditions Set #3****		
Pdx WT, control (prot.)	1.429	1.406
Pdx WT, deuterated, t=0 h	1.823	1.658
Pdx WT, deut., t=0.1 h	2.874	1.952
Pdx WT, deut., t=0.5 h	2.483	2.028
Pdx WT, deut., t=1 h	2.532	2.022
Pdx WT, deut., t=6 h	2.771	2.051
Pdx WT, deut., t=24 h	2.708	2.106
Pdx WT, deut., t=48 h	2.879	2.126

* 50 mM KPi, 100 mM KCl, 0-4 M urea, 10% glycerol, pH 7.5
** 50 mM KPi, 100 mM KCl, 10% glycerol, pH 7.5
*** 50 mM KPi, 1000 mM KCl, 10% glycerol, pH 7.5
or 50 mM KPi, 1000 mM KCl, 10% d8-glycerol, pD = 7.5

Table S8. Tabulated T_m values at 20 K.

	T_m (μs)	
**Sample**	***Perpendicular***	***Parallel***
*Conditions Set #1**		
Pdx WT, 0 M urea	1.594	1.519
Pdx WT, 1 M urea	1.258	1.186
Pdx WT, 2 M urea	1.267	1.136
Pdx WT, 4 M urea	1.290	1.177
*Conditions Set #2***		
Pdx WT	1.491	1.495
Pdx G41R	1.426	1.545
Pdx Q88G	1.930	1.749
*Conditions Set #3****		
Pdx WT, control (prot.)	2.368	1.356
Pdx WT, deuterated, t=0 h	1.728	1.618
Pdx WT, deut., t=0.1 h	2.730	2.050
Pdx WT, deut., t=0.5 h	2.344	1.894
Pdx WT, deut., t=1 h	2.431	2.187
Pdx WT, deut., t=6 h	2.553	1.984
Pdx WT, deut., t=24 h	2.467	2.113
Pdx WT, deut., t=48 h	2.653	2.033

* 50 mM KPi, 100 mM KCl, 0-4 M urea, 10% glycerol, pH 7.5

** 50 mM KPi, 100 mM KCl, 10% glycerol, pH 7.5

*** 50 mM KPi, 1000 mM KCl, 10% glycerol, pH 7.5
or 50 mM KPi, 1000 mM KCl, 10% d8-glycerol, pD = 7.5

Table S9. Tabulated T_m values at 15 K for Pdx WT, G41R, and Q88G field dependences.

	T_m (μs)		
**Field (G)**	***Pdx WT*****	***Pdx G41R*****	***Pdx Q88G*****
3436	1.569	1.624	1.695
3438	1.529	1.568	1.716
3440	1.542	1.570	1.738
3442	1.508	1.548	1.706
3444	1.506	1.565	1.707
3446	1.531	1.585	1.739
3448	1.589	1.630	1.796
3450	1.687	1.706	1.855
3452	1.752	1.778	1.920
3454	1.903	1.850	2.010
3456	2.014	1.905	2.067
3458	2.141	2.011	2.196
3460	2.258	2.062	2.283
3470	2.525	2.190	2.458
3480	2.647	2.265	2.534
3490	2.633	2.204	2.630
3500	2.593	2.147	2.603
3510	2.477	2.081	2.567
3520	2.382	2.017	2.496
3530	2.226	1.891	2.357
3540	2.087	1.785	2.272
3550	1.970	1.697	2.178
3560	1.868	1.639	2.119
3570	1.729	1.561	2.050
3580	1.619	1.498	2.002
3584	1.609	1.506	1.982
3590	1.714	1.686	2.062
3600	2.097	2.160	2.435
3610	2.816	2.758	2.800
3620	3.437	3.106	2.976

** 50 mM KPi, 100 mM KCl, 10% glycerol, pH 7.5

IX. References

(1) Kuznetsov, V. Y.; Blair, E.; Farmer, P. J.; Poulos, T. L.; Pifferitti, A.; Sevrioukova, I. F. The Putidaredoxin Reductase-Putidaredoxin Electron Transfer Complex. *Journal of Biological Chemistry* **2005**, *280* (16), 16135–16142. https://doi.org/10.1074/jbc.M500771200.

(2) Stoll, S.; Schweiger, A. EasySpin, a Comprehensive Software Package for Spectral Simulation and Analysis in EPR. *Journal of Magnetic Resonance* **2006**, *178* (1), 42–55. https://doi.org/10.1016/j.jmr.2005.08.013.

(3) Du, J.-L.; More, K. M.; Eaton, S. S.; Eaton, G. R. Orientation Dependence of Electron Spin Phase Memory Relaxation Times in Copper(II) and Vanadyl Complexes in Frozen Solution. *Isr. J. Chem.* **1992**, *32* (2–3), 351–355. https://doi.org/10.1002/ijch.199200041.

(4) Kevan, L.; Schwartz, R. N. *Time Domain Electron Spin Resonance*; Wiley: New York, 1979.

(5) Husted, R.; Du, J.; Eaton, G. R.; Eaton, S. S. Temperature and Orientation Dependence of Electron Spin Relaxation in Molybdenum(V) Porphyrins. *Magnetic Reson in Chemistry* **1995**, *33* (13). https://doi.org/10.1002/mrc.1260331312.

(6) Schweiger, A.; Jeschke, G. *Principles of Pulse Electron Paramagnetic Resonance*; Oxford University Press: Oxford, UK ; New York, 2001.

(7) Hearshen, D. O.; Hagen, W. R.; Sands, R. H.; Grande, H. J.; Crespi, H. L.; Gunsalus, I. C.; Dunham, W. R. An Analysis of g Strain in the EPR of Two [2Fe-2S] Ferredoxins. Evidence for a Protein Rigidity Model. *Journal of Magnetic Resonance (1969)* **1986**, *69* (3), 440–459. https://doi.org/10.1016/0022-2364(86)90156-3.

(8) Abdalla, J. A. B.; Bowen, A. M.; Bell, S. G.; Wong, L. L.; Timmel, C. R.; Harmer, J. Characterisation of the Paramagnetic [2Fe–2S]+ Centre in Palustrisredoxin-B (PuxB) from Rhodopseudomonas Palustris CGA009: G-Matrix Determination and Spin Coupling Analysis. *Phys. Chem. Chem. Phys.* **2012**, *14* (18), 6526. https://doi.org/10.1039/c2cp24112a.

(9) Shergill, J. K.; Joannou, C. L.; Mason, J. R.; Cammack, R. Coordination of the Rieske-Type [2Fe-2S] Cluster of the Terminal Iron-Sulfur Protein of Pseudomonas Putida Benzene 1,2-Dioxygenase, Studied by One- and Two-Dimensional Electron Spin-Echo Envelope Modulation Spectroscopy. *Biochemistry* **1995**, *34* (51), 16533–16542. https://doi.org/10.1021/bi00051a001.

(10) Maly, T.; Grgic, L.; Zwicker, K.; Zickermann, V.; Brandt, U.; Prisner, T. Cluster N1 of Complex I from Yarrowia Lipolytica Studied by Pulsed EPR Spectroscopy. *J Biol Inorg Chem* **2006**, *11* (3), 343–350. https://doi.org/10.1007/s00775-006-0081-1.
